# Improving the prediction of Spreading Through Air Spaces (STAS) in primary lung cancer with a dynamic dual-delta hybrid machine learning model: a multicenter cohort study

**DOI:** 10.1186/s40364-023-00539-9

**Published:** 2023-11-23

**Authors:** Weiqiu Jin, Leilei Shen, Yu Tian, Hongda Zhu, Ningyuan Zou, Mengwei Zhang, Qian Chen, Changzi Dong, Qisheng Yang, Long Jiang, Jia Huang, Zheng Yuan, Xiaodan Ye, Qingquan Luo

**Affiliations:** 1 Shanghai Lung Cancer Center, Shanghai Chest Hospital, Shanghai Jiao Tong University School of Medicine, Shanghai, 200030 China; 2 Department of Radiology, Zhongshan Hospital, Fudan University, Shanghai, 200032 China; 3 Shanghai Institute of Medical Imaging, Shanghai, 200032 China; 4 Department of Cancer Center, Zhongshan Hospital, Fudan University, Shanghai, 200032 China; 5 School of Medicine, Shanghai Jiao Tong University School of Medicine, Shanghai, 200025 China; 6 Department of Radiology, Ruijin Hospital, Shanghai Jiao Tong University School of Medicine, Shanghai, 200025 China; 7 Department of Bioengineering, School of Engineering and Science, University of Pennsylvania, Philadelphia, 19104 USA; 8 School of Integrated Circuits & Beijing National Research On Information Science and Technology (BNRist), Tsinghua University, Beijing, 100084 China; 9 Department of Radiology, Shanghai Ninth People’s Hospital, Shanghai Jiao Tong University School of Medicine, Shanghai, 200011 China; 10 Department of Radiology, Shanghai Chest Hospital, Shanghai Jiao Tong University, Shanghai, 200030 China

**Keywords:** Deep learning, Radiomics, Spreading through air spaces (STAS), Lung cancer

## Abstract

**Background:**

Reliable pre-surgical prediction of spreading through air spaces (STAS) in primary lung cancer is essential for precision treatment and surgical decision-making. We aimed to develop and validate a dual-delta deep-learning and radiomics model based on pretreatment computed tomography (CT) image series to predict the STAS in patients with lung cancer.

**Method:**

Six hundred seventy-four patients with pre-surgery CT follow-up scans (with a minimum interval of two weeks) and primary lung cancer diagnosed by surgery were retrospectively recruited from three Chinese hospitals. The training cohort and internal validation cohort, comprising 509 and 76 patients respectively, were selected from Shanghai Chest Hospital; the external validation cohorts comprised 36 and 53 patients from two other centers, respectively. Four imaging signatures (classic radiomics features and deep learning [DL] features, delta-radiomics and delta-DL features) reflecting the STAS status were constructed from the pretreatment CT images by comprehensive methods including handcrafting, 3D views extraction, image registration and subtraction. A stepwise optimized three-step procedure, including feature extraction (by DL and time-base radiomics slope), feature selection (by reproducibility check and 45 selection algorithms), and classification (32 classifiers considered), was applied for signature building and methodology optimization. The interpretability of the proposed model was further assessed with Grad-CAM for DL-features and feature ranking for radiomics features.

**Results:**

The dual-delta model showed satisfactory discrimination between STAS and non-STAS and yielded the areas under the receiver operating curve (AUCs) of 0.94 (95% CI, 0.92–0.96), 0.84 (95% CI, 0.82–0.86), and 0.84 (95% CI, 0.83–0.85) in the internal and two external validation cohorts, respectively, with interpretable core feature sets and feature maps.

**Conclusion:**

The coupling of delta-DL model with delta-radiomics features enriches information such as anisotropy of tumor growth and heterogeneous changes within the tumor during the radiological follow-up, which could provide valuable information for STAS prediction in primary lung cancer.

**Supplementary Information:**

The online version contains supplementary material available at 10.1186/s40364-023-00539-9.

## Background

Lung cancer is currently the second most commonly diagnosed malignancy with the highest mortality rate according to the GLOBOCAN study [1]. Due to its histological peculiarities, lung cancer has its own unique mode of invasion. In addition to the classic direct infiltration, lymphatic metastasis and hematogenous metastasis, another mode of invasion has been identified in recent years: spread through air space (STAS). This concept was first introduced by Kadota et al. in 2015 [2], defining it as the spread of tumor cells (as micropapillary structures, solid nests or single cells) in the airspace beyond the margins of the primary tumor. This definition was officially established by the World Health Organization (WHO) in the same year. Recent studies have shown that the presence of STAS often indicates more aggressive tumor biology, higher risk of recurrence and lymph node metastasis, and lower survival rates [3]. Furthermore, numerous clinical studies have confirmed that STAS is a high-risk factor for recurrence in sub-lobar resection and that lobectomy demonstrates a lower recurrence rate and overall survival in STAS-positive patients compared to sub-lobar resection [4, 5]. Therefore, the assessment of STAS in lung cancer can significantly influence clinical decisions such as the choice of surgical procedure, the extent of lymph node dissection, and the need for post-operative chemotherapy. Currently, the assessment of STAS relies on histological analysis after biopsy. However, due to the diversity of STAS pathology, the inconsistent criteria and the interference of intraoperative treatment with discrete artefacts [6], the sensitivity of STAS detection by Intraoperative frozen sections is currently low (55%) [7], which cannot provide reliable guidance for clinical situations, especially the surgical decision making. Therefore, new strategies for reliable preoperative STAS detection need to be developed.

Several studies have shown that STAS leads to changes in the CT presentation of lung cancer [8, 9], which correlates with the presence of notch, vascular convergence, pleural indentation, central low attenuation, ill-defined opacity, air bronchogram, and percentage of solid components, suggesting the potential value of lung radiology to predict STAS. However, there are many limitations to conventional radiological diagnostic indexes and signs, such as differences in technical parameters such as layer thickness of CT and subjective errors in reader judgement due to inconsistent and uncertain imaging criteria for STAS. With the development of radiomics, computer extraction of histological features from CT images can quantify image information in high throughput, reduce the interference of subjective judgement and improve prediction performance. Several studies have shown that radiomics can quantify tumor characteristics and predict STAS in lung cancer. Jiang et al. first applied a CT-based radiomics random forest (RF) model to predict STAS in lung adenocarcinoma and achieved an AUC of 0.754 (sensitivity of 0.880 and specificity of 0.588) [10]. The CT radiomics with plain Bayesian model developed by Chen et al. also showed good performance in predicting STAS in stage I lung adenocarcinoma preoperatively (externally validated AUC = 0.69) [11]. Tao et al. evaluated the efficacy of conventional radiomics models, Computer Vision (CV) models, 3D-CNN models and combination models in predicting STAS status in non-small cell lung cancer (NSCLC) and identified 3D-CNN as the best prediction model [12]. Liao et al. and Takehana et al. incorporated peri-tumor information into the radiomics feature extraction process and identified the peri-tumor range with the best predictive effect [13, 14]. All of these studies suggest that the radiomics is a potential pipeline for non-invasive clinical biomarker discovery for STAS (a summary provided in Additional file 1: Appendix A).

Existing studies predicted STAS with CT scan at a single timepoint. However, in today’s clinical practice, follow-up has become one of the most important and frequent clinical activities in pulmonary oncology. Physicians usually need to combine the follow-up imaging and psychological expectations of patients to design personalized examination or treatment plans for them. Therefore, how to correctly interpret and fully exploit the content of imaging information has become a key issue in the era of artificial intelligence and precision medicine. Classic radiomics or DL usually gives assessment or reference based on medical images at a certain timepoint, which does not seem to provide an adequate quantitative description of dynamic follow-up observations in the clinic. As an emerging radiomics method focusing on the dynamic tracking of characteristic changes in lesion sites over time, delta-radiomics, a system of metrics for making differences between two radiomics indexes, has been showing powerful prediction efficacy in differential diagnosis, prognosis analysis, treatment response prediction, and side effect assessment [15]. Moreover, some studies have demonstrated its increasingly important role in predicting pathological features such as malignancy and aggressiveness of pulmonary nodules [16, 17]. For example, Alahmari et al. evaluated the use of machine learning to combine delta-radiomics with conventional (non-delta) radiomics features in predicting lung nodal malignancies and found a significant performance improvement [16]. Ma et al. showed that delta-radiomics outperformed conventional radiomics in distinguishing between pre-infiltrative and invasive ground-glass nodules (GGNs) [17]. Due to the aggressive biology of STAS-positive tumor such as a higher proportion of micropapillary or solid growth characteristics with their unique aggressiveness [18], the use of delta-radiomics to dynamically monitor and describe the tumor growth and development may be more beneficial for the discriminative diagnosis of STAS. However, the traditional delta-radiomics model could be limited by the strict restriction on the follow-up time interval in most of the previous studies. Additionally, this metric system is based on the quantitative description of the image, which is less intuitive than visual models such as CNN-based DL.

**Fig. 1:**
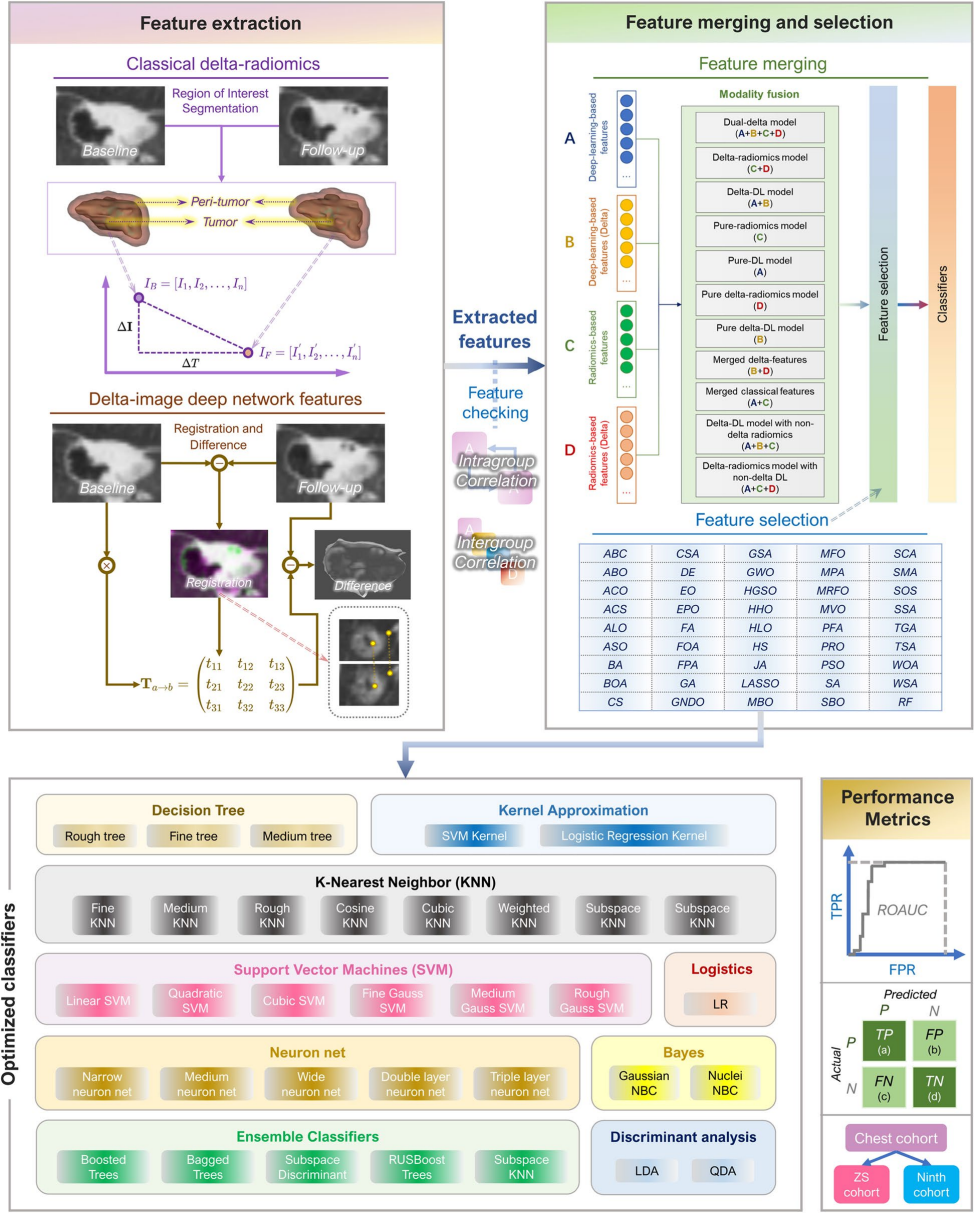
The framework of this study. This work mainly contained three steps: feature extraction by delta-radiomics and deep learning from the delta-images of lung tumors acquired by image registration, feature merging and selection where ICC values were applied to select the features with reliability and 45 methods were applied to optimize the feature set, optimized classification where 32 AI methods were tested. Model performances were evaluated by ROAUC and confusion matrix where two external cohort (ZS Cohort and Ninth Cohort) were used for cross-center validation

In this way, we developed a delta-radiomics machine learning model combined with DL networks extracting features from the post-registration subtracted images to predict STAS in primary lung cancer (Fig. 1). This work explored the feasibility of feature merging with radiomics and deep-learning. More specifically, the value of combining the features extracted by deep network (CNNs) from the subtracted images after registration and the delta-radiomics was systematically studied. We defined this approach as a dual-delta model since both the delta-radiomics based on the slope of classic radiomics indexes within a time interval and the registration-based CNN deep-features describing the difference of lung tumor between the baseline and follow-up scans are ways to detect and quantify the growth and change of lung nodules, namely the “delta” attributions of tumors. Our results demonstrated that this model is not only suitable for various follow-up intervals, but also more interpretable and intuitive than the classic DL model, which is one of the most powerful models for STAS prediction at present.

## Methods

### Patients

**Fig. 2:**
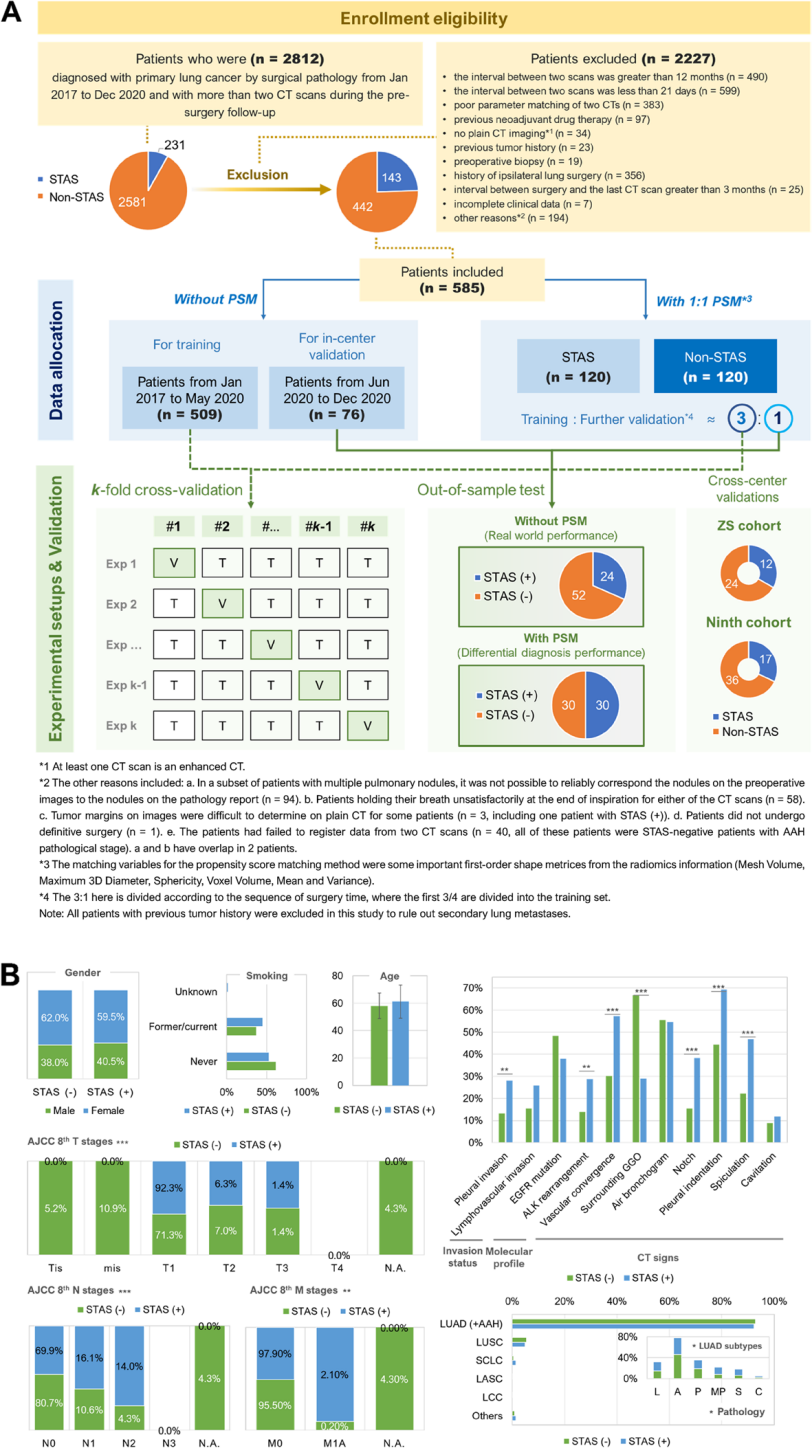
The building of CHEST cohort and its baseline information. **A** The enrollment eligibility for CHEST cohort and the data allocation in the experimental and validation setups. **B** The baseline information of the studied cohort (Gender, age, smoking history, AJCC stages, invasion status, molecular and paraffin pathology with LUAD subtypes, and the CT signs in baseline scan). Abbreviations: lepidic, L; acinar, A; papillary, P; micropapillary, MP; solid, S; complex glandular pattern, C; Not applicable, N.A.; American Joint Committee on Cancer, AJCC

This study was approved by the ethical number KS1832 from the Shanghai Chest Hospital, and all patient imaging, pathological, and clinical information was processed under anonymized conditions to fully ensure patient privacy. Under the approval of multicenter study ethics number IS2126, patient information from Zhongshan Hospital (ethical number: B2023-039) and the Ninth People’s Hospital (ethical number: SH9H-2023-T158-1) was also extracted for further cross-center analysis under the same anonymization processes. Considering the retrospective nature of this study, the ethics committee waived written informed consent from patients at all three centers. A retrospective analysis of 2,812 patients with primary lung cancer diagnosed by surgery and preoperative CT follow-up scans (with minimum interval of over 2 weeks required) from January 2017 to December 2020 at Shanghai Lung Cancer Center in Shanghai Chest Hospital was performed (Fig. 2A). Of these, 2227 patients were excluded due to the interval between two scans greater than 12 months (*n* = 490) or less than 21 days (*n* = 599), poor parameter matching of two CT scans (*n* = 383), previous neoadjuvant drug therapy (*n* = 97), no plain CT imaging (*n* = 34), previous tumor history (*n* = 23), preoperative biopsy (*n* = 19), history of ipsilateral lung surgery (*n* = 356), interval between surgery and the last CT scan greater than 3 months (*n* = 25), incomplete clinical data (*n* = 7), and other reasons (*n* = 194, detailed in figure caption). A total of 585 patients (143 STAS-positive and 442 STAS-negative) were included in the final CHEST Cohort and 175 patients from June 2020 to December 2020 were removed from training set for in-center validation (IVC). Underlying clinical information, including age, gender, stages, type of surgery, pathological subtypes of adenocarcinoma, immunohistochemistry, and molecular pathology were obtained from medical records (Fig. 2B). Data from Zhongshan Hospital and Shanghai Ninth People’s Hospital were collected across centers in this study, and 36 (ZS Cohort in Zhongshan Hospital, with 12 STAS-positive patients) and 53 patients (Ninth Cohort in Ninth People’s Hospital, with 12 STAS positive patients) were included, respectively. The enrollment of patients and post-processing methods of the scans were in consistency with the approaches described above for CHEST Cohort. For concrete enrollment and baseline information of the external cohorts, please refer to the Additional file 1: Appendix B. The original dataset did not have a balanced number ratio on STAS ( +) and STAS (-) and differed in some of the baseline information. We performed propensity score matching (PSM) on the original dataset based on six shape or first-order features to obtain a balanced dataset of 120 patients each with and without STAS, which was likewise further divided into training and validation sets to examine the power of high-dimensional features on differential diagnosis and the performance of the model on a balanced dataset. The sample size evaluation was given in Additional file 1: Appendix O.

### Histopathological analysis

Two pathologists with years of experience in thoracic pathology blinded to clinical findings reviewed formalin-fixed paraffin-embedded sections from all included patients using microscopy independently. According to the WHO, STAS was defined as micropapillary clusters, solid nests, or single cells beyond the edge of the tumor extending into the air spaces in the surrounding lung parenchyma, which consisted of three main forms: (1) airspaces filled by micropapillary structure without central fibrovascular cores; (2) airspaces filled by solid tumor components; (3) airspaces filled by multiple discrete and discontinuous single cells. When there was disagreement, the final diagnosis was decided by discussion between the two pathologists.

### CT radiomics feature extraction

Two radiologists with 2 years’ and 16 years’ experience in chest radiology viewed the CT images to determine the C/T ratio and the CT signs. The scanned images were compressed and packaged in anonymized DICOM format and then the tumors were segmented on each layer using 3D Slicer 5.0.3. A senior radiologist with 16 years of working experience (Participant A), a thoracic surgeon of mid-level seniority with 12 years of working experiences (Participant B), a radiologist underdoing residency training (Participant C), and a thoracic surgeon undergoing residency training (Participant D) were invited to manually delineate the boundaries of the nodule on the CT image layer by layer using 3D slicer, and the 3D tumor volume could be automatically reconstructed according to their delineation. The radiomics features were extracted by PyRadiomics v3.0 open-source software program. A total of 851 features were extracted, including 14 shape features, 18 first-order statistical features, 24 Gy level co-occurrence matrix (GLCM) features, 14 Gy level dependence matrix (GLDM) features, 16 Gy level run length matrix (GLRLM) features, 16 Gy level size zone matrix (GLSZM) features, 5 neighboring gray tone difference matrix (NGTDM) features and 744 wavelet filtered features as described above. 744 wavelet filtered features were also extracted (using 8 sets of filters: LLL, LLH, LHL, HLL, LHH, HLH, HHL, HHH). The concrete content of radiomics features is introduced in Additional file 1: Appendix C. Large blood vessels and bronchi were excluded from the volume of the nodules as much as possible. The area within 3 mm of the tumor region was defined as the peritumor region, and similar feature extraction was performed for the peritumor region. The features extracted were normalized with min–max normalization method. For the scan parameters and settings of three centers, please refer to Additional file 1: Appendix B2.

### Acquisition of the 3D view for registration

The above four participants were provided with anonymized DICOM files. They were asked to select the largest cross-section of the studied tumors in the *x*-direction (i.e. the left–right direction of the body), the *y*-direction (i.e. the anterior–posterior direction of the body), and in the *z*-direction (corresponding to the superior-inferior direction of the body), and to acquire the smallest square image including their boundaries for further registration (see Additional file 1: Appendix D-F for the concrete procedures for deep learning-based feature extraction, the consistency of lesion segmentation, and the relative working arrangement). In the pre-experiment, we found that for some tumors that grow too fast in size or have strong anisotropy in growth, the registration may be unsuccessful. Therefore, four participants were simultaneously asked to delineate the morphology of the spine section at the same section with the lung tumor and frame the tumor with a minimum square if they found that the isotropy or degree of tumor growth was too obvious for registration methods to detect the feature points. In the subsequent registration procedure, these registrations will be performed according to the morphology of the spine, and the transformations will be obtained and then applied to the lung nodule images.

### Calculation of the delta-radiomics features

The definition of the delta-radiomics feature used in this work was based on the slope of time:

$${Index}_{\mathrm{delta}}=\frac{{I}_{\mathrm{followup}}-{I}_{\mathrm{baseline}}}{{t}_{\mathrm{followup}}-{t}_{\mathrm{baseline}}}$$

where ${I}_{\mathrm{followup}}-{I}_{\mathrm{baseline}}$ denotes the difference between the radiomics metrics for the baseline and the follow-up tumor volumes, and ${t}_{\mathrm{followup}}-{t}_{\mathrm{baseline}}$ is the time difference between the follow-up time and the baseline time. The metrics of delta-radiomics are essentially the slopes of the individual baseline metrics.

### Extraction of the deep-network-based features

**Fig. 3:**
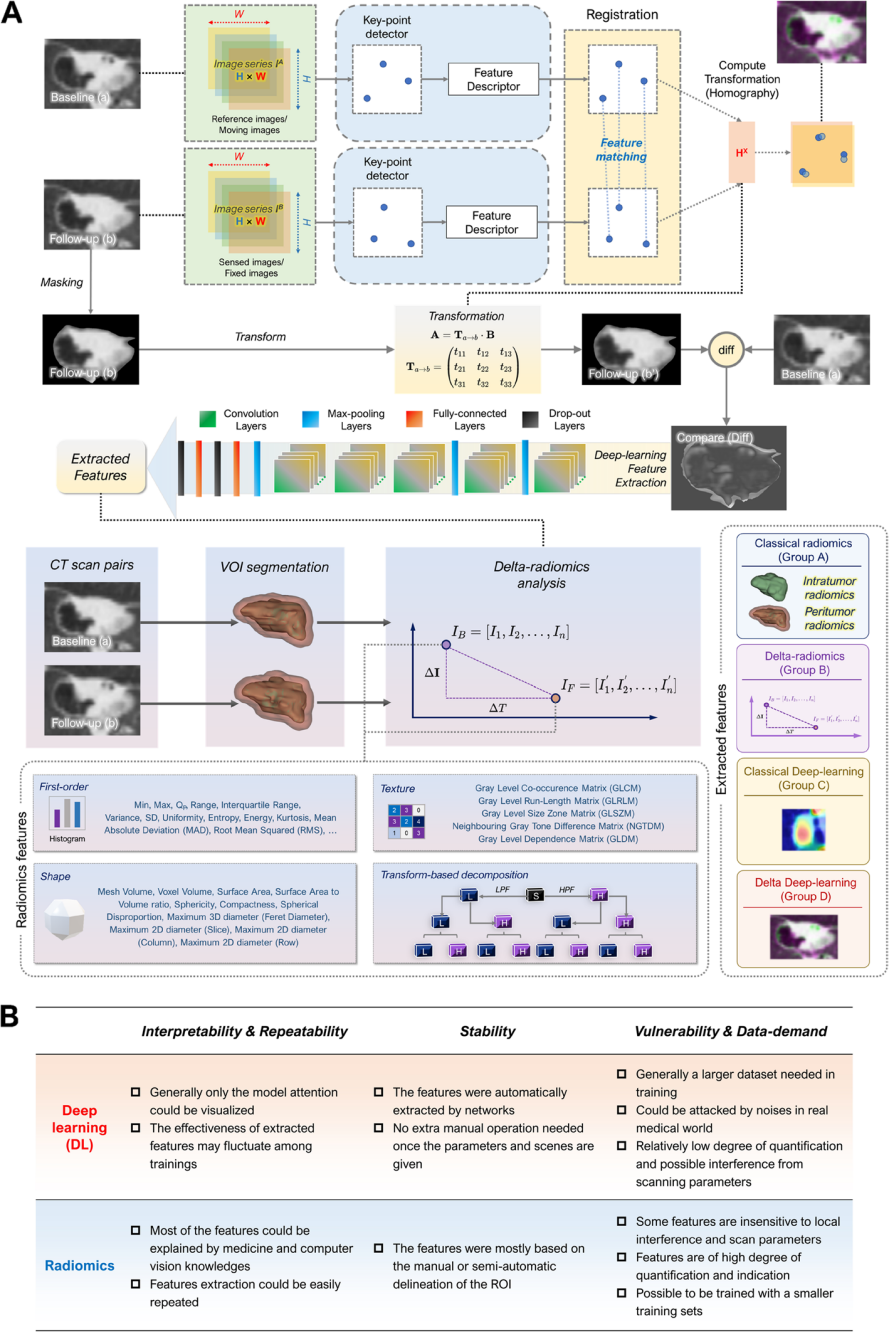
The feature extraction processes in this study and the characteristics of the extracted features. **A** The extraction of dual-delta hybrid features. **B** The characteristics (pros and cons) of deep-learning-extracted features and radiomics features compared from three aspects: interpretability and repeatability, stability, vulnerability and data-demand

A combination of graph cut and ROI manual delineation was applied to complete the initial establishment of ROI regions, followed by SNAKES (active contour algorithm) to refine the regions, and four morphological operations (erode, dilate, open, and close) to eliminate or fill specific areas (detailed in Additional file 1: Appendix D). A mask matrix could be obtained by the ROI masking, and it would be multiplied by the elements of the original image to obtain a masked ROI for subsequent registration. Various classic registration algorithms were used to detect features (detailed in Additional file 1: Appendix D). The transform matrix was then extracted by registration and applied to the baseline CT images to eliminate the adverse effect of body position, pose, etc. on further image subtraction, and then the transformed image was subtracted by matrix elements with the images from follow-up CT to obtain the delta-image, which was further fed into the deep CNN to extract the deep-learning feature set. We chose the AlexNet with balanced performance as the original CNN model, and modified it to extract features in the fully connected layer, where the neuron number (i.e., the number of delta-DL features) was 10 for each view (detailed in Additional file 1: Appendix G). The masking, registration, and CNN training were performed with MATLAB R2021b (Mathworks Inc.). An overall procedure of the feature extraction process and the characteristics of these two different kinds of features were given in Fig. 3.

### Feature selection and classifier

As described above, a total of 3404 radiomics and delta-radiomics features were extracted via intra-tumoral and peri-tumoral 3 mm volume of interest (VOI) delineations. Considering the reproducibility of the study, we chose intra/inter-class correlation coefficient (ICC) values as a criterion to measure whether these features could be consistently extracted by the same observer according to intra-group ICC values, and evaluated the consistency of these features across observers by inter-group ICC values. We select features with ICC values greater than 0.75 to enter the feature selection process. A total of 45 feature selection algorithms were taken into account to select the features (detailed in Additional file 1: Appendix H). These 45 algorithms and 32 classifiers (introduced in Additional file 1: Appendix I) formed 1,440 combinations. We selected the best combination with the highest average AUC as the final optimized model by trial-and-error method.

### Model evaluation

The ROC (Receiver Operating Characteristic)-AUC values were calculated for the evaluation of the model performances, and the Grad-CAM (Class Activation Map) were applied to visualize the attention of the deep-learning model (detailed in Additional file 1: Appendix J).

### Statistical analysis

Normally distributed variable (Age) was recorded as mean ± standard deviation (SD) or median [quartiles]. Categorical variables (variables except the age) were recorded as frequencies (proportions). The differences in the baseline characteristics between STAS ( +) and STAS (-) patients were compared using the independent t-test for continuous variable (Age), and Fisher’s exact test or Chi-square test (with or without Yeats correction) for categorical variables (variables except the age), as appropriate. A two-sided *p* < 0.05 was considered statistically significant. Statistical analyses were performed with IBM SPSS Statistics 23 (IBM, Armonk, NY, USA) and R software (version 4.0.1, the R Foundation for Statistical Computing, Vienna, Austria). The means and confidence intervals (CI) of model performance metrices (e.g. AUC, accuracy, etc.) were given after 40 independent replications of the experiments (results given in Additional file 1: Appendix K).

### Role of the funding source

The funder of the study had no role in study design, data collection, data analysis, data interpretation, or writing of the report. All authors confirm that they had full access to all the analyzed data in the study and accept the responsibility to submit for publication.

## Results

### Baseline information

The clinical information (Fig. 2B) of the patients included in this study was largely similar to the current studies in this field. Both age and gender distribution of patients were similar to previous studies [11, 13]. The percentage of female patients was 61.4% compared to 55.8% [11] and 52.3% [14] in the existing studies. The interquartile distribution of the age of patients was 60 [56,69] years compared to 60 [35,82] years in the study by Chen et al. [11], while the median age of STAS negative and positive patients included in Liao et al. was 62 [53,79] and 62 [53,87], respectively [14]. The percentage of patients in this study who were STAS positive was 24.4%, which is essentially similar to the previous researches (29.6% [11] and 33.2% [14]). The most predominant LUAD pathological subtypes in our study were the alveolar, papillary and lepidic types with proportions of 42.0%, 18.4% and 15.5%, respectively, which were similar in the study of Chen et al. with the predominant pathological subtypes being the alveolar (38.2%), lepidic (36.1%) and papillary (14.2%) types [11]. In terms of tumor staging, the percentage of patients with T_1_, T_2_, and T_3_ staging in this study was 86.7%, 7.5%, and 2.5%, respectively, compared to 75.5% and 24.5% of patients with T_1_ and T_2_ staging in the study by Chen et al. These differences may be related to the fact that only patients with two follow-up CTs were included in this study, whereas patients with multiple follow-ups were generally staged earlier and progressed more slowly. A significant improvement in the baseline information balance after PSM was achieved, as we have discussed in detail in Additional file 1: Appendix L.

### Registration results

**Fig. 4:**
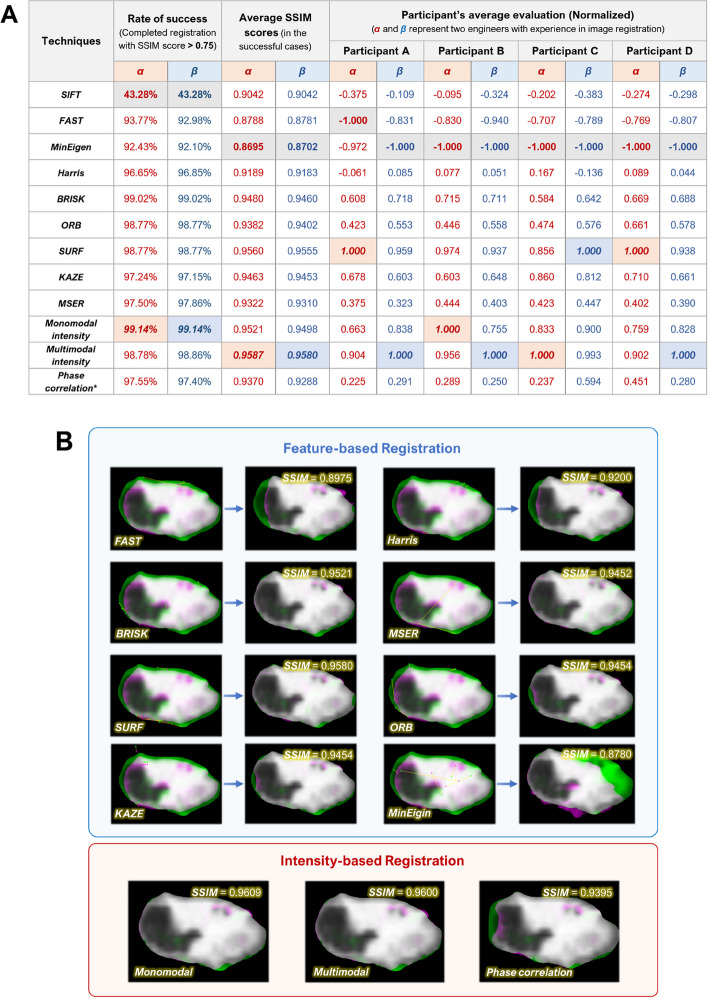
Registration results. **A** SSIM scores of various registration methods. **B** SSIM scores and examples for three different types of registration methods (shown in Green-Magenta pattern)

Figure 4A shows the performances of various registration methods. The multimodal registration methods yielded the highest SSIM values with the value of 0.9587 (Engineer *α*) and 0.9580 (Engineer* β*). Figure 4B demonstrates the some SSIM scores and examples of the three different types of registration methods.

### Diagnostic capabilities of models in the real world

**Fig. 5:**
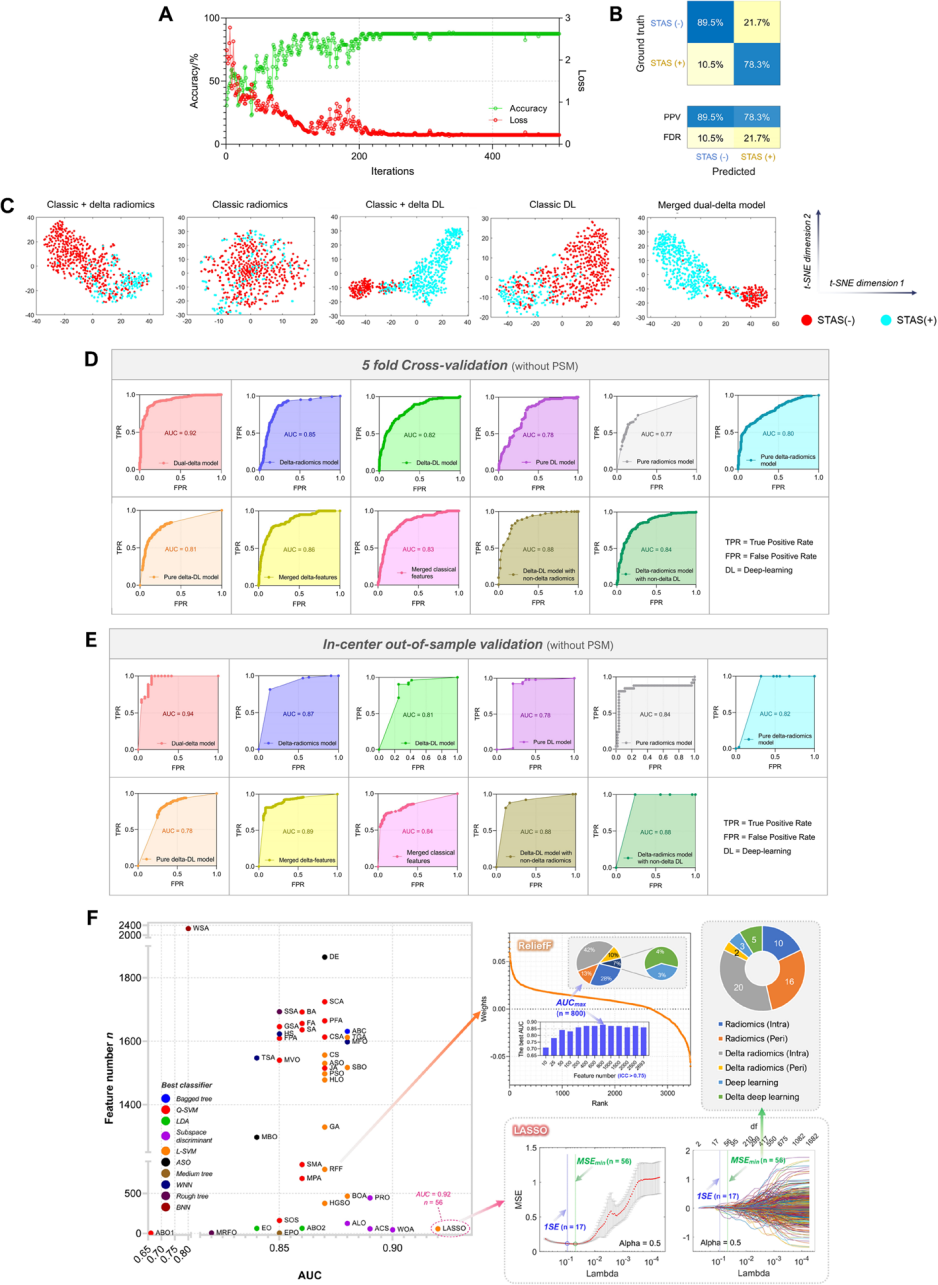
Results of five-fold cross-validation and in-center validation in real world. **A** Training curve of AlexNet model extracting the delta-DL features (ICV accuracy vs. ICV loss during the training process). **B** A representative confusion matrix of a near-average classification result by dual-delta machine learning model. **C** T-SNE unsupervised clustering of features. **D** Five-fold cross-validation ROC curves and their AUC values. **E** In-center validation ROC curves and their AUC values. **F** AUC values and the feature numbers for the combinations of feature selection algorithms and their optimal classification models, where the LASSO cross-validation plot and LASSO trajectory plots of variables (green vertical lines represent the number of features corresponding to MSE_min_), and the ranked feature weights by ReliefF (pie charts show the compositions of essential feature sets selected by LASSO and ReliefF) are given

As mentioned previously, the data were allocated in two different ways, corresponding to the real-world situation (without PSM) and to the situation when the shape and first-order features of tumors are essentially similar (with PSM where these features were selected for matching). The power of the model for differential diagnosis in the real world was first assessed (Fig. 5). Figure 5A is the training curve of AlexNet for extracting Delta deep-learning (delta-DL) features, which showed that the model was able to converge. Figure 5B shows the confusion matrix that was closest to the average categorical performance at a particular time in the repeated trials, and the accuracy of the model can be calculated as 87.0%, sensitivity as 70.6%, and specificity as 92.3%. Figure 5C provides the t-SNE unsupervised clustering results for features extracted before feeding into the final classifier. It can be seen that, starting from classic DL or radiomics, the clustering results of the model for STAS( +) and STAS(-) get closer to the complete separation state with the introduction of delta information, which makes the two point sets more separable. This observation was in line with the five-fold cross validation (Fig. 5D) and in-center out-of-sample validation results (Fig. 5E) where the AUC values (AUC_5-fold_ = 0.92, AUC_out-of-sample_ = 0.94) of the proposed dual-delta model outperformed the component models. Our results suggest that the combination of LASSO regression + linear SVM model is relatively optimal and can accomplish the classification task with an AUC value of 0.92 (Fig. 5F). The cross-validation curves and coefficient trajectory curves of the LASSO regression are further given, and it can be seen that the LASSO regression extracts a total of 56 significant features corresponding to the MSE_min_, and among them 22 features (39.3%) were from the delta-radiomics and 5 features (8.9%) were from delta-DL. We also extracted the features filtered by ICC and selected the top *n* features by weight ranking with ReliefF to enter into the classification model for analysis. Our result showed that the top 800 features (42% delta-radiomics features, 4% delta-DL features) corresponded to the optimal classification performance (selected by AUC), but the AUC value only reached 0.87.

### Differential diagnostic ability of the model in similar nodules

The differential diagnostic power of proposed model was further examined with the post-PSM data, which were balanced in most baseline indexes since the two groups in the new dataset were matched according to six essential shape and histogram features. In Additional file 1: Appendix L, Supplementary Figure L2 shows the feature selection and classifier optimization results, which suggests that the combination of LASSO regression + linear SVM model was optimal, with an AUC value of 0.91, outperforming each component model in both 10-fold cross validation (Supplementary Figure L2-A) and in-center out-of-sample validation (Supplementary Figure L2-B). A total of 58 features were included in the final optimized model, where the delta-DL features (5/58) and delta-radiomics features (21 intratumor features and 2 peritumor features) occupied a significant proportion (Supplementary Figure L2-C).

### Model performance in different follow-up time scenarios and the cross-center applicability

**Fig. 6:**
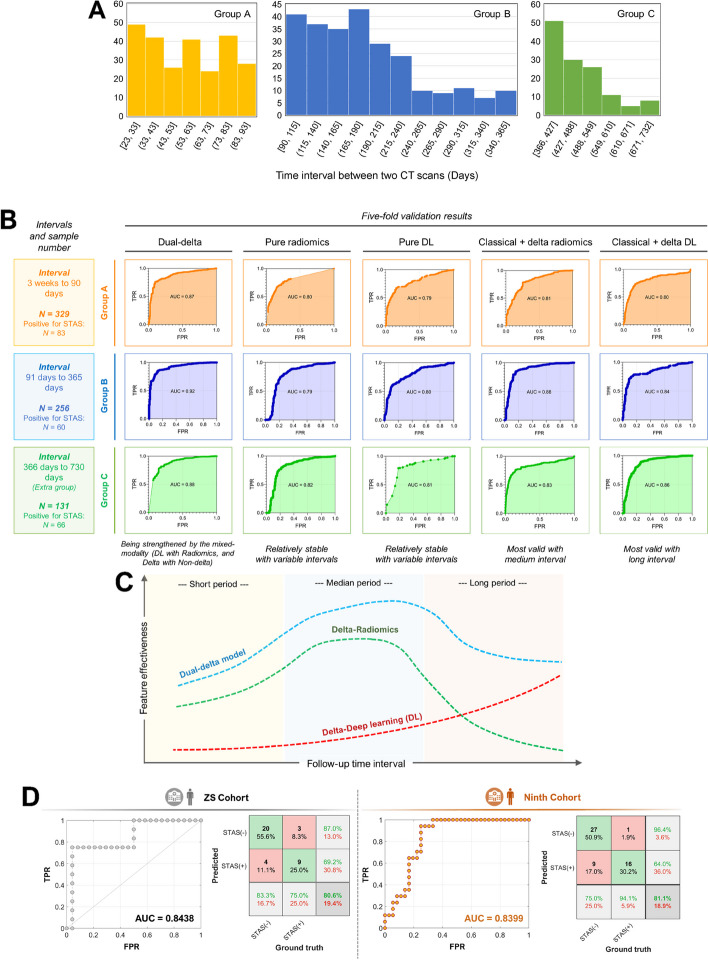
Effect of different follow-up intervals on model performance and the cross-center performance of the model. **A** Frequency distribution of different follow-up time groups. **B** Performances of different follow-up time groups by different models. **C** A schematic diagram showing possible relationships between the feature effectiveness and follow-up time interval in different models. **D** External validation results showing the ROC curves and confusion matrices of Zhongshan cohort and Ninth Hospital cohort

We further collected CT scans from 131 patients who were excluded in forementioned enrollment procedure due to their in-center follow-up intervals of more than a year but less than two years. Subsequently, these newly-enrolled 131 patients and 585 patients in the previous CHEST Cohort were combined together and classified into three groups according to the length of follow-up interval. Patients in group A, B, and C had a follow-up interval between three weeks and three months (*n* = 329), between three months and a year (*n* = 256), and between one and two years (*n* = 131), respectively (Fig. 6A). A five-fold cross-validation was used to obtain AUC values to determine the ability of the different models to predict the tumor STAS status of patients in groups A, B, and C (Fig. 6B).

The dual-delta model still had the most satisfactory classification performance in each subgroup. The classic radiomics model and the classic DL model had performed consistently in each subgroup. The absence of time concept in the classic models made them insensitive to different follow-up intervals since they only used the information extracted from the last CT for the model training. However, for the models incorporating delta information, differences in AUC values among three follow-up time groups were observed. For the delta-radiomics model, the classification accuracy of group B was significantly higher than those of the other subgroups, which could be interpreted by the insignificant (compared to possible noises and other irrelevant inferences) difference in radiomics extracted from the tumor due to the limited time intervals. Therefore, the effective signal-to-noise ratio for precise classification was not large enough. However, the growth of the lung tumor could also show stagnation, slowdown, and other complicated behaviors in a long period, which made the changes in radiomics features in the long time-intervals appeared insignificant when calculating the time-based slope values. In contrast, the delta-DL model has the best prediction for group C with long follow-up intervals. After the image registration and subtraction, the scan pairs with long interval have the most abundant graphical information since their corresponding growth areas of the tumor were generally bigger than those with shorter follow-up intervals, and information in these remaining areas may provide more “learning materials” to the DL model for its response and activation. Two different models describe the tumor growth process from different perspectives, where the radiomics is a quantitative computer vision metric description, and the deep-learning method describes the images with a graphical-based feature map. Their combination makes the models stable to different follow-up time intervals and robust to possible interference information and radiological noises (Fig. 6C).

For cross-center validation results (Fig. 6D), the AUC value for the ZS Cohort was 0.844, with an accuracy of 80.6%, a sensitivity of 75.0%, and a specificity of 83.3%; the AUC value for the Ninth Cohort was 0.840, with an accuracy of 81.1%, a sensitivity of 94.1%, and a specificity of 75.0%.

### Analysis of model attention and interpretability

**Fig. 7:**
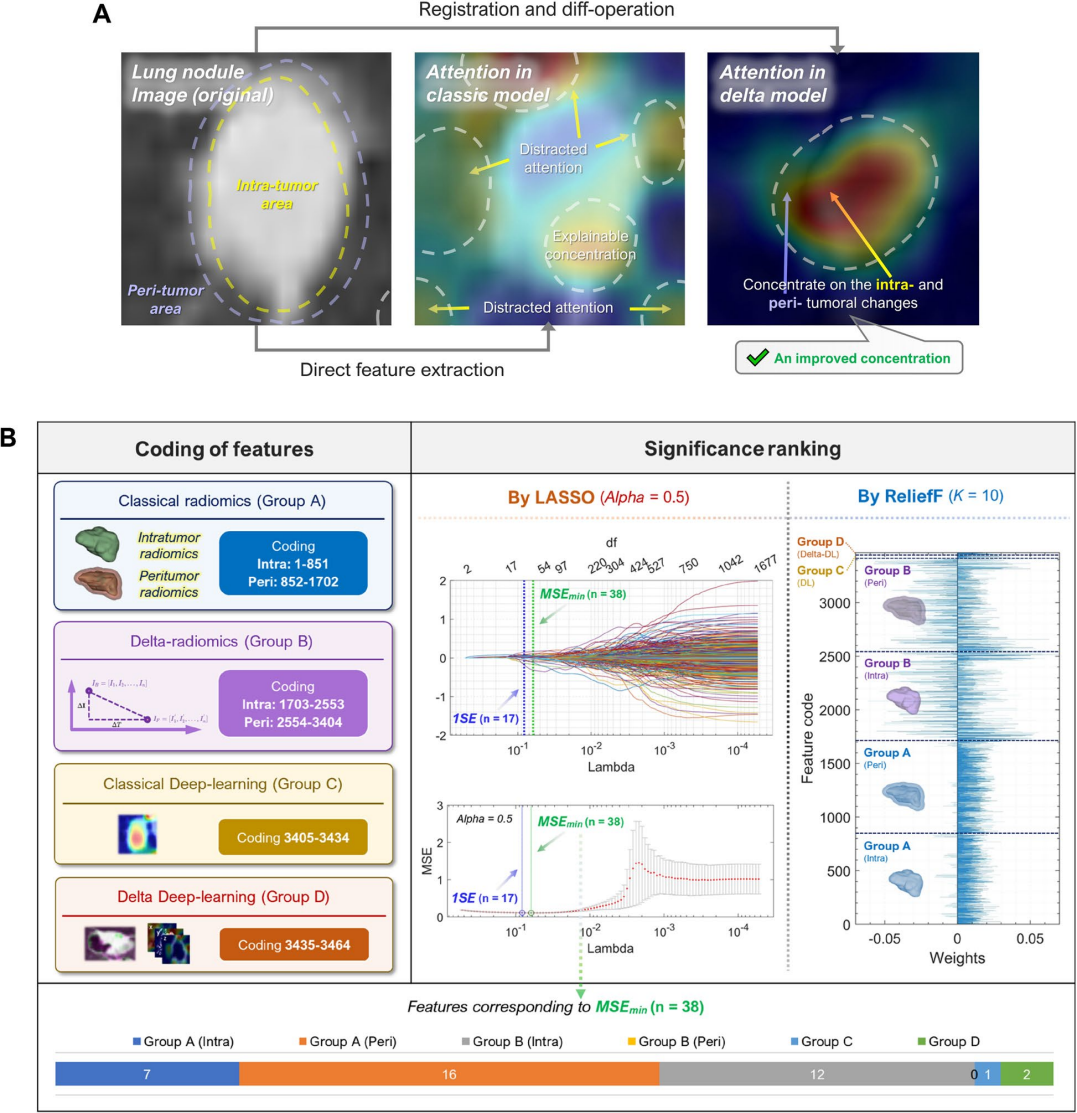
The model interpretability. **A** A representative example of GRAD-CAM visualization result for CNN classification, where the attention distributions of classic DL model and delta-DL model were shown in annotated images. **B** The essential feature set selected by LASSO and ReliefF and their compositions

Figure 7A shows the Grad-CAM visualization results of the classic DL model and the delta-DL model, and five example graphics are randomly selected among the correct classification results in Additional file 1: Appendix M. For the classic DL model, the attention of CNN model was more scattered. Although most of the attention was focused on the tumor and peri-tumor region, there was a scattering of attention to the background, while for the delta-DL model, the CNN attention was obviously more focused than the former, and the areas that make important contributions to the classification were concentrated in the region reflecting the tumor growth, which suggests that the delta-DL model essentially helped the deep network to “learn absorbedly” by focusing its attention on regions that contribute more to the facts, which improved the intuitiveness and humaneness of the learning.

To further investigate the role of radiomics and DL features in the classification task, we extracted radiomics and deep-learning features from VOIs and 3D tumor views delineated by participant A who had the most experience in medical image processing and chest radiology among four participants. These features corresponding to all 585 patients in CHEST cohort were used for model training without ICC-based feature screening. The results of feature selection by the LASSO (alpha = 0.5) and feature ranking by ReliefF algorithm (*K* = 10) were considered, and the contents and coding of the features were given in Fig. 7B. The results of LASSO regression showed a total of 38 features correspond to the minimum mean squared error (MSE_min_). According to the feature weights given by ReliefF, the classic radiomics information was relatively robust in classification with only few features with negative weights. However, compared to the delta-radiomics features, the classic radiomics features had less contribution to the significant positive feature set. The delta-radiomics features and delta-DL features had larger absolute values of weights than those of classic radiomics, suggesting that they may include information that contribute significantly to the classification results (more detailed supporting information in Additional file 1: Supplementary Figure N1 in Appendix N). Meanwhile, it also demonstrated that the subsequent feature selection procedures not only reduced the model dimensionality and improved the efficiency of training and computing, but also had great significance for ensuring the classification accuracy of the model. Among the 38 features in the essential set selected by LASSO, most of them (37/38, 97.4%) were with intra-group and inter-group ICC values over 0.75, testifying a satisfactory reliability and repeatability of the core feature set. A more detailed analysis of model interpretability could be found in Additional file 1: Appendix N.

## Discussion

STAS is an independent prognostic factor for poor recurrence-free survival and overall survival, which can greatly influence surgical decisions [3–5]. However, there is no reliable preoperative method to predict STAS [6, 7]. Radiomics pipeline converts radiological scans into quantifiable information, facilitating the model building and interpretation. Several previous studies have shown that radiomics is a promising method in predicting STAS status in lung cancer patients, and several studies have obtained good predictive performance (AUC 0.66–0.90; accuracy 68%-93%; sensitivity 74%-88%) [10–14, 19–22]. Our dual-delta model with pure radiological information couples the DL model based on images generated by the subtraction between the follow-up scan images and post-registration baseline images with the delta-radiomics model. To the best of our knowledge, it is the first study using CT-based delta-radiomics combined with deep-learning to predict STAS status in primary lung cancer. The main findings demonstrate that the model has good efficacy in predicting STAS in lung cancer (five-fold cross-validation AUC = 0.92, intra-center validation AUC = 0.94, external ZS cohort AUC = 0.84, and external NINTH cohort AUC = 0.84). Delta-radiomics with deep network model is therefore expected to be a preoperative biomarker discovery strategy for predicting STAS status and provide reliable support for clinical surgery options and other treatment plans. Meanwhile, the radiological scan-only information avoided the potential error from the inaccurate/incomplete clinical variables and human-determined CT signs.

Our study also demonstrates the value of peritumoral features and their corresponding delta features in the model construction. STAS is characterized by the spread of tumor cells in different forms within the air spaces of the surrounding lung parenchyma, which usually extends beyond the primary tumor margin. Actually, many CT signs of STAS in lung cancer reported by Toyokawa et al. were associated with peritumoral components, such as vascular convergence, peripheral gross glassy opacity (GGO), air bronchogram, and pleural indentation [8]. Moreover, it has been shown that peritumor-based features outperform traditional tumor-based imaging in predictive STAS [13, 14]. Therefore, we additionally extracted peritumor features within 3 mm of the tumor margin and combined them with tumor features, which enhanced the predictive performance of our model.

The present study still has several limitations. First, this study is still a retrospective analysis of CT findings based on pathological findings and not a prospective study, so future prospective cohort studies may be needed to further confirm our findings. The pathological types of investigated tumors were still not abundant, and some of the pathologic types such as squamous and small cell lung cancer were not sufficient, although the sub-group analysis on the pathological types of tumors did not reveal this insufficiency or bias caused the imbalanced model performances in different pathological groups (details could be found in Additional file 1: Appendix P). Second, due to the nature of retrospective studies, the patient population included in the experimental design (585) remains limited compared to the overall population (2812) with no less than two CT follow-up scans. According to a previous study on STAS in the same center, the selection bias was likely to exist [23]. A larger external training or validation cohort from more centers and larger cohort is still needed to improve or evaluate the predictive effect of the proposed model. Third, the patients included in this study were treated with surgical resection, which may have overlooked some cases of small tumors with stage III and IV cases and consequently introduced selection bias. Fourth, systematic or subjective errors still exist with respect to the pathological diagnosis of STAS. Although the concept of STAS has been established in the thoracic pathology for many years, there is still no universally accepted pathological diagnostic standard, especially due to the presence of ex vivo artifacts. It has been found that STAS may sometimes be simply an artifact caused by tumor destruction, with tumor cells spreading along the alveolar cavity through the lung specimen section in vitro, an artifact very graphically referred to as “spreading through the knife surface”, which makes the labeling determination potentially inaccurate. Although recent studies demonstrated that STAS is an in-vivo phenomenon instead of a pathologist-related artifactual event because of knife transportation of tumor cells during gross specimen handling [24, 25], there is still a critical need to minimize the artifactual clusters and to further refine and standardize the histopathologic definitions of STAS [6, 26]. Fifth, the delineations of VOIs were still subjective and time-consuming. The semi-automatic segmentation techniques supported by software such as 3D Slicer (e.g., level tracing and grow from seeds) cannot yet completely avoid the subjectivity, which leads to the inevitable interference of lymph nodes and blood vessels in the VOI segmentation for a portion of lesions such as large masses near hilar. The feature screening based on ICC was adopted to evaluate and eliminate the influence of the subjective factors as much as possible. Intra-group repeated delineations and inter-group comparative tests were designed to ensure the reliability and repeatability of the final features included for further selection based on algorithms. Sixth, our study places demand on the quality control of CT image sequences, especially with respect to respiratory movements. The chest/respiratory motion may generate adverse effects on the lesion registration from many aspects. The respiratory motions have been shown to affect the volume and density measurement of lung nodules, and the motion artifacts cause poorer imaging quality, such as blurred nodal boundaries that can cause the failure of alignment algorithms based on edge information, while the displacement of high-intensity signals in the middle of the image and the blurring of the boundaries also make intensity-based feature extraction prone to failure or error. Furthermore, the alterations in the relative relationship between the spine and the lung tumor positions can also occur with the respiratory motion, which will adversely affect the registration of lung tumors, especially tumors with significant morphological changes, as it may require different planes of the tumor for alignment (priority must be given to ensuring that the same level of the spine is used for alignment, as they are usually absolutely rigid).

The implications of this study remain significant. First, to the best of our knowledge, it is a pioneer study to introduce the delta-radiomics model to predict STAS status in primary lung cancer. Second, this study not only discussed the significance of delta-radiomics for STAS prediction for the first time, but also added an emerging modality, the deep-learning features based on image registration and subtraction in the context of clinical follow-up to give dynamic description of lung nodule growth, which was a modality that effectively complements delta-radiomics, making the combination model more intuitive than classic radiomics pipeline. Meanwhile, it provides features based on deep-learning-based computer vision, which improves the accuracy of prediction and guards the model against the effect of different follow-up time intervals on the prediction effect. Third, the prediction results of the model were satisfactory and have been validated in multiple centers, indicating that the model has generalized cross-center performance, good reproducibility and robustness, which provides a reliable basis for clinical decision-making. Fourth, the methodological value of this study is not only to demonstrate the potential of delta-radiomics in predicting tumor pathological features, but also to suggest that the combination of quantitative description based on radiomics and CNN-based machine vision could effectively improve the model performance, which provides new thinking for clinical scenarios such as tumor diagnosis and treatment monitoring in the future medicine, especially in the follow-up of solid lesions.

## Conclusion

The current study explored the potential value hidden in the dynamic radiological description of lung tumors by delta-radiomics and DL-based features extracted by CNNs from subtracted images pre-processed with image registration. To the best of our knowledge, no report has investigated the potential benefits of combining delta-radiomics and registration-based DL from subtracted images to improve prediction of STAS. Our dual-delta hybrid model combining four elements (delta-radiomics, delta-DL, classic radiomics, and classic DL) outperformed the component model by discrimination and showed reliable performance in multicenter cohorts and patients with various follow-up intervals. Additionally, the interpretability of the proposed model further assessed with Grad-CAM for DL-features and feature ranking for radiomics features. Meanwhile, as a down-sampling method, PSM was adopted to further provide a balanced dataset for model training and validation. Our model showed satisfactory and stable performances on both the real-world and post-PSM datasets.

Our study suggests that the combination of delta-DL model based on the registration methods and image subtraction with the delta-radiomics model enriches information such as anisotropy of tumor growth and intratumor heterogeneous changes during the CT follow-up, which could provide valuable information for the prediction of STAS in primary lung cancer.

### Supplementary Information

**Additional file 1: Appendix A.** Related AI-assisted model for STAS prediction. **Appendix B.** Baseline information and CT scan parameters of CHEST cohort and two external cohorts. **Appendix B1.** The enrollment criteria of the two external centers. **Appendix B2.** The baseline information of the involved centers. **Appendix B3.** The scan information/parameters of the involved centers. **Appendix C.** A detailed introduction to the radiomics and delta-radiomics features. **Appendix D.** Concrete procedures for deep learning-based feature extraction. **Appendix E.** The consistency of lesion segmentation. **Appendix F.** Working arrangement. **Appendix G.** Architecture of the deep network. **Appendix H.** Feature selection. **Appendix I.** Classifiers considered in this study. **Appendix J.** Model evaluation methods. **Appendix K.** Model repetitiveness (*N* = 40). **Appendix L.** The results of PSM and their analysis. **Appendix M.** Visualization of model attention with Grad-CAM. **Appendix N.** On the interpretability of the features. **Appendix O.** On the sample size evaluation. **Appendix P.** On the bias of pathological types.

## Data Availability

To protect the patient privacy, the data related to patients cannot be available for public access. However, they can be obtained from the corresponding authors on reasonable request approved by the institutional review board of all enrolled centers.

## Abbreviations

CT: Computed tomography
STAS: Spreading through air spaces
DL: Deep learning
MSE: Mean squared error
Grad-CAM: Gradient-weighted class activation mapping
VOI: Volume of interest
CNN: Convolutional Neural Network
PSM: Propensity score matching
RF: Random forest
CV: Computer vision
NSCLC: Non-small cell lung cancer
ROC: Receiver operating characteristic
GGN: Ground-glass nodule
SD: Standard deviation
LASSO: Least absolute shrinkage and selection operator
AUC: Area under curve
