# Supplementary material for: Improving the prediction of Spreading Through Air Spaces (STAS) in primary lung cancer with a dynamic dual-delta hybrid machine learning model: a multicenter cohort study
Source: Biomark Res. 2023 Nov 23;11:102. doi: 10.1186/s40364-023-00539-9 (PMC10668492; doi:10.1186/s40364-023-00539-9)
Supplement: Supplementary file 1 — Additional file 1: Appendix A. Related AI-assisted model for STAS prediction. Appendix B. Baseline information and CT scan parameters of CHEST cohort and two external cohorts. Appendix B1. The enrollment criteria of the two external centers. Appendix B2. The baseline information of the involved centers. Appendix B3. The scan information/parameters of the involved centers. Appendix C. A detailed introduction to the radiomics and delta-radiomics features. Appendix D. Concrete procedures for deep learning-based feature extraction. Appendix E. The consistency of lesion segmentation. Appendix F. Working arrangement. Appendix G. Architecture of the deep network. Appendix H. Feature selection. Appendix I. Classifiers considered in this study. Appendix J. Model evaluation methods. Appendix K. Model repetitiveness (N = 40). Appendix L. The results of PSM and their analysis. Appendix M. Visualization of model attention with Grad-CAM. Appendix N. On the interpretability of the features. Appendix O. On the sample size evaluation. Appendix P. On the bias of pathological types. [file 40364_2023_539_MOESM1_ESM.pdf]

Supplement for

Improving the Prediction of Spreading Through Air Spaces (STAS) in Primary Lung Cancer with a Dynamic Dual-delta Hybrid Machine Learning Model: A Multicenter Cohort Study

Weiqiu Jin[1], Leilei Shen[2,3,4,10], Yu Tian[1], Hongda Zhu[1], Ningyuan Zou[1], Mengwei Zhang[5], Qian Chen[6], Changzi Dong[7], Qisheng Yang[8], Long Jiang[1], Jia Huang[1,*], Zheng Yuan[9,*], Xiaodan Ye[2,3,4,10,*], and Qingquan Luo[1,*]

1. Shanghai Lung Cancer Center, Shanghai Chest Hospital, Shanghai Jiao Tong University School of Medicine, Shanghai, 200030, China.
2. Department of Radiology, Zhongshan Hospital, Fudan University, Shanghai, 200032, China.
3. Shanghai Institute of Medical Imaging, Shanghai, 200032, China.
4. Department of Cancer Center, Zhongshan Hospital, Fudan University, Shanghai, 200032, China.
5. School of Medicine, Shanghai Jiao Tong University School of Medicine, Shanghai, 200025, China.
6. Department of Radiology, Ruijin Hospital, Shanghai Jiao Tong University School of Medicine, Shanghai, 200025, China.
7. Department of Bioengineering, School of Engineering and Science, University of Pennsylvania, Philadelphia, 19104, USA.
8. School of Integrated Circuits & Beijing National Research on Information Science and Technology (BNRist), Tsinghua University, Beijing, 100084, China.
9. Department of Radiology, Shanghai Ninth People's Hospital, Shanghai Jiao Tong University School of Medicine, Shanghai, 200011, China.
10. Department of Radiology, Shanghai Chest Hospital, Shanghai Jiao Tong University, Shanghai, 200030, China.

*** Correspondence to:** Jia Huang, huangjiadragon@126.com; Zheng Yuan, yuanzheng0404@163.com; Xiaodan Ye, yuanyxd@163.com; Qingquan Luo, luoqingquan@hotmail.com

A Graphical Abstract (GA) for This Work

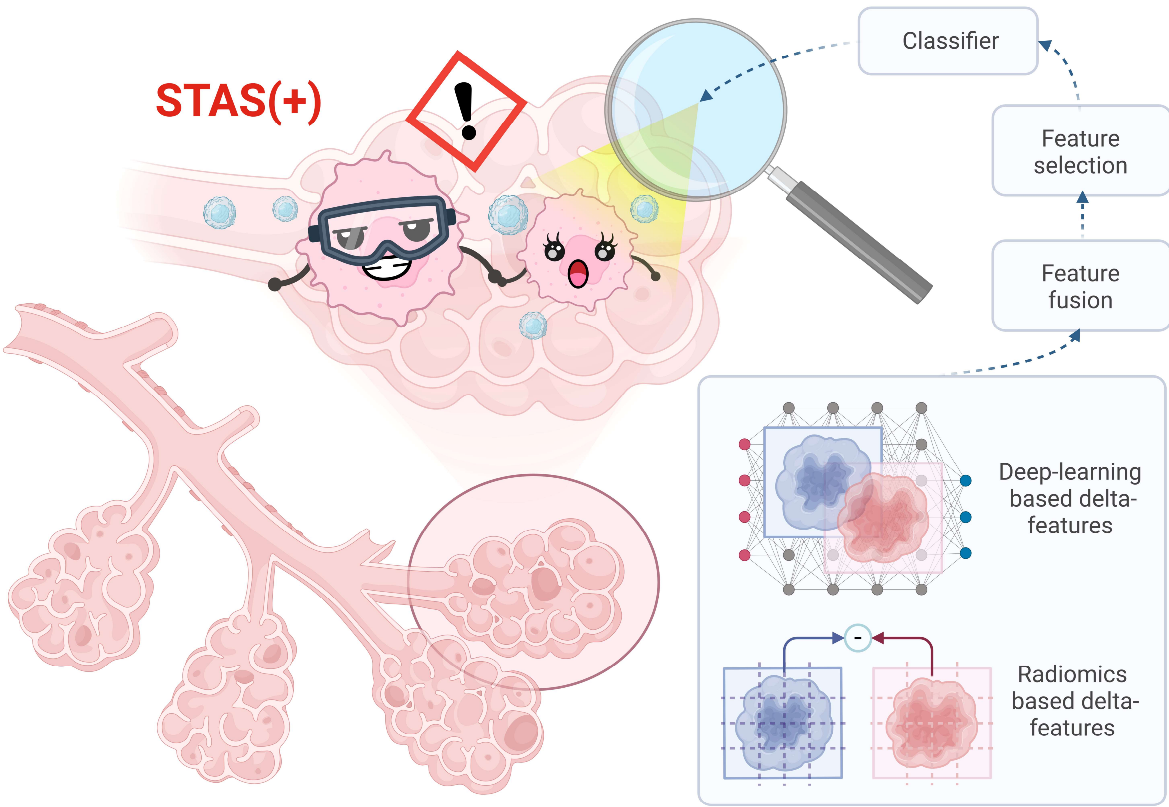

Illustration for GA:

This work demonstrates a novel model that integrates deep learning networks and Delta-radiomics to predict STAS in primary lung cancer patients. This study not only evaluates for the first time the significance of Delta-radiomics in primary lung cancer STAS prediction, but also introduces for the first time Delta-deep learning features based on image registration. The fusion of the two models not only effectively improves prediction, but also complements each other to form a comprehensive model for lung nodule growth evaluation. We found that this model is not only suitable for longer follow-up interval, but also more interpretable and intuitive than the classic deep learning model, which is one of the most powerful models for STAS prediction at present. The figure above shows some of the basic concepts of this model, where the delta deep-learning features extracted by deep learning and delta-radiomics defined by time-based slopes are fused and selected by multiple algorithms, and the prediction could be finally produced by a classifier to give STAS labels.

Appendix A | Related AI-assisted model for STAS prediction

In recent years, benefiting from the development of radiomics, computers can extract histological features from CT images to quantify image information in high throughput, which not only reduces the interference of subjective judgment and improves prediction performance, but also identifies digital information that cannot be observed and summarized by the human eyes and knowledges, improving the effectiveness of using image information. Some studies have already shown that radiomics quantifies some biological features of tumors from image information to help predict STAS more accurately. **Table 1** gives a summary of related AI-assisted model for STAS prediction, including the sample size, main AI methods, AUC values, accuracy, sensitivity, and specificity.

Supplementary Table A | Related AI-assisted model for STAS prediction

Center[#]	STAS (+)	Size	Methods	AUC (95% CI*)	Accuracy	Sensitivity	Specificity	Ref.
S	90	462	RF	0.754	/	88.00%	58.80%	[1]
S	19	92	RF	/	93.31%	/	/	[2]
S	35	226	XGBoost	0.77	/	/	/	[3]
M	65+33	99+55	LR	0.66	68.00%	77.00%	53.00%	[4]
M	69+50	233+112	Naïve Bayes	0.69	/	/	/	[5]
S	95	339	LASSO	0.76 (0.67-0.84)	/	/	/	[6]
S	169	395	LASSO + LR	0.85	79.70%	74.30%	/	[7]
S	56	216	AdaBoost	0.909 (0.863-0.949)	84.90%	/	/	[8]
S	85	256	LASSO	0.87 (0.78–0.96)	85.00%	74.00%	91.00%	[9]
S	89	203	LR	0.8 (0.65-0.86)	72.00%	/	/	[10]
M	143+29	585+89	Dual-delta + LASSO + L-SVM	0.92 (0.90-0.94)	87.00%	70.60%	92.30%	This work

LR = logistic regression, RF = random forest.

* If the CI was reported in the work.

[#] S = single center, M = multicenter.

Appendix B | Baseline information and CT scan parameters of CHEST cohort and two external cohorts

Appendix B1 *The enrollment criteria of the two external centers*

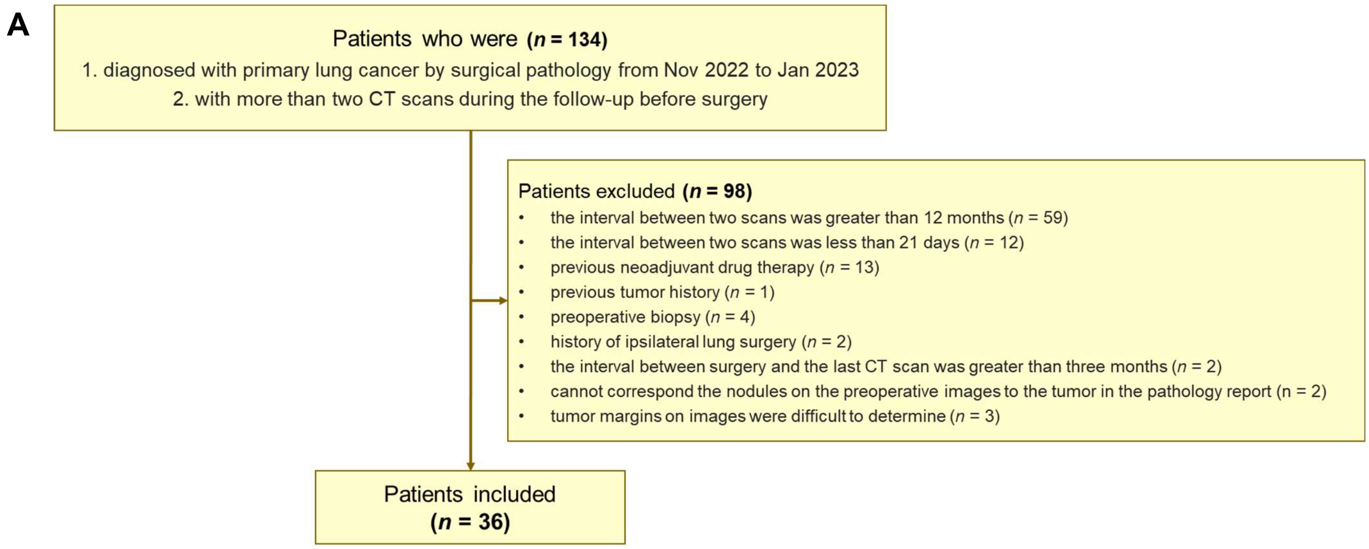

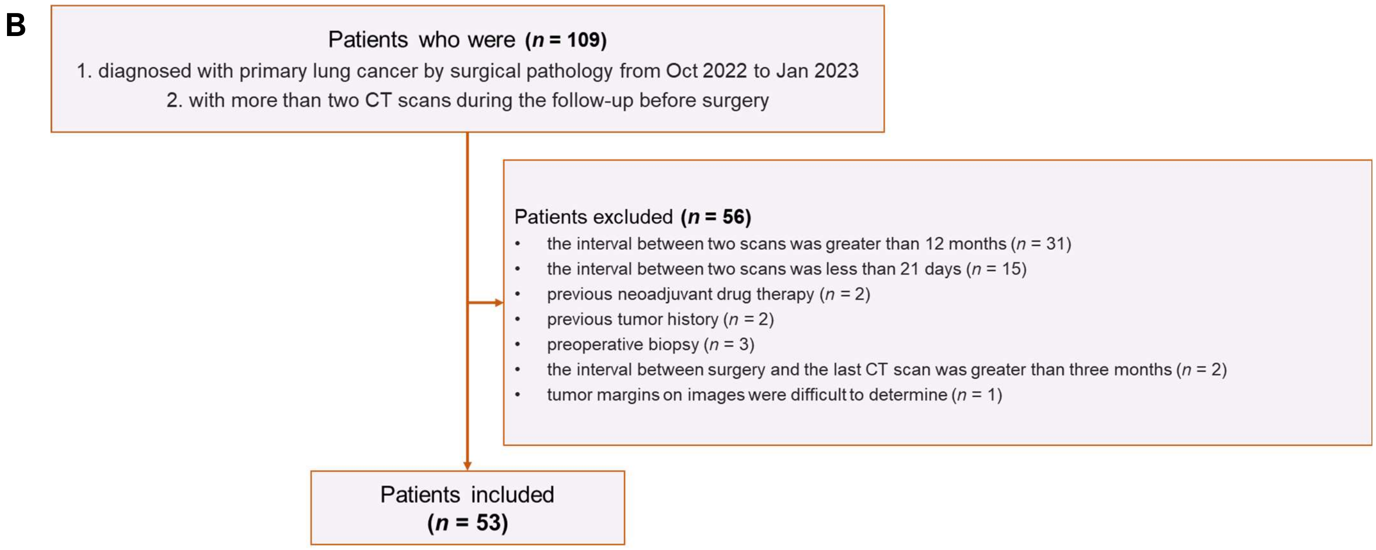

Supplementary Figure B1 | Patient enrollment and exclusion criteria in two external validation cohorts. **A.** ZS Cohort. **B.** Ninth Cohort.

Appendix B2 *The baseline information of the involved centers*

Supplementary Table B2 | Baseline, surgical, and pathological information of patients (**CHEST Cohort**)

**Variables**	**All patients** *n* = 585	**STAS (-)** *n* = 442 (75.6%)	**STAS (+)** *n* =143 (24.4%)	**STAS (+) (1:1 PSM)** *n* = 120 (50.0%)	**STAS (-) (1:1 PSM)** *n* = 120 (50.0%)	p value[A]	p value[B]
**Gender**						0.586	0.595
Male	226 (38.6%)	168 (38.0%)	58 (40.5%)	48 (40.0%)	44 (36.7%)		
Female	359 (61.4%)	274 (62.0%)	85 (59.5%)	72 (60.0%)	76 (63.3%)		

**Age (year)**						0.232	0.187
Mean ± SD	58.6 ± 11.3	58.0 ± 9.3	61.1 ± 12.0	61.5 ± 11.4	57.8 ± 10.6		
Median (IQR)	60 [56, 69]	61 [55, 67]	63 [57, 69]	65 [53, 69]	56 [47, 67]		
**Smoking**						0.157	0.925
Never	349 (59.7%)	273 (61.8%)	76 (53.1%)	67 (55.8%)	70 (58.3%)		
Former/current	231 (39.4%)	166 (37.6%)	65 (45.4%)	51 (42.5%)	48 (40.0%)		
Unknown	5 (0.9%)	3 (0.6%)	2 (1.4%)	2 (1.7%)	2 (1.7%)		
**pT stage (AJCC 8th edition)**						<0.001	0.331
Tis	23 (3.9%)	23 (5.2%)	0 (0.0%)	0 (0.0%)	1 (0.8%)		
Tia (mis)	48 (8.2%)	48 (10.9%)	0 (0.0%)	0 (0.0%)	2 (1.7%)		
T1	447 (76.4%)	315 (71.3%)	132 (92.3%)	110 (91.7%)	104 (86.7%)	0.039	0.592
T1a	220 (37.6%)	165 (37.3%)	55 (38.5%)	45 (37.5%)	39 (32.5%)		
T1b	147 (25.1%)	102 (23.1%)	45 (31.5%)	35 (29.2%)	40 (33.3%)		
T1c	80 (13.7%)	48 (10.9%)	32 (22.4%)	30 (25.0%)	25 (20.8%)		
T2	40 (6.8%)	31 (7.0%)	9 (6.3%)	8 (6.7%)	9 (7.5%)	0.404	> 0.999
T2a	38 (6.5%)	30 (6.8%)	8 (5.6%)	7 (5.8%)	8 (6.7%)		
T2b	2 (0.3%)	1 (0.2%)	1 (0.7%)	1 (0.8%)	1 (0.8%)		
T3	8 (1.4%)	6 (1.4%)	2 (1.4%)	2 (1.7%)	3 (2.5%)		
T4	0 (0.0%)	0 (0.0%)	0 (0.0%)	0 (0.0%)	0 (0.0%)		
Not applicable#	19 (3.2%)	19 (4.3%)	0 (0.0%)	0 (0.0%)	1 (0.8%)		
**pN stage (AJCC 8th edition)**						<0.001	0.537
N0	457 (78.1%)	357 (80.7%)	100 (69.9%)	86 (71.7%)	92 (75.8%)		
N1	70 (12.0%)	47 (10.6%)	23 (16.1%)	18 (15.0%)	16 (13.3%)		
N2	39 (6.7%)	19 (4.3%)	20 (14.0%)	16 (13.3%)	11 (9.2%)		
N3	0 (0.0%)	0 (0.0%)	0 (0.0%)	0 (0.0%)	0 (0.0%)		
Not applicable	19 (3.2%)	19 (4.3%)	0 (0.0%)	0 (0.0%)	1 (0.8%)		
**pM stage (AJCC 8th edition)**						0.795	0.719
M0	562 (96.1%)	422 (95.5%)	140 (97.9%)	118 (98.3%)	118 (98.3%)		
M1a	4 (0.7%)	1 (0.2%)	3 (2.1%)	2 (1.6%)	1 (0.8%)		
M1b	0 (0.0%)	0 (0.0%)	0 (0.0%)	0 (0.0%)	0 (0.0%)		
M1c	0 (0.0%)	0 (0.0%)	0 (0.0%)	0 (0.0%)	0 (0.0%)		
Not applicable	19 (3.2%)	19 (4.3%)	0 (0.0%)	0 (0.0%)	1 (0.8%)		
**Surgery**						<0.001	0.001
Lobectomy or pneumonectomy	147 (25.1%)	80 (18.1%)	67 (46.9%)	54 (45.0%)	29 (24.2%)		
Sub-lobar resection	438 (74.9%)	362 (81.9%)	76 (53.1%)	66 (55.0%)	91 (75.8%)		
**Pathology (paraffin section)**						0.795	0.719
LUAD (AAH included)	542 (92.6%)	410 (92.8%)	132 (92.3%)	110 (91.7%)	109 (90.8%)		
LUSC	31 (5.3%)	24 (5.4%)	7 (4.9%)	6 (5.0%)	5 (4.2%)		
SCLC	4 (0.7%)	2 (0.5%)	2 (1.4%)	2 (1.7%)	2 (1.7%)		
LASC	1 (0.2%)	1 (0.2%)	0 (0.0%)	0 (0.0%)	1 (0.8%)		
LCC	1 (0.2%)	1 (0.2%)	0 (0.0%)	0 (0.0%)	0 (0.0%)		
Others	6 (1.0%)	4 (0.9%)	2 (1.4%)	1 (0.8%)	3 (2.5%)		
PC	2 (0.3%)	1 (0.2%)	1 (0.7%)	1 (0.8%)	1 (0.8%)		

LELC	1 (0.2%)	1 (0.2%)	0 (0.0%)	0 (0.0%)	1 (0.8%)		
NEC*	2 (0.3%)	1 (0.2%)	1 (0.7%)	0 (0.0%)	1 (0.8%)		
NUT	1 (0.2%)	1 (0.2%)	0 (0.0%)	0 (0.0%)	0 (0.0%)		
**Histologic subtypes (in 452 LUAD-IACs with 132 STAS(+))**						0.029	0.893
Lepidic	70 (15.5%)	47 (14.7%)	23 (17.4%)	20 (16.7%)	15 (12.9%)		
Acinar	190 (42.0%)	147 (45.9%)	43 (32.6%)	50 (41.7%)	55 (47.4%)		
Papillary	83 (18.4%)	61 (19.1%)	22 (16.7%)	20 (16.7%)	17 (14.7%)		
Micropapillary	45 (10.0%)	27 (8.4%)	18 (13.6%)	18 (15.0%)	15 (12.9%)		
Solid	35 (7.7%)	19 (5.9%)	16 (12.1%)	7 (5.8%)	8 (6.9%)		
CGP	10 (2.2%)	6 (1.9%)	4 (3.0%)	2 (1.7%)	3 (2.6%)		
**Variant histologic types (in 452 LUAD-IACs with 132 STAS(+))**						0.326	0.513
Mucinous	14 (3.1%)	11 (3.4%)	3 (2.3%)	2 (1.7%)	1 (0.9%)		
Fetal	2 (0.4%)	1 (0.3%)	1 (0.8%)	1 (0.8%)	1 (0.9%)		
Enteric	2 (0.4%)	1 (0.3%)	1 (0.8%)	0 (0.0%)	1 (0.9%)		
Colloid	1 (0.2%)	0 (0.0%)	1 (0.8%)	0 (0.0%)	0 (0.0%)		
**Pleural invasion**						<0.001	0.156
Present	99 (16.9%)	59 (13.3%)	40 (28.0%)	30 (25.0%)	21 (17.5%)		
Absent	486 (83.1%)	383 (86.7%)	103 (72.0%)	90 (75.0%)	99 (82.5%)		
**Lymphovascular invasion**						0.004	0.294
Present	105 (17.9%)	68 (15.4%)	37 (25.9%)	33 (27.5%)	26 (21.7%)		
Absent	480 (82.1%)	374 (84.6%)	106 (74.1%)	87 (72.5%)	94 (78.3%)		
***EGFR* mutation†**						0.570	0.087
Wild-type	158 (52.5%)	124 (51.6%)	34 (62.0%)	29 (60.4%)	23 (43.4%)		
Mutated	143 (47.5%)	116 (48.3%)	27 (38.0%)	19 (39.6%)	30 (56.6%)		
***ALK* rearrangement††**						0.002	0.609
Present	59 (10.0%)	28 (13.9%)	31 (28.7%)	26 (26.5%)	20 (23.3%)		
Absent	251 (81.0%)	174 (86.1%)	77 (71.3%)	72 (73.5%)	66 (76.7%)		
**CT signs**							
**Vascular convergence**						<0.001	0.010
Present	215 (36.8%)	133 (30.2%)	82 (57.2%)	71 (59.2%)	51 (42.5%)		
Absent	370 (63.2%)	309 (69.8%)	61 (42.8%)	49 (40.8%)	69 (57.5%)		
**Surrounding GGO**						<0.001	0.419
Present	337 (57.6%)	295 (66.7%)	42 (29.1%)	40 (33.3%)	46 (38.3%)		
Absent	248 (42.4%)	147 (33.3%)	101 (70.9%)	80 (66.7%)	74 (61.7%)		
**Air bronchogram**						0.816	0.121
Present	324 (55.4%)	246 (55.6%)	78 (54.5%)	70 (58.3%)	58 (48.3%)		
Absent	261 (44.6%)	196 (44.4%)	65 (45.5%)	50 (41.7%)	62 (51.7%)		
**Notch**						<0.001	0.682
Present	123 (21.0%)	68 (15.4%)	55 (38.4%)	42 (35.0%)	39 (32.5%)		
Absent	462 (79.0%)	374 (84.6%)	88 (61.5%)	78 (65.0%)	81 (67.5%)		
**Pleural indentation**						<0.001	0.035
Present	295 (50.4%)	196 (44.4%)	99 (69.2%)	91 (75.8%)	76 (63.3%)		
Absent	290 (49.6%)	246 (55.6%)	44 (30.8%)	29 (24.2%)	44 (36.7%)		

**Spiculation**						<0.001	0.003
Present	165 (28.2%)	98 (22.2%)	67 (46.9%)	55 (45.8%)	33 (27.5%)		
Absent	420 (71.8%)	344 (77.8%)	76 (53.1%)	65 (54.2%)	87 (72.5%)		
**Cavitation**						0.279	0.153
Present	56 (9.6%)	39 (8.9%)	17 (11.9%)	10 (8.3%)	17 (14.2%)		
Absent	529 (90.4%)	403 (91.1%)	126 (88.1%)	110 (91.7%)	103 (85.8%)		
**C/T ratio**						<0.001	0.867
Pure GGO	140 (23.9%)	130 (29.4%)	10 (7.0%)	4 (3.3%)	4 (3.3%)		
0 < C/T ⩽ 25%	52 (8.9%)	44 (10.0%)	8 (5.6%)	5 (4.2%)	7 (5.8%)		
25% < C/T ⩽ 50%	87 (14.9%)	69 (15.6%)	18 (12.6%)	16 (13.3%)	19 (15.8%)		
C/T > 50%	306 (52.3%)	199 (45.0%)	107 (74.8%)	95 (79.2%)	90 (75.0%)		

Abbreviations: LASC = adeno-squamous carcinoma of lung, LCC = large cell carcinoma of the lung, PC = pleomorphic carcinoma of the lung, LELC = lymphoepithelioma-like carcinoma, NEC = neuroendocrine cancer, CGP = complex glandular pattern.

Note: Chi-square calculations were only applied when all expected values were greater than 1.0 and at least 20% of the expected values were greater than 5. The chi-square test was used when the theoretical frequency was no less than 5 and the actual frequency was no less than 40; the chi-square test with continuity correction was used when 1 ⩽ theoretical frequency < 5 and actual frequency ⩾ 40; and the Fischer exact test was used when theoretical frequency < 1 or actual frequency < 40.

[#] AAH

[*] One patient was diagnosed with squamous carcinoma with neuroendocrine and was classified into LUSC.

[†] EGFR mutation status was evaluated for 301 patients.

[††] The results of ALK rearrangement detection were available in 310 patients.

Supplementary Table B3 | Baseline, surgical, and pathological information of patients (**ZS Cohort**)

**Variables**	**All patients** *n* = 36	**STAS (-)** *n* = 24	**STAS (+)** *n* =12	p value
**Gender**				0.499
Male	15 (41.6%)	9 (37.5%)	6 (50.0%)	
Female	21 (58.4%)	15 (62.5%)	6 (50.0%)	
**Age (year)**				0.316
Mean ± SD	59.1 ± 9.2	57.1 ± 5.6	60.5 ± 11.4	
Median (IQR)	61 [55, 69]	60 [53, 64]	65 [48, 69]	
**Smoking**				1.000
Never	22 (61.1%)	15 (62.5%)	7 (58.3%)	
Former/current	14 (38.9%)	9 (37.5%)	5 (41.7%)	
**pT stage (AJCC 8th edition)**				0.216
Tis	1 (2.8%)	1 (4.2%)	0 (0.0%)	
Tia (mis)	2 (5.6%)	2 (8.3%)	0 (0.0%)	
T1	27 (75.0%)	19 (76.0%)	8 (72.7%)	0.259
T1a	15 (41.7%)	12 (50.0%)	3 (25.0%)	
T1b	9 (25.0%)	6 (25.0%)	3 (25.0%)	
T1c	3 (8.3%)	1 (4.2%)	2 (18.2%)	
T2	3 (8.3%)	1 (4.2%)	2 (18.2%)	0.333
T2a	2 (5.6%)	0 (0.0%)	2 (18.2%)	
T2b	1 (2.8%)	1 (4.2%)	0 (0.0%)	
T3	1 (2.8%)	0 (0.0%)	1 (9.1%)	

T4	1 (2.8%)	1 (4.2%)	0 (0.00)	
Not applicable[#]	1 (2.8%)	1 (4.2%)	0 (0.00)	
**pN stage (AJCC 8th edition)**				0.367
N0	20 (55.6%)	15 (62.5%)	5 (41.7%)	
N1	9 (25.0%)	4 (16.7%)	5 (41.7%)	
N2	5 (13.9%)	3 (12.5%)	2 (16.7%)	
N3	1 (2.8%)	1 (4.2%)	0 (0.0%)	
Not applicable	1 (2.8%)	1 (4.2%)	0 (0.0%)	
**pM stage (AJCC 8th edition)**				0.343
M0	34 (94.4%)	23 (95.8%)	11 (91.7%)	
M1a	1 (2.8%)	0 (0.0%)	1 (8.3%)	
M1b	0 (0.0%)	0 (0.0%)	0 (0.0%)	
M1c	0 (0.0%)	0 (0.0%)	0 (0.0%)	
Not applicable	1 (2.8%)	1 (4.2%)	0 (0.0%)	
**Surgery**				0.729
Lobectomy or pneumonectomy	16 (44.4%)	10 (41.7%)	6 (50.0%)	
Sub-lobar resection	20 (55.6%)	14 (58.3%)	6 (50.0%)	
**Pathology (paraffin section)**				0.366
LUAD (AAH included)	31 (86.1%)	22 (91.7%)	9 (75.0%)	
LUSC	3 (8.3%)	1 (4.2%)	2 (16.7%)	
SCLC	2 (5.6%)	1 (4.2%)	1 (8.3%)	
**Histologic subtypes**				0.606
Lepidic	5 (13.9%)	4 (16.7%)	1 (8.3%)	
Acinar	11 (30.6%)	8 (33.3%)	3 (25.0%)	
Papillary	5 (13.9%)	3 (12.5%)	2 (16.7%)	
Micropapillary	3 (8.3%)	1 (4.2%)	2 (16.7%)	
Solid	1 (2.8%)	1 (4.2%)	0 (0.0%)	
Cribriform	0 (0.0%)	0 (0.0%)	0 (0.0%)	
**Variant histologic types**				/
Mucinous	2 (5.6%)	1 (4.2%)	1 (8.3%)	
Fetal	0 (0.0%)	0 (0.0%)	0 (0.0%)	
Enteric	0 (0.0%)	0 (0.0%)	0 (0.0%)	
Colloid	0 (0.0%)	0 (0.0%)	0 (0.0%)	
**Pleural invasion**				0.588
Present	4 (11.1%)	2 (8.3%)	2 (16.7%)	
Absent	32 (88.9%)	22 (91.7%)	10 (83.3%)	
**Lymphovascular invasion**				1.000
Present	5 (13.9%)	3 (12.5%)	2 (16.7%)	
Absent	31 (86.1%)	21 (87.5%)	10 (83.3%)	
***EGFR* mutation**[†]				1.000
Wild-type	18 (51.4%)	12 (52.2%)	6 (50.0%)	
Mutated	17 (48.6%)	11 (47.8%)	6 (50.0%)	
***ALK* rearrangement**[††]				0.253

Present	4 (11.4%)	2 (8.7%)	2 (16.7%)	
Absent	31 (88.6%)	21 (91.3%)	10 (83.3%)	
**CT signs**				
**Vascular convergence**				1.000
Present	8 (22.2%)	5 (20.8%)	3 (25.0%)	
Absent	28 (77.8%)	19 (79.2%)	9 (75.0%)	
**Surrounding GGO**				0.033
Present	17 (47.2%)	8 (33.3%)	9 (75.0%)	
Absent	19 (52.8%)	16 (66.7%)	3 (25.0%)	
**Air bronchogram**				0.471
Present	14 (38.9%)	8 (33.3%)	6 (50.0%)	
Absent	22 (61.1%)	16 (66.7%)	6 (50.0%)	
**Notch**				0.126
Present	9 (25.0%)	4 (16.7%)	5 (41.7%)	
Absent	27 (75.0%)	20 (83.3%)	7 (58.3%)	
**Pleural indentation**				0.148
Present	14 (38.9%)	7 (29.2%)	7 (58.3%)	
Absent	22 (61.1%)	17 (70.8%)	5 (41.7%)	
**Spiculation**				1.000
Present	6 (16.7%)	4 (16.7%)	2 (16.7%)	
Absent	30 (83.3%)	20 (83.3%)	10 (83.3%)	
**Cavitation**				0.543
Present	2 (5.6%)	2 (8.3%)	0 (0.0%)	
Absent	34 (94.4%)	22 (91.7%)	12 (100.0%)	
**C/T ratio**				0.890
Pure GGO	8 (22.2%)	5 (20.8%)	3 (25.0%)	
0 < C/T ⩽ 25%	2 (5.6%)	1 (4.2%)	1 (8.3%)	
25% < C/T ⩽ 50%	5 (13.9%)	3 (12.5%)	2 (16.7%)	
C/T > 50%	21 (58.3%)	15 (62.5%)	6 (50.0%)	

Abbreviations: LASC = adeno-squamous carcinoma of lung, LCC = large cell carcinoma of the lung, PC = pleomorphic carcinoma of the lung, LELC = lymphoepithelioma-like carcinoma, NEC = neuroendocrine cancer.

Note: The T-stage difference test was performed after combining Tis and Tia.

[#] AAH

[†] EGFR mutation status was evaluated for 35 patients.

[††] The results of ALK rearrangement detection were available in 35 patients.

Supplementary Table B4 | Baseline, surgical, and pathological information of patients (**Ninth Cohort**)

**Variables**	**All patients** *n* = 53	**STAS (-)** *n* = 36	**STAS (+)** *n* = 17	p value
**Gender**				0.441
Male	24 (45.3%)	15 (41.7%)	9 (52.9%)	
Female	29 (54.7%)	21 (58.3%)	8 (47.1%)	
**Age (year)**				0.3772
Mean ± SD	57.5 ± 9.8	57.1 ± 8.3	59.7 ± 11.2	
Median (IQR)	58 [52, 68]	60 [54, 69]	57 [53, 71]	

**Smoking**				0.430
Never	26 (49.1%)	19 (52.8%)	7 (41.2%)	
Former/current	27 (50.9%)	17 (47.2%)	10 (58.8%)	
**pT stage (AJCC 8th edition)**				0.113
Tis	2 (3.8%)	2 (5.6%)	0 (0.0%)	
Tia (mis)	2 (3.8%)	2 (5.6%)	0 (0.0%)	
T1	39 (73.6%)	27 (75.0%)	12 (70.6%)	0.292
T1a	22 (56.4%)	14 (51.9%)	8 (66.7%)	
T1b	13 (33.3%)	11 (40.7%)	2 (16.7%)	
T1c	4 (10.3%)	2 (7.4%)	2 (16.7%)	
T2	5 (9.4%)	1 (2.8%)	4 (23.5%)	1.000
T2a	2 (40.0%)	0 (0.0%)	2 (50.0%)	
T2b	3 (60.0%)	1 (100.0%)	2 (50.0%)	
T3	2 (3.8%)	1 (2.8%)	1 (5.9%)	
T4	1 (1.9%)	1 (2.8%)	0 (0.0%)	
Not applicable#	2 (3.8%)	2 (5.6%)	0 (0.0%)	
**pN stage (AJCC 8th edition)**				0.778
N0	31 (58.5%)	20 (55.6%)	11 (64.7%)	
N1	12 (22.6%)	9 (25.0%)	3 (17.7%)	
N2	8 (15.1%)	5 (13.9%)	3 (17.7%)	
N3	0 (0.0%)	0 (0.0%)	0 (0.0%)	
Not applicable	2 (3.8%)	2 (5.6%)	0 (0.0%)	
**pM stage (AJCC 8th edition)**				0.333
M0	50 (94.3%)	34 (94.4%)	16 (94.1%)	
M1a	1 (1.9%)	0 (0.0%)	1 (5.9%)	
M1b	0 (0.0%)	0 (0.0%)	0 (0.0%)	
M1c	0 (0.0%)	0 (0.0%)	0 (0.0%)	
Not applicable	2 (3.8%)	2 (5.6%)	0 (0.0%)	
**Surgery**				0.801
Lobectomy or pneumonectomy	20 (37.7%)	14 (38.9%)	6 (35.3%)	
Sub-lobar resection	33 (62.3%)	22 (61.1%)	11 (64.7%)	
**Pathology (paraffin section)**				0.142
LUAD (AAH included)	44 (84.6%)	32 (91.4%)	12 (70.6%)	
LUSC	5 (9.6%)	2 (5.7%)	3 (17.7%)	
SCLC	1 (1.9%)	1 (2.9%)	0 (0.0%)	
LASC	0 (0.0%)	0 (0.0%)	0 (0.0%)	
LCC	1 (1.9%)	0 (0.0%)	1 (5.9%)	
Others	1 (1.9%)	0 (0.0%)	1 (5.9%)	/
PC	0 (0.0%)	0 (0.0%)	0 (0.0%)	
LELC	0 (0.0%)	0 (0.0%)	0 (0.0%)	
NEC*	1 (100.0%)	0 (0.0%)	1 (100.0%)	
NUT	0 (0.0%)	0 (0.0%)	0 (0.0%)	
**Histologic subtypes**				0.524

Lepidic	5 (14.7%)	3 (12.5%)	2 (20.0%)	
Acinar	19 (55.9%)	15 (62.5%)	4 (40.0%)	
Papillary	6 (17.6%)	4 (16.7%)	2 (20.0%)	
Micropapillary	2 (5.9%)	1 (4.2%)	1 (10.0%)	
Solid	1 (2.9%)	0 (0.0%)	1 (10.0%)	
Cribriform	1 (2.9%)	1 (4.2%)	0 (0.0%)	
**Variant histologic types**				1.000
Mucinous	3 (75.0%)	2 (100.0%)	1 (50.0%)	
Fetal	1 (25.0%)	0 (0.0%)	1 (50.0%)	
Enteric	0 (0.0%)	0 (0.0%)	0 (0.0%)	
Colloid	0 (0.0%)	0 (0.0%)	0 (0.0%)	
**Pleural invasion**				0.231
Present	16 (30.2%)	9 (25.0%)	7 (41.2%)	
Absent	37 (69.8%)	27 (75.0%)	10 (58.8%)	
**Lymphovascular invasion**				0.995
Present	14 (26.4%)	10 (27.8%)	4 (23.5%)	
Absent	39 (73.6%)	26 (72.2%)	13 (76.5%)	
***EGFR* mutation**†				0.725
Wild-type	20 (54.1%)	14 (51.9%)	6 (60.0%)	
Mutated	17 (46.0%)	13 (48.2%)	4 (40.0%)	
***ALK* rearrangement**††				1.000
Present	10 (27.0%)	7 (25.9%)	3(30.00)	
Absent	27 (73.0%)	20 (74.1%)	7(70.00)	
**CT signs**				
**Vascular convergence**				0.647
Present	12 (22.64%)	7 (19.4%)	5 (29.4%)	
Absent	41 (77.4%)	29 (80.6%)	12 (70.6%)	
**Surrounding GGO**				0.548
Present	29 (54.7%)	8 (47.1%)	21 (58.3%)	
Absent	24 (45.3%)	9 (52.9%)	15 (41.7%)	
**Air bronchogram**				0.548
Present	25 (47.2%)	18 (50.0%)	7 (41.2%)	
Absent	28 (52.8%)	18 (50.0%)	10 (58.8%)	
**Notch**				0.085
Present	10 (18.9%)	4 (11.1%)	6 (35.3%)	
Absent	43 (81.1%)	32 (88.9%)	11 (64.7%)	
**Pleural indentation**				0.858
Present	29 (54.7%)	20 (55.6%)	9 (52.9%)	
Absent	24 (45.3%)	16 (44.4%)	8 (47.1%)	
**Spiculation**				0.647
Present	12 (22.6%)	7 (19.4%)	5 (29.4%)	
Absent	41 (77.4%)	29 (80.6%)	12 (70.6%)	
**Cavitation**				0.957

Present	8 (15.1%)	5 (13.9%)	3 (17.7%)	
Absent	45 (84.9%)	31 (86.1%)	14 (82.4%)	
**C/T ratio**				0.329
Pure GGO	8 (15.1%)	7 (19.4%)	1 (5.9%)	
0 < C/T ≤ 25%	3 (5.7%)	2 (5.6%)	1 (5.9%)	
25% < C/T ≤ 50%	7 (13.2%)	6 (16.7%)	1 (5.9%)	
C/T > 50%	35 (66.0%)	21(58.3%)	14 (82.4%)	

Abbreviations: LASC = adeno-squamous carcinoma of lung, LCC = large cell carcinoma of the lung, PC = pleomorphic carcinoma of the lung, LELC = lymphoepithelioma-like carcinoma, NEC = neuroendocrine cancer.

AAH

† EGFR mutation status was evaluated for 37 patients.

†† The results of ALK rearrangement detection were available in 37 patients.

Appendix B3 *The scan information/parameters of the involved centers*

The scan information (with parameters) of these three centers were:

(1) CHEST Cohort: The studies performed at Shanghai Chest Hospital used the following five scanners: a 64-detector row scanner (Brilliance, Philips, Cleveland, USA), a 256-detector row scanner (Revolution CT, GE, Waukesha, USA), a 128-detector row scanner (Ingenuity core 128, Philips, Suzhou, China), a Discovery CT750HD CT scanner (GE, Waukesha, USA), and a 16-detector row scanner (uCT S160, United Imaging, Shanghai, China). All CT examinations included the entire thorax at full suspended inspiration with the patient lying supine. The HRCT scans were performed with the following parameters: collimation, 0.625-1.25 mm; pitch, 0.64; section thickness 0.625-1.25 mm without overlap; matrix, 512×512 or 1024×1024; FOV, 350-400 mm; 120 kVp; and 220-300 mA. All imaging data were reconstructed by using a standard algorithm.

Two radiologists with 2 years' and 16 years' experience in chest radiology viewed the CT images to determine the C/T ratio and the CT signs.

(2) ZS Cohort： The studies performed at Shanghai Zhongshan Hospital utilized the Philips Brilliance 64-slice spiral CT scanner. Scanning parameters of the Siemens CT scanner were set as follows: tube voltage 120 KV, tube current 250 mA, pitch 1.1, reconstruction slice thickness 1 mm, reconstruction interval 1 mm, image matrix 512×512, filter function F. Philips CT scanner parameters: tube voltage 130 KV, tube current 200 mA, pitch 0.64, reconstruction slice thickness 1mm, reconstruction interval 1 mm, image matrix 512×512, filter function F. All patients underwent breath-holding training before scanning. During the scanning, breath-holding was required under free-breathing. The scan range was from the apex of the lung to the base of the lung, covering the axilla and chest wall on both sides. The bone algorithm was employed for reconstruction.

Two radiologists with 18 years' and 16 years' experience in chest radiology viewed the CT images to determine the C/T ratio and the CT signs. The 3D views extraction of tumor images for registration and VOIs for radiomics extraction were performed by the radiologist with 18 years' experience in chest radiology. The registrations were performed by an engineer with experiences in image registration.

(3) **Ninth Cohort**：The studies performed at Shanghai Ninth People's Hospital included a single-phase non-contrast or contrast-enhanced study with coverage from the base of the neck to include the adrenal glands inferiorly. It was performed with the patient in full inspiration on three CT scanners (SOMATOM Definition Flash, Siemens Healthineers, Erlangen, Germany; uCT 760, United Imaging, Shanghai, China; and Discovery CT750HD, Lightspeed 16 and VCT, GE Healthcare, Chicago, IL). The CT protocols of SOMATOM Definition Flash was as follows: 100 kVp, auto mA = 190 to 640; slice thickness 5 mm; pitch, 0.625; matrix, 512×512, and the reconstruction section thickness was 0.6 mm. The CT protocols of uCT 760 were as follows: 120 kVp, auto mA = 120 to 370; slice thickness 5 mm; pitch, 0.625; matrix, 512×512. The reconstruction section thickness was 1.0 mm. The CT protocols of Discovery CT750HD was as follows: 120 kVp, auto mA = 70 to 240; pitch, 0.984; matrix, 512×512, and the reconstruction section thickness was 1.25 mm.

Two radiologists with 18 years' and 16 years' experience in chest radiology viewed the CT images to determine the C/T ratio and the CT signs. The 3D views extraction of tumor images for registration and VOIs for radiomics extraction were performed by the radiologist with 16 years' experience in chest radiology. The registrations were performed by an engineer with experiences in image registration.

Appendix B4 *The acquisition of molecular profiles in CHEST Cohort*

As for the molecular profiles, Covaris M220 (Covaris, MA, USA) was used to shear DNA and fragments of size 200–400 base pairs (bp) were selected by bead (Agencourt AMPure XP Kit, Beckman Coulter, Brea, CA, USA). Qubit 2.0 fluorometer with the dsDNA high-sensitivity assay kit (Life Technologies, Carlsbad, CA, USA) was used to measure genomic DNA quality and concentration. Indexed samples were sequenced on Nextseq500 sequencer (Illumina, Inc., Madison, WI, USA) with pair-end reads. The genomic profiles were assessed with Lung Core panel from Burning Rock Biotech (Guangzhou, China) consisting of the whole exons of 68 lung cancer-related genes and spans 345 kb of the human genome.

For experimental protocol of molecular profiling for patients from Ninth Cohort and ZS Cohort, please contact the corresponding authors Dr. Yuan and Dr. Ye.

Appendix C | A detailed introduction to the radiomics and delta-radiomics features

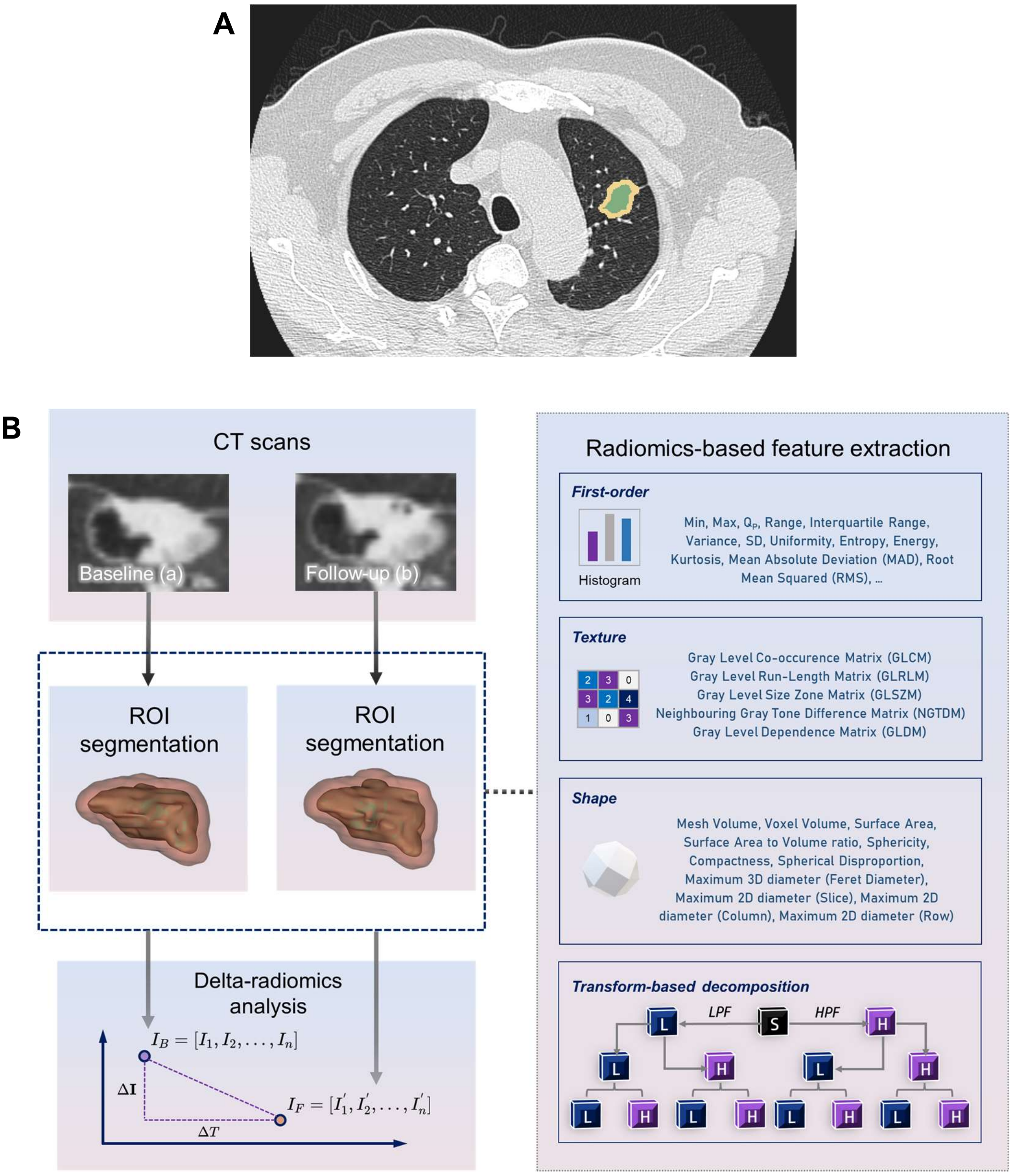

Supplementary Figure C | Pipeline of radiomics and delta-radiomics. A. An example of ROI delineation. **B.** The procedures of the delta-radiomics extraction and the feature categories.

In this study, we extracted a total of 851 radiomics features, of which 107 features were extracted before wavelet decomposition and the remaining 744 were wavelet transformed features. These wavelet-transformed features take a multi-resolution (multi-scale) view of the image features in a stepwise manner from coarse to fine, and they are the most frequently applied to the higher-order features in radiomics (**Supplementary Figure C-B**).

All features were extracted using 3D Slicer 5.0.3 and PyRadiomics v3.0 (**Supplementary Figure C-A**). The 107 raw features were classified into three categories: 18 first-order features, 14 shape features and 75 texture features. 75 texture features were categorized into five types, including Gray Level Co-occurrence Matrix (GLCM) features, Gray Level Co-occurrence Matrix (GLCM) features, Gray Level Run-Length Matrix (GLRLM) features, Gray Level Size Zone Matrix (GLSZM) features, and Neighboring Gray Tone Difference Matrix (NGTDM) features.

(1) First order features: First order features describe the distribution of voxel intensities in the image region of the ROI by common basic metrics. X is the set of all voxels in the ROI; N_p is the number of voxels in X; $P(i)$ is a first-order histogram with N_g discrete intensity levels, and $p(i)$ is a normalized first-order eigenvalue equal to $\frac{P(i)}{N_p}$; c is an optional value to prevent negative values in X.

(2) Shape: Shape features describe the size and shape of the ROI in two and three dimensions. N_v represents the number of voxels contained in the ROI; V represents the volume of the mesh in mm^3; A represents the surface area of the mesh in mm^2; P represents the perimeter of the mesh in mm. λ_{major}, λ_{minor} and λ_{least} are the largest, the second largest, and the smallest principal components of the ROI shape, respectively.

(3) Gray Level Co-occurrence Matrix (GLCM): It obtains spatial relationships of pixel or voxel pairs with predefined gray levels of intensity based on predefined distances between different directions and between pixels or voxels. Where:

ϵ is an arbitrarily small positive number ($\approx 2.2 \times 10^{-16}$);

$P(i,j)$ is an arbitrary covariance matrix;

$P(i,j)$ is a normalized covariance matrix equal to $\frac{P(i,j)}{\sum P(i,j)}$;

N_g is the number of discrete intensity levels in the image;

$p_x(i) = \sum_{j=1}^{N_g} p(i,j)$ is the row marginal probability;

$p_y(j) = \sum_{i=1}^{N_g} p(i,j)$ is the column marginal probability;

μ_x is the average gray level intensity defined as $\mu_x = \sum_{i=1}^{N_g} p_x(i)i$;

μ_y is the average gray level intensity, defined as $\mu_y = \sum_{j=1}^{N_g} p_y(j)j$;

σ_x is the standard deviation of p_x; σ_y is the standard deviation of p_y.

From this we have the joint probability distributions $p_{x+y}(k)$ and $p_{x-y}(k)$ as follows:

$$p_{x+y}(k) = \sum_{i=1}^{N_g} \sum_{j=1}^{N_g} p(i,j)$$

Where $i + j = k,\ \ k = 2, 3, \dots, 2N_g$.

$$p_{x-y}(k) = \sum_{i=1}^{N_g} \sum_{j=1}^{N_g} p(i,j)$$

Where $|i - j| = k,\ \ k = 0,1, \dots, N_g - 1$.

Meanwhile, we could obtain the entropy of p_x (HX), the entropy of p_y (HY), and the entropy of $p(i,j)$ (HXY):

$$HX = -\sum_{i=1}^{N_g} p_x(i)\, log_2(p_x(i) + \epsilon)$$

$$HY = -\sum_{j=1}^{N_g} p_y(j)\, log_2(p_y(j) + \epsilon)$$

$$HXY = -\sum_{i=1}^{N_g}\sum_{j=1}^{N_g} p(i,j)\, log_2(p(i,j) + \epsilon)$$

In order to quantify the complexity of textures, information metrics are further defined to measure the relevance:

$$HXY1 = -\sum_{i=1}^{N_g}\sum_{j=1}^{N_g} p(i,j)\, log_2\big(p_x(i)p_y(j) + \epsilon\big)$$

$$HXY2 = -\sum_{i=1}^{N_g}\sum_{j=1}^{N_g} p_x(i)p_y(j)\, log_2\big(p_x(i)p_y(j) + \epsilon\big)$$

(4) Gray Level Dependence Matrix (GLDM): It provides features based on the grayscale relationships between the central pixel or voxel and its neighbors, including magnitudes reflecting heterogeneity and homogeneity, as well as grayscale non-uniformity and dependent uniformity reflecting grayscale similarity and grayscale dependence across the ROI. N_g is the number of discrete intensity values in the image; N_d is the number of discrete dependencies in the image; N_z is the number of dependency regions in the image, which could be expressed as $\sum_{i=1}^{N_g}\sum_{j=1}^{N_d} P(i,j)$; $P(i,j)$ is the dependency matrix; $p(i,j)$ is the normalized dependency matrix, defined as:

$$p(i,j) = \frac{P(i,j)}{N_z}$$

(5) Gray Level Run-Length Matrix (GLRLM): It provides information about the spatial distribution of consecutive pixels with the same gray level in one or more directions, in 2 or 3 dimensions. N_g is the number of discrete intensity values in the image; N_r is the number of discrete run lengths in the image; N_p is the number of voxels in the image; $N_r(\theta)$ is the number of runs along the angle in the image:

$$N_r(\theta) = \sum_{i=1}^{N_g}\sum_{j=1}^{N_r} P(i,j|\theta), 1 \leq N_r(\theta) \leq N_p$$

$P(i,j|\theta)$ is the trip length matrix θ in any direction; $p(i,j|\theta)$ is the normalized trip length matrix defined as:

$$p(i,j|\theta) = \frac{P(i,j|\theta)}{N_r(\theta)}$$

(6) Gray Level Size Zone Matrix (GLSZM): GLSZM is based on the count of the number of groups of interconnected neighboring pixels or voxels (so-called zones) with the same gray level, where, let N_g be the number of discrete intensity values in the image; N_s is the number of discrete zone sizes in the image; N_p is the number of voxels in the image; N_z is the number of zones in the ROI:

$$N_z = \sum_{i=1}^{N_g}\sum_{j=1}^{N_s} P(i,j), 1 \leq N_z \leq N_p$$

$P(i,j)$ is the size zone matrix; $p(i,j)$ is the normalized size zone matrix, defined as:

$$p(i,j) = \frac{P(i,j)}{N_z}$$

(7) Neighboring Gray Tone Difference Matrix (NGTDM): It quantifies the sum of the differences between the gray level of a voxel and its average gray level of neighboring pixels or voxels within a predefined distance.

Supplementary Table C | The definitions and meanings of radiomics features

Categories in radiomics	Metrices	Definition	Meaning
First order	10th percentile	/	The 10th percentile value of X
	90th percentile	/	The 90th percentile value of X
	Energy	$Energy = \sum_{i=1}^{N_p} (\mathbf{X}(i) + c)^2$	Magnitude of voxel intensity in the image
	Entropy	$Entropy = -\sum_{i=1}^{N_g} log_2(p(i) + \epsilon)$	Uncertainty/randomness of image values
	Interquartile Range	$interquartilerange = \boldsymbol{P}_{75} - \boldsymbol{P}_{25}$	Mean value of grayscale values in ROI
	Kurtosis	$kurtosis = \frac{\mu_4}{\sigma^4} = \frac{\frac{1}{N_p}\sum_{i=1}^{N_p}(\boldsymbol{X}(i) - \bar{X})^4}{\left(\frac{1}{N_p}\sum_{i=1}^{N_p}(\boldsymbol{X}(i) - \bar{X})^2\right)^2}$	Concentration of voxel grayscale values
	Maximum	$maximum = max(\boldsymbol{X})$	Maximum value of grayscale
	Mean Absolute Deviation (MAD)	$Total_{energy} = V_{\text{voxel}} \sum_{i=1}^{N_p} (\mathbf{X}(i) + c)^2$	The average of the absolute value of the deviation of each gray value from the mean
	Mean	$mean = \frac{1}{N_p}\sum_{i=1}^{N_p} \boldsymbol{X}(i)$	Mean value of grayscale values
	Minimum	$minimum = min(\boldsymbol{X})$	Minimum value of grayscale value
	Range	$Total_{energy} = V_{\text{voxel}} \sum_{i=1}^{N_p} (\mathbf{X}(i) + c)^2$	Polar difference of grayscale values
	Robust Mean Absolute Deviation (rMAD)	$rMAD = \frac{1}{N_{10-90}} \sum_{i=1}^{N_{10-90}} \lvert \boldsymbol{X}_{10-90}(i) - \bar{X}_{10-90} \rvert$	Average distance of all voxel intensity values
	Root Mean Squared (RMS)	$RMS = \sqrt{\frac{1}{N_p}\sum_{i=1}^{N_p}(\boldsymbol{X}(i) + c)^2}$	Square root of the mean of the squared values of the intensity of each voxel
	Skewness	$skewness = \frac{\mu_3}{\sigma^3} = \frac{\frac{1}{N_p}\sum_{i=1}^{N_p}(\boldsymbol{X}(i) - \bar{X})^3}{\left(\sqrt{\frac{1}{N_p}\sum_{i=1}^{N_p}(\boldsymbol{X}(i) - \bar{X})^2}\right)^3}$	Asymmetry of the grayscale distribution with respect to the mean
Shape	Elongation	$elongation = \sqrt{\frac{\lambda_{minor}}{\lambda_{major}}}$	The relationship between the two largest principal components in the shape
	Flatness	$flatness = \sqrt{\frac{\lambda_{least}}{\lambda_{major}}}$	The relationship between the maximum and minimum principal components in the shape
	Least Axis Length	$leastaxis = 4\sqrt{\lambda_{least}}$	The minimum axis length of the surrounded ellipsoid calculated with the minimum principal component of the ROI

	Major Axis Length	$majoraxis = 4\sqrt{\lambda_{major}}$	The maximum axis length of the surrounded ellipsoid calculated with the maximum principal component of the ROI
	Maximum 2D diameter	/	Maximum pairwise Euclidean distance between ROI grid vertices in the sagittal plane
	Maximum 2D diameter (Slice)	/	Maximum pairwise Euclidean distance between ROI surface grid vertices in the axial plane
	Maximum 2D diameter (Column)	/	Maximum paired Euclidean distance between tumor surface grid vertices in the coronal plane
	Mesh Volume	$V_i = \frac{Oa_i \cdot (Ob_i \times Oc_i)}{6}, V = \sum_{i=1}^{N_f} V_i$	The volume calculated from the triangular mesh of ROI. Oa_i , Ob_i, Oc_i are the vectors from the origin to the three vertices of the triangular mesh respectively
	Minor Axis Length	$minoraxis = 4\sqrt{\lambda_{minor}}$	The length of the secondary long axis of the ellipsoid surrounded by the ROI calculated with the largest principal component
	Sphericity	$Sphericity = \frac{\sqrt[3]{36\pi V^2}}{A}$	A measure of the roundness of the ROI shape relative to the sphere, and a value of 1 indicates a perfect sphere
	Surface Area	$A_i = \frac{1}{2}\lvert a_i b_i \times a_i c_i \rvert, A = \sum_{i=1}^{N_f} A_i$	Surface area of ROI
	Surface Area to Volume ratio	$surfacetovolumeratio = \frac{A}{V}$	ROI surface area per unit volume
	Voxel Volume	$V_{voxel} = \sum_{k=1}^{N_v} V_k$	Approximate volume of ROI
GLCM	Autocorrelation	$autocorrelation = \sum_{i=1}^{N_g}\sum_{j=1}^{N_g} p(i,j)ij$	Measure texture fineness and roughness size
	Cluster Prominence	$cluster\ prominence = \sum_{i=1}^{N_g}\sum_{j=1}^{N_g}(i + j - \mu_x - \mu_y)^4 p(i,j)$	Measuring GLCM skewness and asymmetry
	Cluster Shade	$cluster\ shade = \sum_{i=1}^{N_g}\sum_{j=1}^{N_g}(i + j - \mu_x - \mu_y)^3 p(i,j)$	Measuring the skewness and uniformity of GLCM
	Cluster Tendency	$cluster\ tendency = \sum_{i=1}^{N_g}\sum_{j=1}^{N_g}(i + j - \mu_x - \mu_y)^2 p(i,j)$	Measure groupings of voxels with similar gray level values
	Contrast	$contrast = \sum_{i=1}^{N_g}\sum_{j=1}^{N_g}(i - j)^2 p(i,j)$	Measuring local voxel intensity changes

Correlation	$correlation = \frac{\sum_{i=1}^{N_G}\sum_{j=1}^{N_g} p(i,j)ij - \mu_x\mu_y}{\sigma_x(i)\sigma_y(j)}$	Linear dependence of grayscale values on their respective voxels in the GLCM
Difference Average	$difference\ average = \sum_{k=0}^{N_g-1} kp_{x-y}(k)$	Mean difference of voxel grayscale values
Difference Entropy	$difference\ entropy = \sum_{k=0}^{N_g-1} p_{x-y}(k)\, log_2\big(p_{x-y}(k)+\epsilon\big)$	Measure of randomness and variability
Difference Variance	$difference\ variance = \sum_{k=0}^{N_g-1} (k-DA)^2 p_{x-y}(k)$	Measure of heterogeneity
Inverse Difference (ID)	$ID = \sum_{k=0}^{N_g-1} \frac{p_{x-y}(k)}{1+k}$	Measuring image local homogeneity
Inverse Difference Moment (IDM)	$IDM = \sum_{k=0}^{N_g-1} \frac{p_{x-y}(k)}{1+k^2}$	Measuring image local homogeneity
Inverse Difference Moment Normalized (IDMN)	$IDMN = \sum_{k=0}^{N_g-1} \frac{p_{x-y}(k)}{1+\left(\frac{k^2}{N_g^2}\right)}$	Measuring image local homogeneity
Inverse Difference Normalized (IDN)	$IDN = \sum_{k=0}^{N_g-1} \frac{p_{x-y}(k)}{1+\left(\frac{k}{N_g}\right)}$	Measuring image local homogeneity
Informational Measure of Correlation (IMC) 1	$IMC1 = \frac{HXY - HXY1}{\max\{HX, HY\}}$	Measuring texture complexity
Informational Measure of Correlation (IMC) 2	$IMC2 = \sqrt{1-e^{-2(HXY2-HXY)}}$	Measuring texture complexity
Inverse Variance	$inverse\ variance = \sum_{k=1}^{N_g-1} \frac{p_{x-y}(k)}{k^2}$	Degree of clarity and regularity of the texture
Joint Average	$joint\ average = \mu_x = \sum_{i=1}^{N_g}\sum_{j=1}^{N_g} p(i,j)i$	Average gray intensity of the distribution
Joint Energy	$joint\ energy = \sum_{i=1}^{N_g}\sum_{j=1}^{N_g} (p(i,j))^2$	Measuring homogeneous patterns in an image
Contrast	$joint\ entropy = -\sum_{i=1}^{N_g}\sum_{j=1}^{N_g} p(i,j)\, log_2(p(i,j)+\epsilon)$	Measuring neighborhood intensity value randomness and variability
Maximal Correlation Coefficient (MCC)	$MCC = \sqrt{\text{second largest eigenvalue of } Q}$ $Q(i,j) = \sum_{k=0}^{N_g} \frac{p(i,k)p(j,k)}{p_x(i)p_y(k)}$	Measure of texture complexity

	Maximum Probability	$maximum\ probability = \max\big(p(i,j)\big)$	Maximum neighboring intensity values
	Sum Average	$sum\ average = \sum_{k=2}^{2N_g} p_{x+y}(k)k$	Relationship between lower intensity value pairs and higher intensity value pairs
	Sum Entropy	$sum\ entropy = \sum_{k=2}^{2N_g} p_{x+y}(k)\ log_2\big(p_{x+y}(k) + \epsilon\big)$	Sum of the differences in neighboring intensity values
	Sum of Squares	$sum\ squares = \sum_{i=1}^{N_g}\sum_{j=1}^{N_g}(i-\mu_x)^2 p(i,j)$	Distribution of neighboring intensity value pairs about the average intensity value in GLCM
GLDM	Dependence Entropy	$DependenceEntropy = -\sum_{i=1}^{N_g}\sum_{j=1}^{N_d} p(i,j)\ log_2(p(i,j)+\epsilon)$	Measuring the randomness of dependency size in an image
	Dependence Non-Uniformity (DN)	$DN = \frac{\sum_{j=1}^{N_d}\left(\sum_{i=1}^{N_g} P(i,j)\right)^2}{N_z}$	Measuring the similarity of dependencies in the whole image
	Dependence Non-Uniformity Normalized (DNN)	$DNN = \frac{\sum_{j=1}^{N_d}\left(\sum_{i=1}^{N_g} P(i,j)\right)^2}{N_z^2}$	Measuring the similarity of dependencies in the whole image
	Dependence Variance	$DV = \sum_{i=1}^{N_g}\sum_{j=1}^{N_d} p(i,j)(j-\mu)^2$ $\mu = \sum_{i=1}^{N_g}\sum_{j=1}^{N_d} jp(i,j)$	Measuring the difference of dependency size in an image
	Gray Level Non-Uniformity (GLN)	$GLN = \frac{\sum_{i=1}^{N_g}\left(\sum_{j=1}^{N_d} P(i,j)\right)^2}{N_z}$	Similarity of gray values in an image
	Gray Level Variance (GLV)	$GLV = \sum_{i=1}^{N_g}\sum_{j=1}^{N_d} p(i,j)(i-\mu)^2$ $\mu = \sum_{i=1}^{N_g}\sum_{j=1}^{N_d} ip(i,j)$	Difference in grayscale
	High Gray Level Emphasis (HGLE)	$HGLE = \frac{\sum_{i=1}^{N_g}\sum_{j=1}^{N_d} P(i,j)i^2}{N_z}$	Distribution of higher gray values
	Large Dependence Emphasis (LDE)	$LDE = \frac{\sum_{i=1}^{N_g}\sum_{j=1}^{N_d} P(i,j)j^2}{N_z}$	Distribution of large dependencies
	Large Dependence High Gray Level Emphasis (LDHGLE)	$LDHGLE = \frac{\sum_{i=1}^{N_g}\sum_{j=1}^{N_d} P(i,j)i^2j^2}{N_z}$	Joint distribution measuring large dependencies with higher gray values
	Large Dependence Low Gray Level Emphasis (LDLGLE)	$LDLGLE = \frac{\sum_{i=1}^{N_g}\sum_{j=1}^{N_d} \frac{P(i,j)j^2}{i^2}}{N_z}$	Joint distribution measuring large dependencies with lower gray values
	Low Gray Level Emphasis (LGLE)	$LGLE = \frac{\sum_{i=1}^{N_g}\sum_{j=1}^{N_d} \frac{P(i,j)}{i^2}}{N_z}$	Distribution of low gray values

	Small Dependence Emphasis (SDE)	$SDE = \frac{\sum_{i=1}^{N_g}\sum_{j=1}^{N_d}\frac{P(i,j)}{i^2}}{N_z}$	Distribution of small dependencies
	Small Dependence High Gray Level Emphasis (SDHGLE)	$SDHGLE = \frac{\sum_{i=1}^{N_g}\sum_{j=1}^{N_d}\frac{P(i,j)i^2}{j^2}}{N_z}$	Joint distribution measuring small dependencies with higher gray values
	Small Dependence Low Gray Level Emphasis (SDLGLE)	$SDLGLE = \frac{\sum_{i=1}^{N_g}\sum_{j=1}^{N_d}\frac{P(i,j)}{i^2j^2}}{N_z}$	Joint distribution measuring small dependencies with lower gray values
GLRLM	Gray Level Non-Uniformity (GLN)	$GLN = \frac{\sum_{i=1}^{N_g}\left(\sum_{j=1}^{N_r}P(i,j\|\theta)\right)^2}{N_r(\theta)}$	Measuring the similarity of gray-level intensity values in an image
	Gray Level Non-Uniformity Normalized (GLNN)	$GLNN = \frac{\sum_{i=1}^{N_g}\left(\sum_{j=1}^{N_r}P(i,j\|\theta)\right)^2}{N_r(\theta)^2}$	Measures the similarity of gray-level intensity values in an image
	Gray Level Variance (GLV)	$GLV = \sum_{i=1}^{N_g}\sum_{j=1}^{N_r}p(i,j\|\theta)(i-\mu)^2$	Measures the variance of the gray level intensity of the run
	High Gray Level Run Emphasis (HGLRE)	$HGLRE = \frac{\sum_{i=1}^{N_g}\sum_{j=1}^{N_r}P(i,j\|\theta)i^2}{N_r(\theta)}$	Measuring the similarity of gray-level intensity values in an image
	Long Run Emphasis (LRE)	$LRE = \frac{\sum_{i=1}^{N_g}\sum_{j=1}^{N_r}P(i,j\|\theta)j^2}{N_r(\theta)}$	Measures the similarity of gray-level intensity values in an image
	Long Run High Gray Level Emphasis (LRHGLE)	$LRHGLRE = \frac{\sum_{i=1}^{N_g}\sum_{j=1}^{N_r}P(i,j\|\theta)i^2j^2}{N_r(\theta)}$	Measures the variance of the gray level intensity of the run
	Long Run Low Gray Level Emphasis (LRLGLE)	$LRLGLRE = \frac{\sum_{i=1}^{N_g}\sum_{j=1}^{N_r}\frac{P(i,j\|\theta)j^2}{i^2}}{N_r(\theta)}$	A measure of the distribution of higher grayscale values, with higher values indicating a greater concentration of higher grayscale values in the image
	Low Gray Level Run Emphasis (LGLRE)	$LGLRE = \frac{\sum_{i=1}^{N_g}\sum_{j=1}^{N_r}\frac{P(i,j\|\theta)}{i^2}}{N_r(\theta)}$	A measure of the distribution of small dependencies, with larger SDE indicating inhomogeneous texture
	Run Entropy (RE)	$RE = -\sum_{i=1}^{N_g}\sum_{j=1}^{N_r}p(i,j\|\theta)\,log_2(p(i,j\|\theta)+\epsilon)$	A measure of the joint distribution of long travel lengths with high gray values
	Run Length Non-Uniformity (RLN)	$RLN = \frac{\sum_{j=1}^{N_r}\left(\sum_{i=1}^{N_g}P(i,j\|\theta)\right)^2}{N_r(\theta)}$	A measure of the joint distribution of long travel lengths with lower gray values
	Run Length Non-Uniformity Normalized (RLNN)	$RLNN = \frac{\sum_{j=1}^{N_r}\left(\sum_{i=1}^{N_g}P(i,j\|\theta)\right)^2}{N_r(\theta)^2}$	Measures the distribution of low grayscale values, with higher values indicating higher concentrations of low grayscale values in the image
	Run Percentage (RP)	$RP = \frac{N_r(\theta)}{N_p}$	Uncertainty and randomness of the distribution of run lengths and gray levels, with higher values indicating greater heterogeneity of texture patterns

	Run Variance (RV)	$RV = \sum_{i=1}^{N_g}\sum_{j=1}^{N_r} p(i,j\|\theta)(j-\mu)^2$	A measure of the distribution of small dependencies, with larger SDE indicating texture inhomogeneity
	Short Run Emphasis (SRE)	$SRE = \frac{\sum_{i=1}^{N_g}\sum_{j=1}^{N_r}\frac{P(i,j\|\theta)}{j^2}}{N_r(\theta)}$	A measure of the distribution of small dependencies, with larger SDE indicating inhomogeneous texture
	Short Run High Gray Level Emphasis (SRHGLE)	$SRHGLE = \frac{\sum_{i=1}^{N_g}\sum_{j=1}^{N_r}\frac{P(i,j\|\theta)i^2}{j^2}}{N_r(\theta)}$	A measure of the roughness of the texture
	Short Run Low Gray Level Emphasis (SRLGLE)	$SRLGLE = \frac{\sum_{i=1}^{N_g}\sum_{j=1}^{N_r}\frac{P(i,j\|\theta)}{i^2 j^2}}{N_r(\theta)}$	A measure of travel variance of travel lengths
	Gray Level Non-Uniformity (GLN)	$GLN = \frac{\sum_{i=1}^{N_g}\left(\sum_{j=1}^{N_d} P(i,j)\right)^2}{N_z}$	A measure of short-term travel length distribution, larger indicates shorter travel length with finer texture
GLSZM	Gray Level Non-Uniformity (GLN)	$GLN = \frac{\sum_{i=1}^{N_g}\left(\sum_{j=1}^{N_s} P(i,j)\right)^2}{N_z}$	A measure of the distribution of small dependencies, with larger SDE indicating uneven texture
	Gray Level Non-Uniformity Normalized (GLNN)	$GLNN = \frac{\sum_{i=1}^{N_g}\left(\sum_{j=1}^{N_s} P(i,j)\right)^2}{N_z^2}$	Joint distribution of shorter travel lengths with lower grayscale values
	Gray Level Variance (GLV)	$GLV = \sum_{i=1}^{N_g}\sum_{j=1}^{N_s} p(i,j)(i-\mu)^2$ $\mu = \sum_{i=1}^{N_g}\sum_{j=1}^{N_s} p(i,j)i$	A measure of the similarity of grayscale intensity values in an image, the lower the GLN value, the greater the similarity of intensity values
	High Gray Level Zone Emphasis (HGLZE)	$HGLZE = \frac{\sum_{i=1}^{N_g}\sum_{j=1}^{N_s} P(i,j)i^2}{N_z}$	Measures the variability of grayscale intensity values in an image, the lower the GLN value the greater the uniformity of intensity values
	Large Area Emphasis (LAE)	$LAE = \frac{\sum_{i=1}^{N_g}\sum_{j=1}^{N_s} P(i,j)j^2}{N_z}$	Measure the variability of gray intensity values in the image, the lower the GLN, the greater the uniformity of intensity values
	Large Area High Gray Level Emphasis (LAHGLE)	$LAHGLE = \frac{\sum_{i=1}^{N_g}\sum_{j=1}^{N_s} P(i,j)i^2 j^2}{N_z}$	Variance of ROI gray level intensity
	Large Area Low Gray Level Emphasis (LALGLE)	$LALGLE = \frac{\sum_{i=1}^{N_g}\sum_{j=1}^{N_s}\frac{P(i,j)j^2}{i^2}}{N_z}$	Measures the distribution of higher gray level values, with higher indicating a greater proportion of high gray areas in the image
	Low Gray Level Zone Emphasis (LGLZE)	$LGLZE = \frac{\sum_{i=1}^{N_g}\sum_{j=1}^{N_s}\frac{P(i,j)}{i^2}}{N_z}$	A measure of the distribution over large areas, with higher values indicating rougher texture over larger areas
	Size-Zone Non-Uniformity (SZN)	$SZN = \frac{\sum_{j=1}^{N_s}\left(\sum_{i=1}^{N_g} P(i,j)\right)^2}{N_z}$	A measure of the joint distribution of larger size areas with higher gray levels in the image
	Size-Zone Non-Uniformity Normalized (SZNN)	$SZNN = \frac{\sum_{j=1}^{N_s}\left(\sum_{i=1}^{N_g} P(i,j)\right)^2}{N_z^2}$	A measure of the joint distribution of larger areas in the image with lower gray levels

	Small Area Emphasis (SAE)	$SAE = \frac{\sum_{i=1}^{N_g}\sum_{j=1}^{N_s}\frac{P(i,j)}{j^2}}{N_z}$	Distribution of areas with lower gray levels, with higher indicating a larger proportion of areas with lower gray values in the image
	Small Area High Gray Level Emphasis (SAHGLE)	$SAHGLE = \frac{\sum_{i=1}^{N_g}\sum_{j=1}^{N_s}\frac{P(i,j)i^2}{j^2}}{N_z}$	Variability of the volume of the size region in the image, with lower values indicating greater uniformity in the image
	Small Area Low Gray Level Emphasis (SALGLE)	$SALGLE = \frac{\sum_{i=1}^{N_g}\sum_{j=1}^{N_s}\frac{P(i,j)}{i^2j^2}}{N_z}$	Variability of the volume of the size region in the image, with lower values indicating greater homogeneity in the image
	Size-Zone Non-Uniformity (SZN)	$ZE = -\sum_{i=1}^{N_g}\sum_{j=1}^{N_s} p(i,j)\, log_2(p(i,j)+\epsilon)$	A measure of the distribution of small size areas
	Size-Zone Non-Uniformity (SZN)	$ZP = \frac{N_z}{N_p}$	Measure of the joint distribution of smaller size areas with higher gray levels in the image
	Zone Variance (ZV)	$ZV = \sum_{i=1}^{N_g}\sum_{j=1}^{N_s} p(i,j)(j-\mu)^2$ $\mu = \sum_{i=1}^{N_g}\sum_{j=1}^{N_s} p(i,j)j$	A measure of the proportion of the joint distribution of smaller sized regions with lower gray levels in the image
NGTDM	Busyness	$Busyness = \frac{\sum_{i=1}^{N_g} p_i s_i}{\sum_{i=1}^{N_g}\sum_{j=1}^{N_g}\lvert ip_i - jp_j\rvert}$ $p_i \neq 0, p_j \neq 0$	Measures the uncertainty and randomness of region size and grayscale distribution. Higher values indicate greater heterogeneity of texture patterns
	Coarseness	$Coarseness = \frac{1}{\sum_{i=1}^{N_g} p_i s_i}$	Measures the roughness of the texture
	Complexity	$Complexity = \frac{1}{N_{v,p}}\sum_{i=1}^{N_g}\sum_{j=1}^{N_g}\lvert i-j\rvert \frac{p_i s_i + p_j s_j}{p_i + p_j}$ $p_i \neq 0, p_j \neq 0$	Measures the roughness of the texture
	Contrast	$contrast = \sum_{i=1}^{N_g}\sum_{j=1}^{N_g}(i-j)^2 p(i,j)$	A measure of the variation from a pixel to its neighboring pixels
	Strength	$Strength = \frac{\sum_{i=1}^{N_g}\sum_{j=1}^{N_g}(p_i+p_j)(i-j)^2}{\sum_{i=1}^{N_g} s_i}$ $p_i \neq 0, p_j \neq 0$	A measure of the average difference between the central voxel and its neighbors, as the spatial rate of change

Supplementary Table C gives a summary of the interpretability and definition of the defined radiomics features in this study.

Appendix D | Concrete procedures for deep learning-based feature extraction

The machine learning model applied in this paper is based on the concept of "dynamic follow-up". The structure of the model used in this paper is presented in the figure below. It can be seen that the model in this paper is a novel "double/dual-delta machine learning model", unlike the previous classic radiomics and deep learning models. By “double/dual-delta”, we meant that the two components of the extracted features of the model were derived from the delta-radiomics model and the delta-deep learning model, respectively. The delta-radiomics model was based on the classic concept of radiomics model, which derived the time-based slope from the radiomics information extracted from a pair of follow-up CTs, and then described the incremental changes of each metric to reflect the characteristics of tumor growth during a certain follow-up interval. It had the advantages of quantification, refinement and good interpretability, but the disadvantage was that it is not intuitive (It could not be described by feature maps that visualize the model attentions). Delta-DL model is a concept proposed based on image registration and subtraction, which delineates nodes on two follow-up CTs separately and extracted 2D ROIs, and then performed registrations based on the feature points, intensity information, etc. After image subtraction, it could reflect the anisotropy of tumor growth and cavity changes, necrosis and calcification inside the tumor within a certain period of time. After extracting features by convolutional neural network (CNN), it can assess the tumor growth in the form of feature maps, which was more intuitive than classic radiomics, but the disadvantage was that it was relatively less interpretable and insensitive to the difference in follow-up time. The combination of the two sub-models provides a more comprehensive picture of tumor growth information.

Likewise, we still extracted the single visit information (namely the classic DL and radiomics features) from the follow-up visits (since they were acquired at a time closer to the final surgical pathological assessment than the baseline scans) as classic features and fused them into the final feature set. Therefore, as seen in the lower right corner of **Figure 3A**, our feature set has four categories: (1) Delta features based on deep learning (DL): it is based on the concept of registration and subtraction. After comparing the nodal morphology on the two follow-up CT visits (segmented ROIs with masked out-of-tumor areas needed to eliminate the influence of the surrounding area on the registration), a transformation matrix can be extracted and imposed on one of the CT images, thus eliminating the influence of body position and pose on image subtraction. Then, the transformed CT information was subtracted from the other baseline CT to obtain the subtracted images, which was further fed into the deep CNN to extract the delta-DL feature set (the concrete process shown in **Supplementary Figure D1**); (2) Classic DL features: these features were extracted by the classic deep learning CNN model, and the features were taken out from the fully connected layer; (3) Delta features based on radiomics: the time-based slope of radiomics features extracted from baseline and follow-up scans, respectively; (4) Classic radiomics features (i.e., radiomics features extracted from follow-up CTs that were closer to surgery).

This section will systematically introduce the concrete procedures for deep learning-based feature extraction in three steps: ***Image masking process***, ***Registration***, and ***Feature extraction based on CNN***.

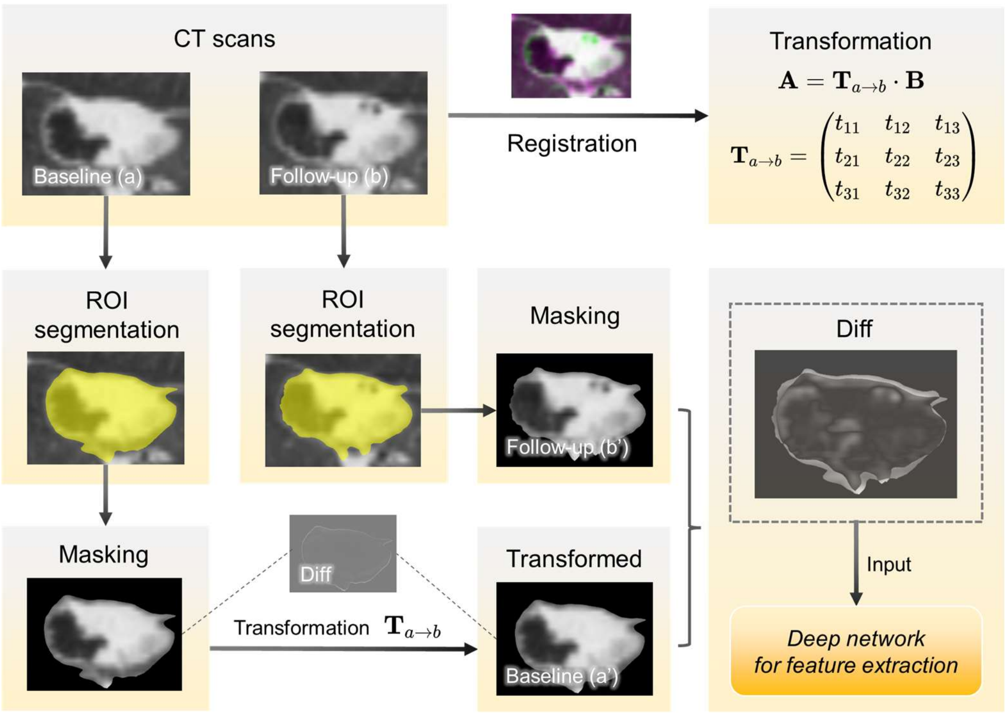

Supplementary Figure D1 | Pipeline of delta-DL feature extraction.

a. Image masking process

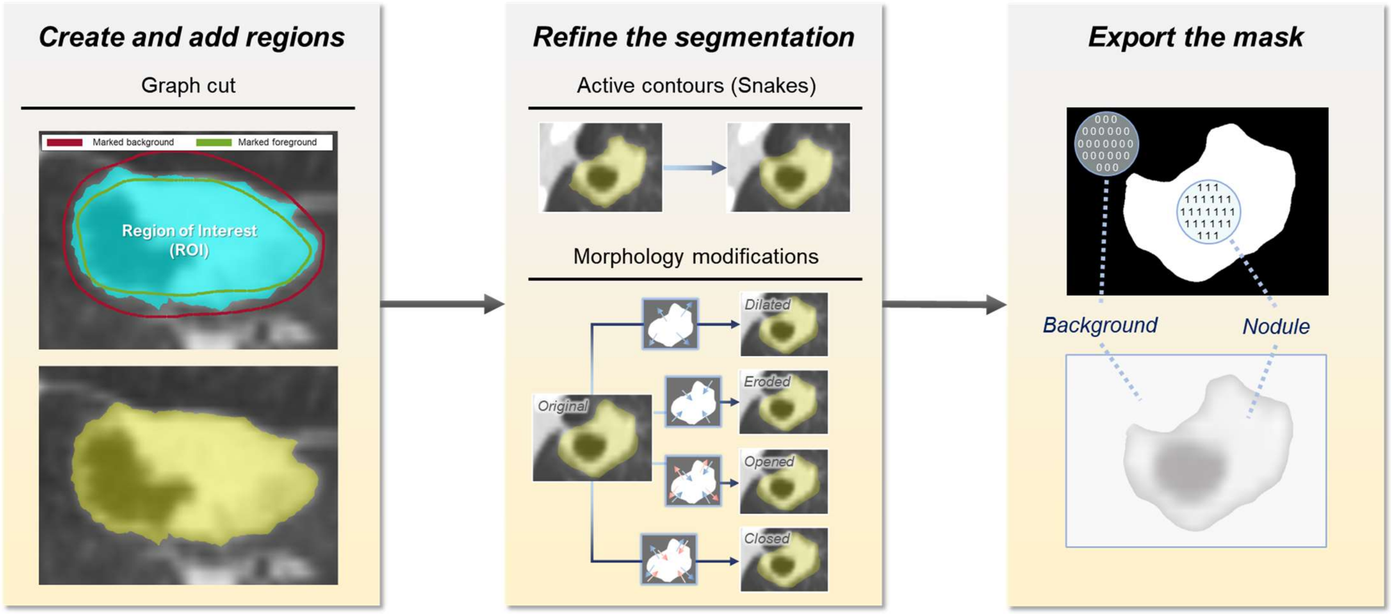

Supplementary Figure D2 | The image masking process in this work.

In order to accurately complete the registration, it is necessary to remove background information that is not relevant to the nodule, namely the process of masking. This step is illustrated above. We applied a combination of Graph Cut and ROI manual segmentation to initially build the ROI area, and then refined the area with the SNAKES (i.e. the active contouring) algorithm. The refinement is complemented by four morphological operations (i.e. erode, dilate, open, close) to eliminate and fill specific areas. The result of this step is a mask matrix, which is multiplied by the elements of the original image to obtain a masked ROI for subsequent alignment.

The Graph Cut is a semi-automatic tool segmenting the foreground and background. Here we provide A

summary of the process for readers to understand the principles of Graph Cut (a graphical illustration given in **Supplementary Figure D3**).

a. First, the user is required to specify a small number of background pixels B and foreground target pixels O.
b. The image is then built as a graph, where pixels are nodes on the graph.
c. Two special nodes are T node (background terminal) and S node (object terminal, foreground).
d. Connections are established between all nodes. Connections between image pixels are called n-links and connections from pixels to special nodes are called t-links.
e. The energy of t-links represents how much a pixel resembles the foreground or background. All pixels that have been labeled as foreground or background in the **Supplementary Figure D3** below have thick t-links. N-links energy constrains the pixels to be assigned to different regions. If the more likely neighboring nodes are to be separated, the thinner the connection and the lower the energy will be. If the difference between the two neighboring pixels is very small, it is very likely that both pixels belong to the foreground (namely the ROI) or both belong to the background, namely the greater the weight of the edge between the two pixels will be and they are less likely to be segmented; if the difference between the two neighboring pixels is very large, it means that the two pixels are likely to be in the edge part of the ROI and the background, and then the less the weight of the edge between the two pixels, the possibility to be segmented is higher.
f. In order to split the foreground and background, the Graph Cut will end up using the min-cut method to find a slit that minimizes the total energy of the cut connections.

The above procedures become clearer when the principle of Graph Cut is introduced. We have goal/energy/loss/weight function $E(A)$ determined by two terms $R(A)$ and $B(A)$:

$$E(A) = \lambda \cdot R(A) + B(A)$$

$$R(A) = \sum_{p \in P} R_p(A_p)$$

$$B(A) = \sum_{\{p,q\} \in N} B_{\{p,q\}} \cdot \delta(A_p, A_q), \text{ where } \delta(A_p, A_q) = \begin{cases} 1 & \text{if } A_p \neq A_q \\ 0 & \text{otherwise} \end{cases}$$

Meanwhile, we have the following hard constraints:

$$\forall p \in O, A_p = \text{object}$$

$$\forall q \in B, A_p = \text{background}$$

Where $R(A)$ denotes the regional data term of segmentation A, $B(A)$ denotes the boundary smoothing term of segmentation A, and $E(A)$ denotes the weights, i.e., the loss function, also called the energy function, and the goal of graph cutting is to optimize the energy function to minimize its value. λ is the important factor between the region term and the boundary term, which determines the magnitude of their influence on the energy. If λ is 0, only the boundary factor is considered and not the region factor. The regional term $R(A)$ assumes that the individual penalties for assigning pixel p to "object" and "background", correspondingly

R_p(“object”) and R_p(“background”), are given. The term $B(A)$ defined the “boundary” properties of the segmentation A, where the non-negative $B_{\{p,q\}}$ could be interpreted as a penalty for a discontinuity between p and q. When the $B_{\{p,q\}}$ is large, it denotes that the pixels p and q are similar. The penalty $B_{\{p,q\}}$ can also decrease as a function of distance between p and q. The goal of Graph Cut is to compute the global minimum of $R(A)$ among all segmentations A satisfying the hard constraints, where the constraints were determined by the initial strokes/lines/labels given by the operators.

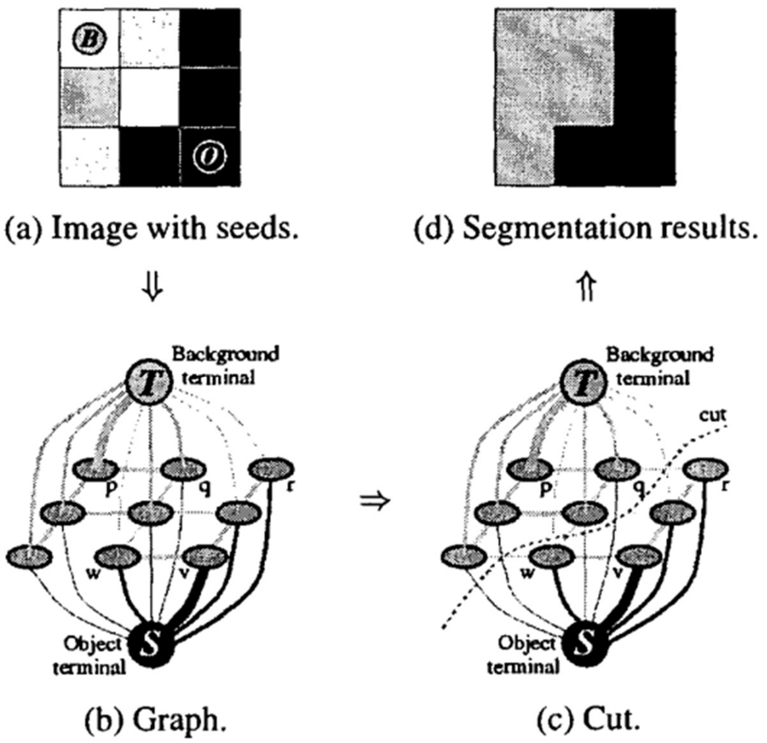

Supplementary Figure D3 | A simple 2D segmentation example for a 3 x 3 image (the original figure from literature *Interactive Graph Cuts for Optimal Boundary & Region Segmentation of Objects in N-D Images* **by Yuri Y. Boykov et al.).** The seeds are $O = \{w\}$ and $B = \{p\}$. The cost of each edge is reflected by the edge’s thickness. The regional term $E(A)$ and hard constraints ($\forall p \in O, A_p = \text{object}, \forall q \in B, A_p = \text{background}$) define the costs of t-links. The boundary term $B(A)$ defines the costs of n-links. Inexpensive edges are attractive choices for the minimum cost cut[11].

The Image Segmenter app in MATLAB provides access to many different ways to segment an image. The active contour method, also known as snake, is an iterative region-growing image segmentation algorithm. We applied the Gabor filter to reserve the texture features in the graphs. The Gabor transform is a special case of the short-time Fourier transform when the window function is taken to be a Gaussian function, which can extract relevant features at different scales and in different directions in the frequency domain. Its role is similar to that of the human eye and it has an important role in texture recognition. Two-dimensional Gabor filters were applied for the extraction of the texture features in order to assist the segmentation with the texture information. Generally, in the spatial domain, a Gabor filter could be given by:

$$G_{\lambda\theta\psi\sigma\gamma}(x, y) = \mathrm{e}^{-\frac{x'^2+\gamma^2 y'^2}{2\sigma^2}} \cos\left(2\pi\frac{x'}{\lambda} + \psi\right)$$

The aspect ratio γ determines the ellipticity of the shape of the Gabor function. When $\gamma = 1$, the shape is

circular; when $\gamma < 1$, the shape elongates with the direction of the parallel stripes. The value was 0.5 in our work. The wavelength λ represents the wavelength parameter of the cosine function in the Gabor kernel function. Its value is formulated in pixels. The direction θ indicates the direction of the parallel strips in the Gabor filter kernel. Valid values are real numbers from 0° to 360°. The phase shift ψ represents the phase parameter of the cosine function in the Gabor kernel function. It takes values in the range -180° to 180°. The half-response spatial frequency bandwidth b of the Gabor filter is related to the ratio σ/λ, where σ denotes the standard deviation of the Gaussian factor of the Gabor function. These parameters followed the default settings in MATLAB Image Segmenter. For the detail of this toolbox, please refer to https://ww2.mathworks.cn/help/images/image-segmentation-using-the-image-segmenter-app.html.

b. Registration

Registration is the process of finding the spatial mapping between the pixels of one image to the pixels of another image. These images can be taken at different times (multitemporal registration) and in different places by different sensors (multimodal registration). Since the beginning of the 21st century, image registration has mainly used feature-based methods. These methods have three steps: keypoint detection and feature description, feature matching, and image transformation. In simple terms, we selected the points of interest in two images, associated the reference image with the equivalent points of interest in the sensed image, and then transformed the sensed image to align the two images. Generally speaking, image registration includes the following steps: (1) **Model selection**: it is required to select a suitable transformation model according to the specific scenario of the application; (2) **Feature space selection**: the common feature space includes corner points, spots, specific intensity regions, etc.; (3) **maximum correlation point finding**: according to the selected transformation model and the corresponding features, the search strategy is selected and optimized where the similarity measure is applied;(4) **Transformation model solving**: based on the correspondence of the maximum correlation points, the unknown parameters are solved and the transform model is estimated and evaluated; (5) **Image transformation**: according to the transformation model, the image will be aligned with the reference image correspond to each other. These core processes are the same in all types of registration methods.

There are differences in the use of various transformation methods, but basically, they are divided into two sequential steps: feature detection and transformation calculation. Based on the following considerations, we used the classic **2D Transformations** instead of 3D ones, since the registration of the 3D point cloud requires complex computational resources and there is usually some error in 3D point cloud registrations due to the variability of breathing depth, spinal curvature, and body postures, etc. In addition, the classic 2D Transformations allow physicians to have a more intuitive understanding to participate in the selection of the pre-registration image pairs to describe the dynamic changes of tumor area and the evaluation of the quality after alignment to optimize the engineering parameters and settings. Therefore, the 3D registration was not used in this study. Instead, we adopted the classic 2D transformation for registration and further image subtraction. The forms of transformations include rigid, similarity, affine, and projective. In the rigid transformation (namely the Euclidean transformation), the most important feature is that the distances between any two points (before and after the transformation, respectively) of the targets remains unchanged,

which has 3 degrees of freedom. In the similarity transformation, the shapes of the target before and after the transformation remain the same, and in essence, the components of the isometric transformation are added into the conventional rigid transformation. The similarity transformation has four degrees of freedom. In the affine transformation, the order of the points remains the same and the parallel lines in the image remain parallel after the transformation, thus the degree of freedom is 6. The projective transformation is the most complex, it is a linear transformation of the homogeneous ternary vectors, and the degree of freedom is 8.

The terms used in this study follows the custom in engineering research, and the image to be registered is called "moving image" (in this study, it actually refers to the images extracted from the follow-up scans), which undergoes a global transformation (i.e., the classic 2D transformation pattern noted above) to provide the best registration results according to the corresponding features with the fixed image (in this study, it is the images extracted from the baseline scans).

The rotation, scaling, translation and other affine transformations are essentially linear transformations, which are essentially a class of global transformations with an inherent disadvantage in describing local geometric differences between images. This problem could be well addressed by non-rigid transformations that can locally distort the image to align it with the reference image. Strictly speaking, non-rigid registration is a local alignment technique, which is no longer limited to classic global transformations such as rotation, translation, scaling, etc., but is achieved by examining all the local details of the differences between the two images, so that, in general, non-rigid registrations uses spatial transformations with higher degrees of freedom, which may even exceed a million units of freedom in three-dimensional situations. In this study, the non-rigid transformation and the displacement field formed by the elastic transformation are not routinely applied in this study, considering that the growth of lung nodules is anisotropic, and the displacement field formed by the elastic transformation may eliminate this feature. This is an important difference between this study and the classic image registration task, because the use of displacement fields may affect the characterization of the heterogeneity in lung tumor growth.

Many kinds of algorithms are used for feature detection in image registration, and several of the most representative and general classes are used in this study. The algorithms used in this study are indicated in the following **Supplementary Table D**. Although they differ in principle, they can be basically divided into the following three categories: (1) Feature-based registration techniques: they automatically detect obvious image features, such as sharp corners, speckles, or regions with uniform intensity, which include algorithms with a high degree of automaticity such as SIFT, but also algorithms that can adjust the number and quality of matched features such as Harris, FAST, etc. Taking Harris for example, it detects the corner points of the image, which is one of the most basic types in feature points. (2) Intensity-based registration algorithms, and (3) Non-rigid algorithms. At the same time, various registration methods have their own special features, which are related to various factors such as scale space, feature class, and filter function. For example, in general, algorithms such as SURF construct a linear scale space and detect feature points in it, which tends to cause blurred image boundaries and may lose some image details, but the KAZE algorithm detects feature points in images by constructing a nonlinear scale space, which allows more image details to be preserved. In this study, a total of 12 image alignment methods are compared, including nine feature-based registration methods

and three intensity-based registration methods (see **Supplementary Table D**).

In a broad sense, registration is the process of aligning the spatial position of images taken at different times, backgrounds and angles by the same sensor, and at the same time by different sensors[12]. In general, the results of image alignment can be expressed in terms of affine transformations. Affine transformations include geometric contraction, expansion, dilation, reflection, rotation, shear, similarity transformations, spiral similarities and translation (some common affine transformations were illustrated tin the **Supplementary Table D-A**). Typically, affine transformations can often be a combination of rotations, translations, dilations and shears. In this study, four transformations were utilized for registration (rigid, similarity, affine, and projective).

Supplementary Table D | The definitions and meanings of radiomics features

(A) Common affine transformations and their matrices

Transformation	Affine matrix	Graphical illustration
Identity	$\begin{bmatrix}1 & 0 & 0\\0 & 1 & 0\\0 & 0 & 1\end{bmatrix}$	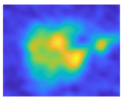
Translation	$\begin{bmatrix}1 & 0 & v_x\\0 & 1 & v_y\\0 & 0 & 1\end{bmatrix}$	→ 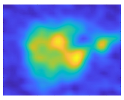 $\begin{pmatrix}v_x > 0\\v_y = 0\end{pmatrix}$
Reflection	$\begin{bmatrix}-1 & 0 & 0\\0 & 1 & 0\\0 & 0 & 1\end{bmatrix}$	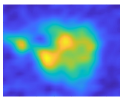
Scale	$\begin{bmatrix}c_x & 0 & 0\\0 & c_y & 0\\0 & 0 & 1\end{bmatrix}$	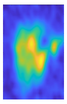 $\begin{pmatrix}c_x = 0\\c_y = 0.5\end{pmatrix}$
Rotation	$\begin{bmatrix}\cos\theta & -\sin\theta & 0\\\sin\theta & \cos\theta & 0\\0 & 0 & 1\end{bmatrix}$	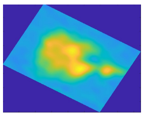 $(\theta = 30°)$
Shear	$\begin{bmatrix}1 & c_x & 0\\c_y & 1 & 0\\0 & 0 & 1\end{bmatrix}$	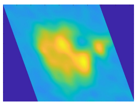 $\begin{pmatrix}c_x = 0\\c_y = 0.5\end{pmatrix}$

(B) The techniques for registration applied in this work

Registration methods		Detection targets	Specific settings
Feature-based Registration[#]	*SIFT*	Potential feature points that are invariant to scale and rotation identified by a Gaussian differentiation function	This algorithm was basically automatic.
	FAST	Corner features	Four transformations applicable: rigid, similarity, affine, and projective. In some cases, rotations were applied. Post-processing based on non-rigid method was routinely applied to optimize the registration.

	MinEigen	Corner features	Four transformations applicable: rigid, similarity, affine, and projective. In some cases, rotations were applied. Post-processing based on non-rigid method was routinely applied to optimize the registration.
	Harris	Corner features	Four transformations applicable: rigid, similarity, affine, and projective. In some cases, rotations were applied. Post-processing based on non-rigid method was routinely applied to optimize the registration.
	BRISK	Corner features	Four transformations applicable: rigid, similarity, affine, and projective. Post-processing based on non-rigid method was routinely applied to optimize the registration.
	ORB	Corners with changes in scale and/or rotation	Four transformations applicable: rigid, similarity, affine, and projective. The scale factor for image decomposition was 1.2 and the number of decomposition levels was 4. Namely the scale value at each level of decomposition was $1.2^{\alpha-1}$, where α was any value within the range [0,3]. Post-processing based on non-rigid method was routinely applied to optimize the registration.
	SURF	Blobs (supports changes in scale and rotation)	Four transformations applicable: rigid, similarity, affine, and projective. In some cases, rotations were applied. Post-processing based on non-rigid method was routinely applied to optimize the registration.
	KAZE	Multiscale blob features from a scale space constructed using nonlinear diffusion	Four transformations applicable: rigid, similarity, affine, and projective. Three methods for computing conductivity were applicable: region (select large regions, Perona and Malik conductivity coefficients were used), sharpedge (select high-contrast edges, Perona and Malik conductivity coefficients were used), and edge (instead of crossing the edge of image, both sides of an edge were smoothed, Weickert conductivity coefficient was used).
	MSER	Regions of uniform intensity	Four transformations applicable: rigid, similarity, affine, and projective. In some cases, rotations were applied. Post-processing based on non-rigid method was routinely applied to optimize the registration.
Intensity-based Registration	*Monomodal intensity*	Registers images with similar brightness and contrast that are captured on the same type of scanner or sensor	Four transformations were applicable: similarity, rigid, affine and translation. The intensity parameters were: maximum iterations were 100, minimum step length was 1.0×10^{-5}, maximum step length was 0.0625, gradient magnitude tolerance was 0.0001, relaxation factor was 0.5, number of pyramid level was 3. Post-processing based on non-rigid method was routinely applied to optimize the registration.
	Multimodal intensity	Registers images with different brightness and contrast	Four transformations were applicable: similarity, rigid, affine and translation. The normalization was pre-performed to ensure that the intensity ranges of each image pairs were the same. The Gaussian Blur was also performed as a pre-processing method. All pixels were used for transformation. The intensity parameters were: number of spatial samples was 500, number of histogram bins was 50, initial radius was 0.00625, epsilon was 1.5×10^{-6}, maximum iterations were 300,

			growth factor was 1.05, number of pyramid level was 3. Post-processing based on non-rigid method was routinely applied to optimize the registration.
	*Phase correlation**	Registers images in the frequency domain	Three transformations applicable: rigid, similarity, and translation. Windows were applied in this method. Post-processing based on non-rigid method was routinely applied to optimize the registration.
Nonrigid Registration	*Nonrigid*	Nonglobal transformations where a displacement field are generated	The number of iterations was 100 on every pyramid level. The number of Gaussian pyramid reduction levels was 3. The standard deviation of Gaussian smoothing was 1. The displacement field generated by this registration method was not applied to the final transformation since the local torching and elastic deformations that could reflect the tumor growth may be eliminated after the non-rigid transformation.

[#] The number of features detected and the quality of the matched features are selected by the registration performer on a case-by-case basis. Specifically, in ORB, only the quality of matched features could be tuned.

* The aspect ratio of each image was transformed into square to achieve better results.

When each registration is completed, the quality score based on the Structure Similarity Index Measure (SSIM) is used to evaluate the effectiveness of the registration to select the model output that achieves the best registration quality into the deep learning model for feature extraction. Generally, the closer the SSIM value is to 1, the more satisfactory the registration quality is. Different registration methods and their settings can yield similar SSIM scores but their errors could be located in various regions of the image. Therefore, we invited two radiologists (a senior radiologist with 16 years of working experience [Participant A], and a radiologist undergoing residency training [Participant B]) and two thoracic surgeons (a senior surgeon with 17 years of working experience [Participant C], and a surgeon undergoing residency training [Participant D]) to inspect the image overlay to confirm which registration technique is the most acceptable in monitoring the growth of a pulmonary nodule.

In this study, the perceptual model is used to evaluate the alignment quality, and the metric used is the SSIM value, which is in accordance with the intuitive perception of human eyes. It is a distance-based metric that examines the intuitive similarity of two images after alignment based on brightness, structure and contrast. In general, the SSIM value is defined as

$$\mathrm{SSIM}(x,y) = [l(x,y)]^{\alpha} \cdot [c(x,y)]^{\beta} \cdot [s(x,y)]^{\gamma}$$

where where $[l(x,y)]^{\alpha}$ is the Luminance term, $[c(x,y)]^{\beta}$ is the Contrast term, and $[s(x,y)]^{\gamma}$ is the Structure term, which measure three different aspects of image similarity. Among them, the luminance is obtained by averaging all the pixels, which is essentially a measure of the average grayscale of the image (in this paper, the average CT value of the image). Naturally, in the *x*, *y* dimensions, we have μ_x and μ_y:

$$\mu_x = \frac{1}{N}\sum_{i=1}^{N} x_i \, , \mu_y = \frac{1}{N}\sum_{i=1}^{N} y_i$$

Correspondingly we have the comparison function $l(x,y)$:

$$l(x,y)=\frac{2\mu_x\mu_y+C_1}{\mu_x^2+\mu_y^2+C_1}$$

The constant C_1 is set to prevent instability when the denominator is zero.

Contrast reflects different levels of luminance, i.e., the standard deviation of luminance. One in each of the *x*, *y* dimensions, defined as:

$$\sigma_x=\sqrt{\frac{1}{N-1}\sum_{i=1}^{N}(x_i-\mu_x)^2},\sigma_y=\sqrt{\frac{1}{N-1}\sum_{i=1}^{N}\left(y_i-\mu_y\right)^2}$$

Correspondingly, the covariance σ_{xy} can be expressed as

$$\sigma_{xy}=\frac{1}{N-1}\sum_{i=1}^{N}\left[(x_i-\mu_x)\cdot\left(y_i-\mu_y\right)\right]$$

These definitions lead to the complete definition of the three terms:

$$l(x,y)=\frac{2\mu_x\mu_y+C_1}{\mu_x^2+\mu_y^2+C_1},c(x,y)=\frac{2\sigma_x\sigma_y+C_2}{\sigma_x^2+\sigma_y^2+C_2},s(x,y)=\frac{\sigma_{xy}+C_3}{\sigma_x\sigma_y+C_3}$$

In this work, we set the $\alpha=\beta=\gamma=1$, and $C_3=\ C_2/2$, then the SSIM index could be simplified as:

$$\text{SSIM}(x,y)=\frac{\left(2\mu_x\mu_y+C_1\right)\cdot\left(2\sigma_{xy}+C_2\right)}{\left(\mu_x^2+\mu_y^2+C_1\right)\cdot\left(\sigma_x^2+\sigma_y^2+C_2\right)}$$

The dynamic range D of this work was set to be 255 (since the images were recorded as uint8), and we specify a three-element vector of non-negative numbers (in the form $[C_1,C_2,C_3]$) to be the regularization constants for the luminance, contrast and structure terms in order to avoid instability in image regions where the local mean or standard deviation is close to zero. Therefore, according to the general experience in registration evaluation, these constants were selected to be small, non-zero values as:

$$C_1=(0.01\cdot D)^2,C_2=(0.03\cdot D)^2,C_3=\ C_2/2$$

Intuitively, the SSIM values we compute in this way are a matrix described by the mean, standard deviation of the CT values (i.e., grayscale) of each of the two images, and the corresponding covariance between the two images. The final metric we use to measure the final registration is the average SSIM value obtained by sliding window averaging (weighted by Gaussian). In general, the closer the calculated SSIM value is to 1, the higher the quality of the registration is. However, different registration methods may produce SSIM scores with similar values, but their errors may be located in different regions of the image, so a comprehensive evaluation combined with human eye observation is still essential. At the same time, the use of this method dictates that it is impossible to compare the SSIM values in a cross-task way, since the relative sizes of the mask area and the lung tumor area are different, and the registration effect of the masked black area will always be "accurate" because the gray level of the background area is zero due to the effect of the mask.

Our results suggest that monomodal intensity appeared to be the most informative method in lung nodule

image registration in terms of SSIM values, although slightly lower than unimodal intensity alignment in terms of registration success, but these factors are influenced by the quality of the images extracted by the participants. After the transformation matrix is extracted by registration, it is applied to one of the CT images, thus eliminating the effects of body position and pose on the image differencing, and then the transformed CT information is differenced from the other baseline CT (i.e., the matrix is subtracted by elements) to obtain the Delta image, which is further fed into the deep CNN to extract the feature set.

c. Feature extraction based on CNN

We improved the AlexNet pre-trained by ImageNet to fit the learning task by replacing the last two layers with our layers, here we replaced the last two layers with a fully connected layer with ten and two nodes, respectively, and a softmax layer. The remaining parameters of the original model were retained and involved in the training as initial conditions. The features were extracted from the first fully connected layer we added into the net (detailed in following appendixes).

Note:

A

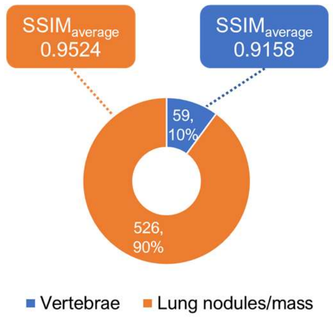

B

C/T ratio	Participants (Vertebrae/Lung lesions)			
	A	B	C	D
Pure GGO	10/130	12/128	14/126	12/128
0 < C/T ≤ 25%	2/50	2/50	1/51	3/49
25% < C/T ≤ 50%	9/78	8/79	11/76	5/82
C/T > 50%	44/262	51/255	42/264	43/263

Supplementary Figure D4 | The image masking process in this work.

We mentioned in the main text of the paper that in the 3D view selection session, we asked four participants to extract images of both the spine and nodes for registration in cases where some of the variations were too significant for the alignment algorithm to extract feature points. A comparison of the alignment methods and their results for nodules with different solid components is given above. It can be seen that for the nodules with the middle proportion of solidity, the alignment using only the information of the nodules themselves is sufficient in most cases. In contrast, for some of the ground glass nodules and solid nodules, it may be necessary to use the information of the spine to obtain the affine transformation.

Appendix E | The consistency of lesion segmentation

For the lesion segmentation, the four operators did all cases. In the main text of the manuscript, we chose the set of images with the relatively highest SSIM values among segmentation results from operators A, B, C, and D to enter the subsequent feature extraction. By comparing the SSIM values and final model accuracies for each operator, we measured the magnitude of the impact that the segmentation results of different operators caused on the results. In **Supplementary Figure E**, the final SSIM values obtained for various combinations of operators and engineers are given in **Supplementary Figure E-A** and **Supplementary Figure E-B**. It can be seen that the magnitude of the SSIM values was mainly determined by the registration method. Multimodal intensity is the relatively best registration method as it has the highest SSIM without sacrificing the success rate of registration **(Supplementary Figure E-A, Supplementary Figure E-B, Supplementary Table E)**. On the classical deep learning (DL) modality, there is no significant AUC difference between operators **(Supplementary Figure E-D and Supplementary Figure E-F)**. On the delta-DL and hybrid DL, there does seem to be some variation between different combinations of operators and engineers **(Supplementary Figures E-C, E, G, and H)**. However, the magnitude of these differences was not large (coefficients of variation of AUC values among four observers: $\text{CV-AUC}_{\text{delta-DL-}\alpha} = 0.0094$, $\text{CV-AUC}_{\text{delta-DL-}\beta} = 0.0074$, $\text{CV-AUC}_{\text{hybrid-DL-}\alpha} = 0.0053$, $\text{CV-AUC}_{\text{hybrid-DL-}\beta} = 0.0098$, α and β denote two engineers respectively). As for the rate of successful registration and the compositions of image pairs selected by engineers from four operators/participants, there were no statistically significant differences between the different combinations of operators and engineers (the registration method was multimodal intensity, **Supplementary Figure E-J**), and the proportions of image pairs from the four operators selected by the different engineers were similar (**Supplementary Figures E-K and L**).

A

Techniques	Rate of success (Completed registration with SSIM score > 0.75)		Average SSIM scores (in the successful cases)		Participant's average evaluation (Normalized) (*α* and *β* represent two engineers with experience in image registration)							
					Participant A		Participant B		Participant C		Participant D	
	α	*β*	*α*	*β*	*α*	*β*	*α*	*β*	*α*	*β*	*α*	*β*
SIFT	**43.28%**	**43.28%**	0.9042	0.9042	-0.375	-0.109	-0.095	-0.324	-0.202	-0.383	-0.274	-0.298
FAST	93.77%	92.98%	0.8788	0.8781	**-1.000**	-0.831	-0.830	-0.940	-0.707	-0.789	-0.769	-0.807
MinEigen	92.43%	92.10%	**0.8695**	**0.8702**	-0.972	**-1.000**	**-1.000**	**-1.000**	**-1.000**	**-1.000**	**-1.000**	**-1.000**
Harris	96.65%	96.85%	0.9189	0.9183	-0.061	0.085	0.077	0.051	0.167	-0.136	0.089	0.044
BRISK	99.02%	99.02%	0.9480	0.9460	0.608	0.718	0.715	0.711	0.584	0.642	0.669	0.688
ORB	98.77%	98.77%	0.9382	0.9402	0.423	0.553	0.446	0.558	0.474	0.576	0.661	0.578
SURF	98.77%	98.77%	0.9560	0.9555	***1.000***	0.959	0.974	0.937	0.856	***1.000***	***1.000***	0.938
KAZE	97.24%	97.15%	0.9463	0.9453	0.678	0.603	0.603	0.648	0.860	0.812	0.710	0.661
MSER	97.50%	97.86%	0.9322	0.9310	0.375	0.323	0.444	0.403	0.423	0.447	0.402	0.390
Monomodal intensity	***99.14%***	***99.14%***	0.9521	0.9498	0.663	0.838	***1.000***	0.755	0.833	0.900	0.759	0.828
Multimodal intensity	98.78%	98.86%	***0.9587***	***0.9580***	0.904	***1.000***	0.956	***1.000***	***1.000***	0.993	0.902	***1.000***
*Phase correlation**	97.55%	97.40%	0.9370	0.9288	0.225	0.291	0.289	0.250	0.237	0.594	0.451	0.280

B

Techniques	**SSIM** (Form: mean ± SD)			
	Participant A	Participant B	Participant C	Participant D

	α	β	α	β	α	β	α	β
SIFT	0.904 ± 0.035	0.904 ± 0.035	0.904 ± 0.026	0.904 ± 0.026	0.904 ± 0.038	0.904 ± 0.038	0.904 ± 0.020	0.904 ± 0.037
FAST	0.879 ± 0.024	0.885 ± 0.033	0.886 ± 0.011	0.880 ± 0.034	0.889 ± 0.022	0.878 ± 0.027	0.887 ± 0.031	0.895 ± 0.012
MinEigen	0.880 ± 0.033	0.878 ± 0.029	0.879 ± 0.023	0.878 ± 0.003	0.878 ± 0.029	0.868 ± 0.023	0.879 ± 0.011	0.888 ± 0.023
Harris	0.916 ± 0.021	0.921 ± 0.009	0.922 ± 0.006	0.920 ± 0.024	0.923 ± 0.035	0.908 ± 0.026	0.918 ± 0.018	0.925 ± 0.023
BRISK	0.943 ± 0.031	0.947 ± 0.035	0.948 ± 0.031	0.946 ± 0.027	0.939 ± 0.002	0.944 ± 0.033	0.939 ± 0.021	0.947 ± 0.030
ORB	0.936 ± 0.025	0.940 ± 0.027	0.937 ± 0.029	0.940 ± 0.018	0.935 ± 0.026	0.941 ± 0.016	0.939 ± 0.005	0.943 ± 0.009
SURF	0.959 ± 0.028	0.956 ± 0.007	0.959 ± 0.037	0.955 ± 0.017	0.949 ± 0.019	0.960 ± 0.032	0.951 ± 0.038	0.956 ± 0.023
KAZE	0.946 ± 0.020	0.942 ± 0.014	0.944 ± 0.006	0.944 ± 0.016	0.950 ± 0.024	0.951 ± 0.012	0.941 ± 0.016	0.946 ± 0.023
MSER	0.934 ± 0.033	0.931 ± 0.023	0.937 ± 0.007	0.934 ± 0.024	0.933 ± 0.036	0.935 ± 0.015	0.930 ± 0.019	0.937 ± 0.027
Monomodal intensity	0.945 ± 0.020	0.952 ± 0.020	0.960 ± 0.021	0.948 ± 0.022	0.949 ± 0.024	0.955 ± 0.010	0.942 ± 0.017	0.952 ± 0.022
Multimodal intensity	0.955 ± 0.007	0.958 ± 0.040	0.958 ± 0.029	0.958 ± 0.006	0.955 ± 0.019	0.960 ± 0.019	0.947 ± 0.019	0.958 ± 0.032
Phase correlation	0.928 ± 0.006	0.930 ± 0.031	0.931 ± 0.023	0.928 ± 0.014	0.926 ± 0.019	0.941 ± 0.029	0.931 ± 0.019	0.933 ± 0.017

C

**Techniques**	**AUC (Delta-DL modality), *n* = 40**							
	Participant A		Participant B		Participant C		Participant D	
	α	β	α	β	α	β	α	β
Multimodal intensity	0.802 ± 0.012	0.814 ± 0.015	0.820 ± 0.031	0.812 ± 0.019	0.817 ± 0.027	0.799 ± 0.035	0.805 ± 0.011	0.807± 0.020

* Please be informed that here the repeated experiments refer to the setting that the modified AlexNet was fed by the input dataset (fixed with the same operator and engineer combination, for example, the image pairs by operator A and registered by Engineer α) repeatedly, instead of the repeated delineation processes.

D

**Techniques**	**AUC (Pure-DL modality), *n* = 40**							
	Participant A		Participant B		Participant C		Participant D	
	α	β	α	β	α	β	α	β
Multimodal intensity	0.787 ± 0.018	0.787 ± 0.018	0.784 ± 0.038	0.784 ± 0.038	0.774 ± 0.037	0.774 ± 0.037	0.789 ± 0.025	0.789 ± 0.025

* Please be informed that here the repeated experiments refer to the setting that the modified AlexNet was fed by the input dataset (fixed with the same operator and engineer combination, for example, the image pairs by operator A and registered by Engineer α) repeatedly, instead of the repeated delineation processes.

** Please note that the Engineers were not participated in this procedure since there were no need of registration in the Pure-DL modality. Therefore, the test results were the same for a specific participant/operator.

E

**Techniques**	**AUC (Pure-DL + Delta-DL modality), *n* = 40**							
	Participant A		Participant B		Participant C		Participant D	
	α	β	α	β	α	β	α	β
Multimodal intensity	0.816 ± 0.017	0.819 ± 0.019	0.826 ± 0.018	0.832 ± 0.021	0.825 ± 0.027	0.810 ± 0.031	0.827± 0.016	0.825 ± 0.022

F

**Independent two-sample t-test of AUC (Pure-DL modality) *n* = 40***		**Participant A**		**Participant B**		**Participant C**		**Participant D**	
		α	β	α	β	α	β	α	β
**Participant A**	α**								
	β**								
**Participant B**	α	0.2138	0.2138						

	β	0.2138	0.2138						
Participant C	α	0.0743	0.0743	0.9913	0.9913				
	β	0.0743	0.0743	0.9913	0.9913				
Participant D	α	0.9818	0.9818	0.3480	0.3480	0.0574	0.0574		
	β	0.9818	0.9818	0.3480	0.3480	0.0574	0.0574		

* Please be informed that here the repeated experiments refer to the setting that the modified AlexNet was fed by the input dataset (fixed with the same operator and engineer combination, for example, the image pairs by operator A and registered by Engineer α) repeatedly, instead of the repeated delineation processes.

** Please note that the Engineers were not participated in this procedure since there were no need of registration in the Pure-DL modality. Therefore, the test results were the same for a specific participant/operator.

G

Independent two-sample t-test of AUC (Delta-DL modality) *n* = 40*		Participant A		Participant B		Participant C		Participant D	
		α	β	α	β	α	β	α	β
Participant A	α								
	β	***							
Participant B	α	0.0094**	0.2430						
	β	***	0.1069	0.1702					
Participant C	α	0.0127*	0.3236	0.5405	0.7062				
	β	0.8805	0.0104*	0.3014	0.0329*	0.0227*			
Participant D	α	0.0083**	0.0776	0.0511	0.1069	0.0014**	0.3513		
	β	0.1376	0.3728	0.0346*	0.1113	0.0171*	0.0258*	0.9200	

* Please be informed that here the repeated experiments refer to the setting that the modified AlexNet was fed by the input dataset (fixed with the same operator and engineer combination, for example, the image pairs by operator A and registered by Engineer α) repeatedly, instead of the repeated delineation processes.

H

Independent two-sample t-test of AUC (Pure-DL + Delta-DL modality) *n* = 40*		Participant A		Participant B		Participant C		Participant D	
		α	β	α	β	α	β	α	β
Participant A	α								
	β	0.1795							
Participant B	α	0.0051**	0.1523						
	β	***	0.0022**	0.0282*					
Participant C	α	0.0545	0.0229*	0.5003	0.5691				
	β	0.3614	0.1450	0.4362	0.1287	0.0660			
Participant D	α	***	0.0557	0.3139	0.9691	0.2026	0.0142*		
	β	***	0.1000	0.4432	0.2284	0.9651	0.1347	0.4129	

* Please be informed that here the repeated experiments refer to the setting that the modified AlexNet was fed by the input dataset (fixed with the same operator and engineer combination, for example, the image pairs by operator A and registered by Engineer α) repeatedly, instead of the repeated delineation processes.

I

Techniques	Rate of success							
	Participant A		Participant B		Participant C		Participant D	
	α	β	α	β	α	β	α	β
SIFT	43.5%	43.5%	43.2%	43.2%	42.2%	42.2%	44.3%	44.3%
FAST	93.3%	92.2%	92.5%	91.2%	93.9%	94.1%	95.5%	94.5%
MinEigen	91.2%	92.5%	92.6%	91.6%	94.5%	92.1%	91.5%	92.1%

Harris	96.7%	96.8%	96.5%	97.5%	95.9%	95.2%	97.5%	97.9%
BRISK	98.7%	98.9%	98.9%	99.3%	99.3%	99.1%	99.1%	98.7%
ORB	98.8%	98.7%	98.9%	99.2%	99.0%	98.9%	98.5%	98.4%
SURF	98.8%	98.2%	98.7%	99.1%	98.5%	98.7%	98.9%	98.8%
KAZE	97.5%	97.2%	97.3%	96.7%	96.9%	97.4%	97.1%	97.5%
MSER	97.7%	97.9%	97.8%	97.9%	96.9%	97.5%	97.6%	98.3%
Monomodal intensity	99.1%	98.7%	99.3%	99.2%	99.1%	99.2%	98.9%	99.3%
Multimodal intensity	98.9%	98.9%	99.1%	98.9%	98.1%	98.8%	99.1%	98.9%
Phase correlation	97.1%	97.5%	96.9%	98.0%	98.0%	96.8%	98.4%	97.3%

J

Rate of success for registrations based on multimodal intensity (χ^2 test)		Operator A		Operator B		Operator C		Operator D	
		Engineer α	Engineer β	Engineer α	Engineer β	Engineer α	Engineer β	Engineer α	Engineer β
Operator A	Engineer α								
	Engineer β	0.7880							
Operator B	Engineer α	0.7803	0.7803						
	Engineer β	0.7880	0.7880	1.0000					
Operator C	Engineer α	0.4761	0.4761	0.3284	0.4761				
	Engineer β	0.7880	0.7880	1.0000	0.7880	0.4761			
Operator D	Engineer α	0.7717	0.7717	1.0000	0.7717	0.2082	0.7717		
	Engineer β	1.0000	1.0000	0.7717	1.0000	0.3284	1.0000	1.0000	

K

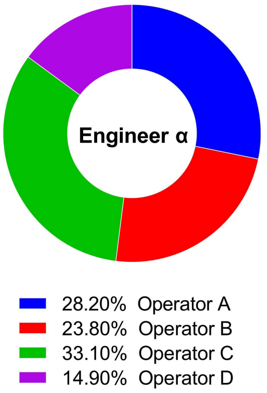

L

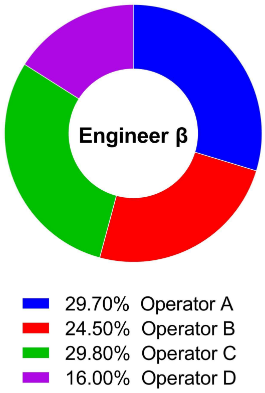

Supplementary Figure E | The consistency of four operators (A, B, C, and D) for the lesion segmentation. A. SSIM scores of various registration methods. **B.** SSIM scores (in form of mean ± SD) of four operators (A, B, C, and D) and two engineers α and β (in specific). **C.** AUC values of delta-deep learning (DL) modality with image pair aligned by multimodal intensity. **D.** AUC values of pure-DL modality with image pair aligned by multimodal intensity. **E.** AUC values of delta-DL and pure-DL modalities with image pair aligned by multimodal intensity. **F.** Independent two-sample t-test of AUC (Pure-DL modality), n = 40. **G.** Independent two-sample t-test of AUC (delta-DL modality), n = 40. **H.** Independent two-sample t-test of AUC (pure-DL and delta-DL modality), n = 40. **I.** Rate of success in image pair registration. **J.** Rate of success for registrations based on multimodal intensity (χ^2 test). **K.** The final composition of image pairs selected by engineer α. **L.** The final composition of image pairs selected by engineer β. The image pairs entering the final model were determined by a discussion in engineers.

Table E | Comparison between the average registration SSIM values and rate of successful registration (SSIM ≥

0.75) of image pairs from four operators

(a) **For Engineer A** (* < 0.05, **p < 0.01, ***p < 0.001, comparison between the average SSIM values of image pairs from four operators; Red unit, the row is superior than the column; Green unit, the column is superior than the row)

Pairs	SIFT	FAST	MiniEigin	Harris	BRISK	ORB	SURF	KAZE	MSER	Mono-MI	Multi-MI	PC
SIFT	n.a.											
FAST	**	n.a.										
MiniEigen	***	0.5363	n.a.									
Harris	0.0949	***	***	n.a.								
BRISK	***	***	***	***	n.a.							
ORB	***	***	***	*	*	n.a.						
SURF	***	***	***	***	0.4256	*~** (0.0010)	n.a.					
KAZE	***	***	***	***	*	**	0.5712	n.a.				
MSER	***	***	***	0.7770	***	0.4881	*	**	n.a.			
Mono-MI	***	***	***	***	0.0566	*	0.6093	0.6622	***	n.a.		
Multi-MI	***	***	***	***	0.6637	***	0.2199	0.4638	*	**	n.a.	
PC	***	***	***	0.5325	***	0.4287	***	**	**	***	***	n.a.

(b) **For Engineer B** (* < 0.05, **p < 0.01, ***p < 0.001, comparison between the average SSIM values of image pairs from four operators; Red unit, the row is superior than the column; Green unit, the column is superior than the row)

Pairs	SIFT	FAST	MiniEigin	Harris	BRISK	ORB	SURF	KAZE	MSER	Mono-MI	Multi-MI	PC
SIFT	n.a.											
FAST	***	n.a.										
MiniEigen	***	**	n.a.									
Harris	***	***	***	n.a.								
BRISK	***	***	***	***	n.a.							
ORB	***	***	***	***	*	n.a.						
SURF	***	***	***	***	0.3113	***	n.a.					
KAZE	***	***	***	***	0.1171	0.0615	*	n.a.				
MSER	***	***	***	0.1477	**	***	**	0.0515	n.a.			
Mono-MI	***	***	***	***	0.8902	0.6042	***	**	***	n.a.		
Multi-MI	***	***	***	***	0.1296	*	0.6283	*	***	0.1509	n.a.	
PC	0.0791	***	***	0.1836	***	***	***	*	***	***	***	n.a.

(c) **For Engineer A** (* < 0.05, **p < 0.01, ***p < 0.001, comparison between the rate of successful registration; Red unit, the row is superior than the column; Green unit, the column is superior than the row)

Pairs	SIFT	FAST	MiniEigin	Harris	BRISK	ORB	SURF	KAZE	MSER	Mono-MI	Multi-MI	PC
SIFT	n.a.											

FAST	***	n.a.										
MiniEigen	***	0.4175	n.a.									
Harris	***	*	***	n.a.								
BRISK	***	***	***	**	n.a.							
ORB	***	***	***	*	1.0000	n.a.						
SURF	***	***	***	*	1.0000	0.7880	n.a.					
KAZE	***	**	***	0.6115	0.0527	0.0920	0.0920	n.a.				
MSER	***	**	***	0.4924	0.0781	0.1319	0.1319	1.0000	n.a.			
Mono-MI	***	***	***	**	1.0000	0.7718	0.7717	*	*	n.a.		
Multi-MI	***	**	***	*	1.0000	0.7880	0.7880	0.0920	0.1319	0.7717	n.a.	
PC	***	***	***	0.3842	0.1144	0.1864	0.1864	0.8533	1.0000	0.0643	0.1864	n.a.

(d) **For Engineer B** (* < 0.05, **p < 0.01, ***p < 0.001, comparison between the rate of successful registration; Red unit, the row is superior than the column; Green unit, the column is superior than the row)

Pairs	SIFT	FAST	MiniEigin	Harris	BRISK	ORB	SURF	KAZE	MSER	Mono-MI	Multi-MI	PC
SIFT	n.a.											
FAST	***	n.a.										
MiniEigen	***	0.6558	n.a.									
Harris	***	***	***	n.a.								
BRISK	***	***	***	*	n.a.							
ORB	***	***	***	*	1.0000	n.a.						
SURF	***	***	***	*	1.0000	0.7880	n.a.					
KAZE	***	***	***	1.0000	*	0.0634	0.0634	n.a.				
MSER	***	***	***	0.4665	0.1652	0.2594	0.2594	0.5789	n.a.			
Mono-MI	***	***	***	*	1.0000	0.7717	0.7717	*	0.0964	n.a.		
Multi-MI	***	***	***	*	1.0000	0.7880	0.7880	0.0634	0.2594	0.7717	n.a.	
PC	***	***	***	0.7239	0.0781	0.1319	0.1319	0.8577	0.8483	*	0.1319	n.a.

Abbreviations: Mono-MI, monomodal intensity; Multi-MI, multi-modal intensity; PC, phase correlation.

Appendix F | Working arrangement

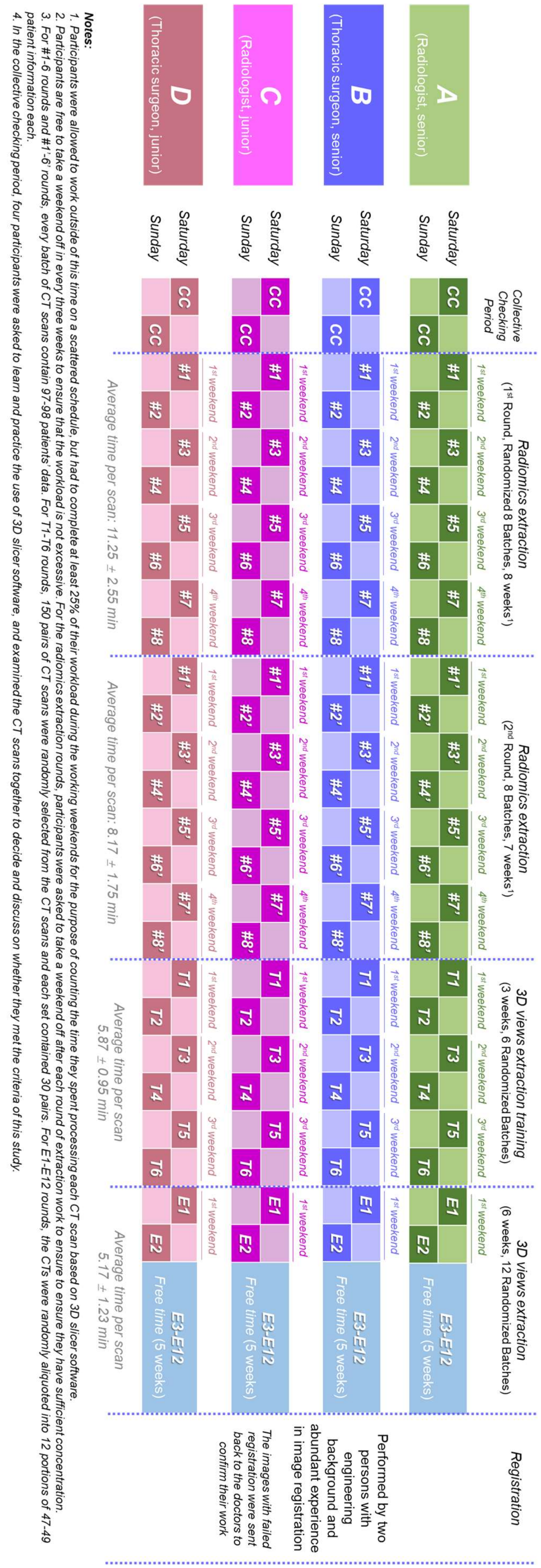

Supplementary Figure F1 | Working arrangement of CT image segmentation and 3D image extraction (placed

vertically).

We invited four doctors with related specialties to participate in ROI segmentation and 3D image extraction of nodules (**Participant A** is a radiologist who has worked in radiology for 16 years; **Participant B** is a surgeon who has worked in thoracic surgery for 14 years; **Participant C** is a resident who participated in residency training of radiology; **Participant D** is a resident who participated in residency training of thoracic surgery). Four participants worked together according to the following arrangement (see **Supplementary Figure F1**).

First, in the first week, the participants were asked to receive training on VOI delineation with the 3D slicer. At the same time, initial screening of CTs that did not meet the requirements was also performed.

Second, the segmentation of 585 pairs of CT images in four weeks started from the second week, in which the doctors were asked to focus on segmentation tasks at the weekends (they were asked to finish at least 25% of the tasks for that week on the weekends together, where the engineer recorded the time that they spent on each scan). The time spent by the four participants on nodal/mass segmentation was recorded and counted, which was aimed to measure the expected work intensity in future practical application scenarios. During the workday, four doctors were allowed to independently and autonomously delineate the nodal and perineural contours on the remaining CT images that were not processed during the weekend hours. They were allowed a one-week break at the end of each weekly delineation task. Statistically, four doctors spent an average of 11.25 ± 2.55 minutes per image during the first round of delineation work. This round took 8 weeks.

Third, in the next 4 weeks, for the calculation of ICC values within the group, the participants were asked to process the CT scans again with the same methods and settings in the second step. Statistically, in the second round of delineation work, the physicians spent an average of 8.17 ± 1.75 minutes on each image, with a significant speed-up from the previous round. The round took 7 weeks.

Fourth, the next 3 weeks were spent on training the participants to extract the 3D images (namely the *X* view, the *Y* view, and the *Z* view) for further registration. The four participants were asked to complete the 3D image extraction task every weekend under the guidance of the registration engineers. Two engineers with registration experience and medical image processing knowledges would align the image pairs provided by the four physicians and observe the registration effect for 3 rounds of training.

Fifth, in the last 6 weeks, a 3D image extraction test was conducted at the end of the first week, and two engineers aligned the images provided by the four doctors and recorded the alignment effect with SSIM scores. They were asked to determine whether the registration effect was satisfactory and discussed the results of registration and subtraction with four doctors together. Once an agreement was reached on the actionable registration methods and subtraction effect on the description of tumor growth and intertumoral changes, the subsequent 5 weeks were free for the four doctors to work on the extraction of 3D images.

Sixth, the engineers would align and subtract the image pairs to acquire the final subtracted “images” and input them into CNNs to extraction DL-based delta-features.

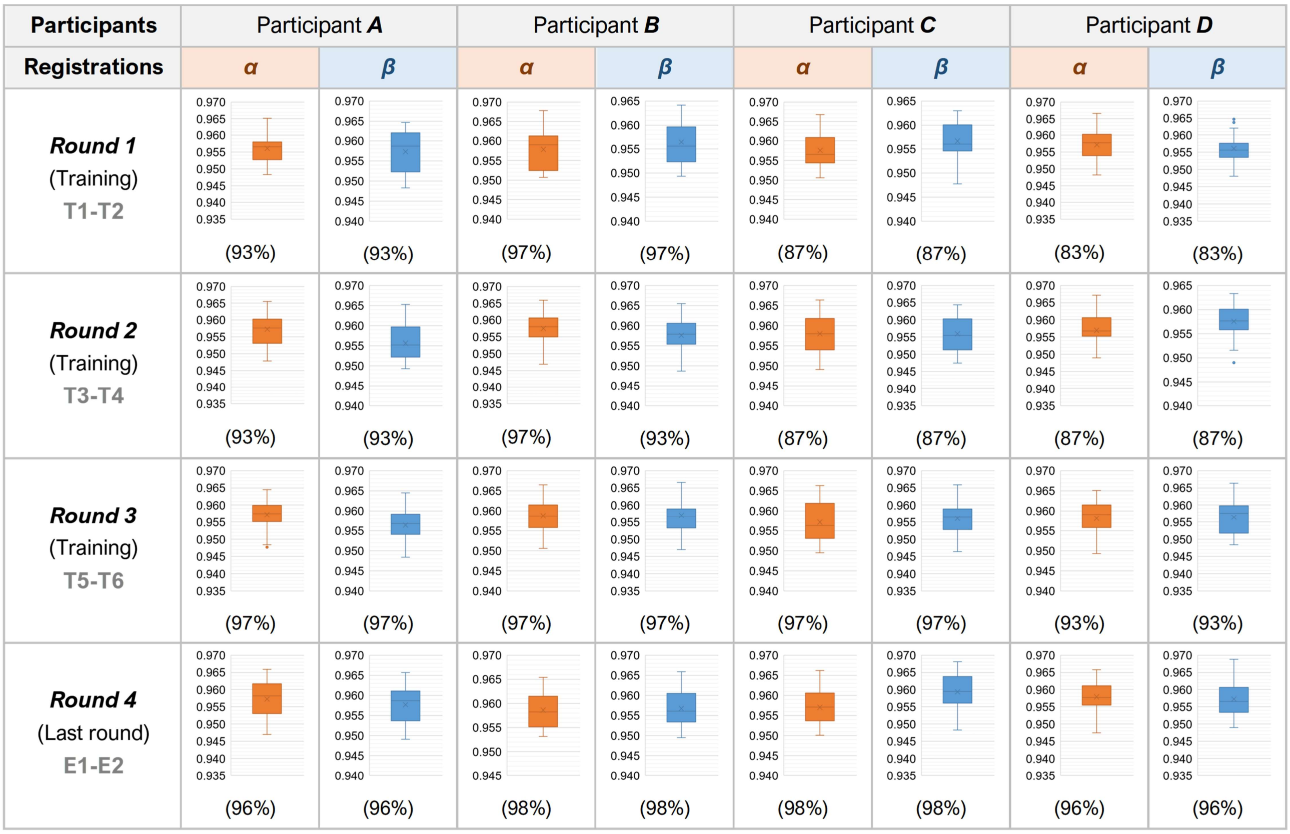

Supplementary Figure F2 | SSIM scores of the four participants' registration results by two engineers (during 1st to 3rd training rounds and during the final round)

Supplementary Figure F2 shows the SSIM scores of the four participants in training rounds 1-3 (where three randomly selected small-batch datasets were used to assess the improvement in registration quality with increasing proficiency) before the formal work, and it can be seen that the quality of the images extracted by two registration participants A and B has improved through their training processes.

Appendix G | Architecture of the deep network

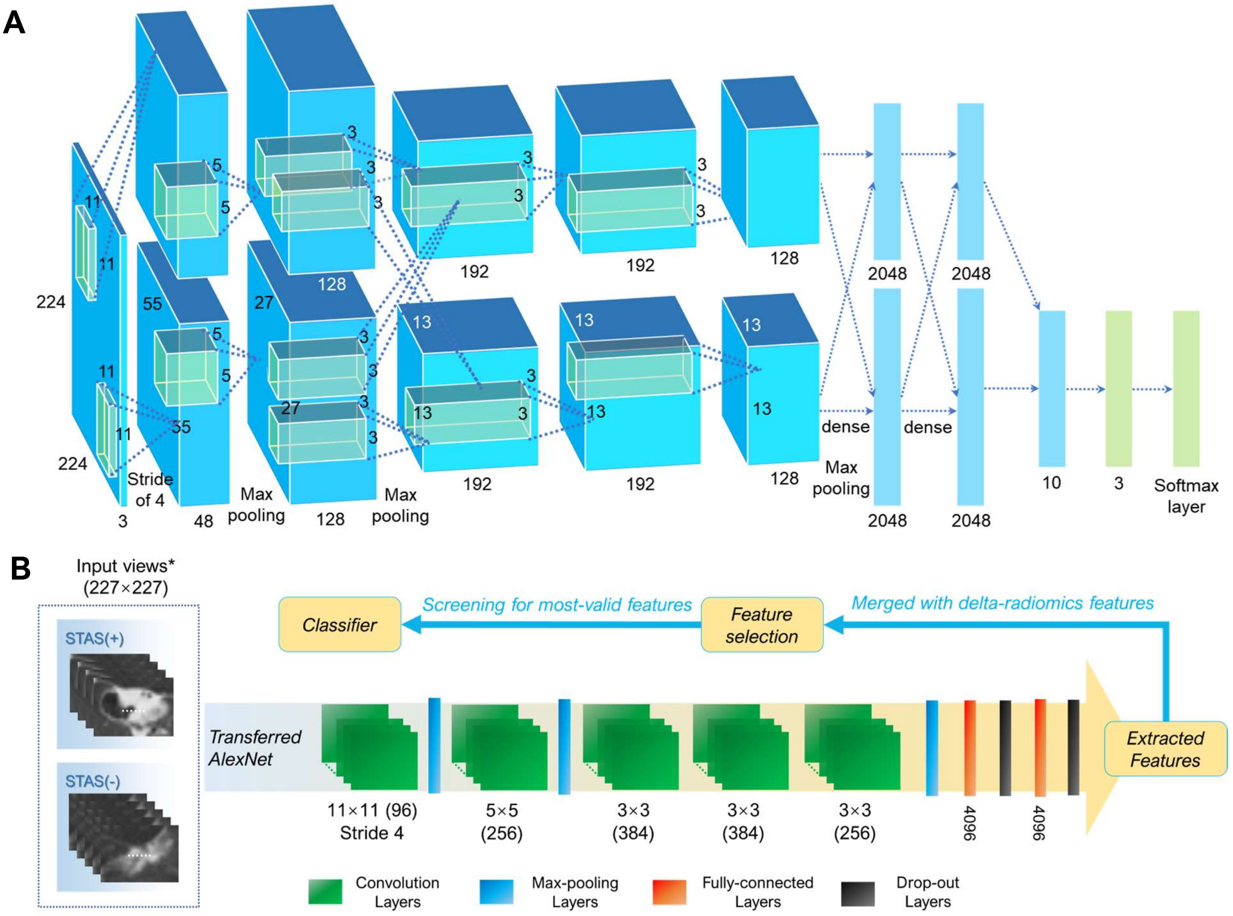

* The delta-images acquired from registration and difference could also be inputs of the AlexNet-based deep-learning feature extraction model in some cases.

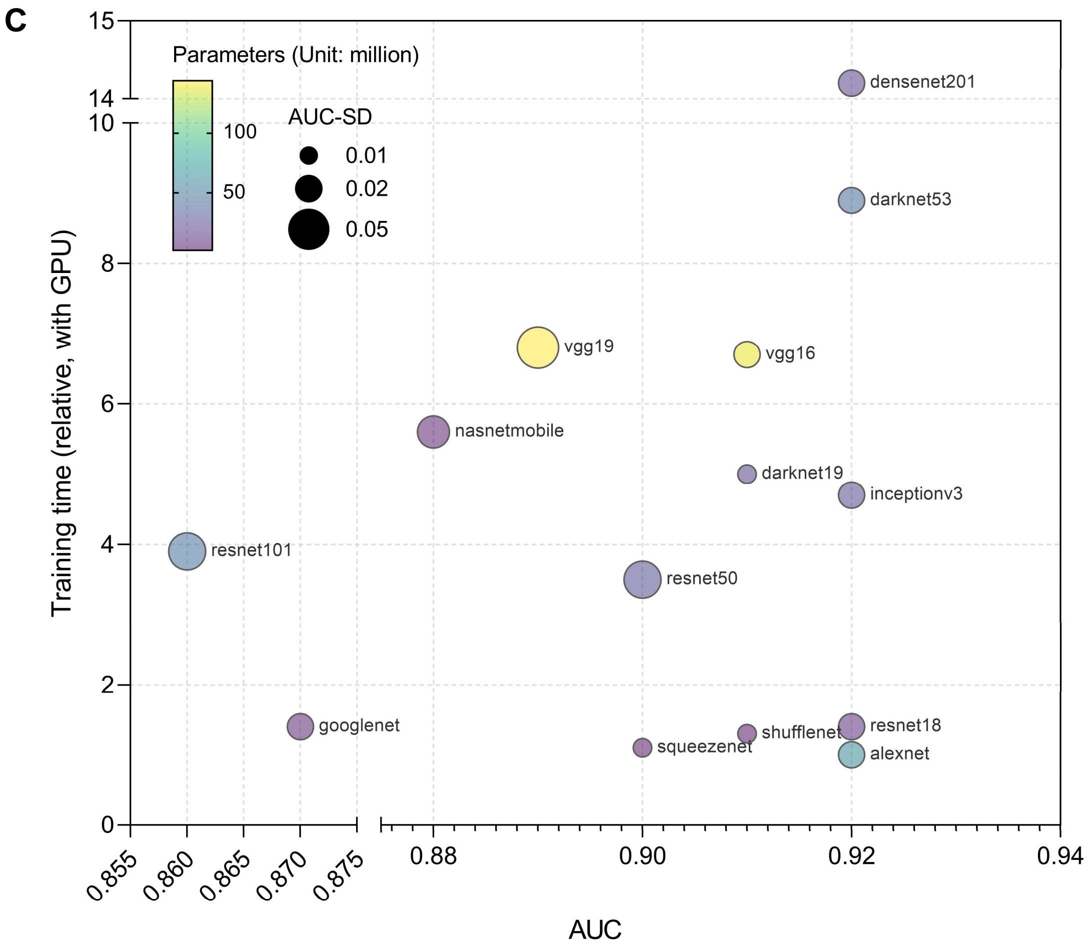

Supplementary Figure G1 | A. The structure of AlexNet. **B.** The feature extraction process based on AlexNet. **C.** The training times, AUC values (with SD values), and the number of parameters of considered CNNs.

For feature extraction, all images were input to AlexNet and the outputs of a certain layer of the network were considered as feature vectors of the image and used for classification. Since computer-aided diagnosis (CAD) systems and other medical image interpretation systems usually cannot train CNNs from scratch, common parameters can be migrated from trained CNNs and used as input classifiers for imaging tasks in transfer learning.

As a large network with 60 million parameters and 650,000 neurons (Structure shown in the **Supplementary Figure G1-A**), AlexNet requires a large number of labeled samples for training, a requirement that clinical imaging resources are often unable to meet. In this case, Transfer Learning (TL) is a convenient and effective approach that is widely used to train deep neural networks when available labeled samples are insufficient. In this approach, pre-existing parameters from pre-trained networks are used as initial parameters, which can effectively utilize these basic parameters already learned from large datasets. AlexNet used in this paper is pre-trained by ImageNet, and the parameters obtained through pre-training can help the model converge successfully during the training process. In addition, training deep networks usually requires high performance GPUs and CPUs, but TL can make the computational resource requirements relatively low, and training can be performed on a personal computer configuration.

In the model proposed in this paper, we improve AlexNet to fit the learning task by replacing the last two layers (the original model includes a fully connected layer with 1000 neurons and a softmax layer) with our layers (shown

in **Supplementary Figure G1-B**), i.e., two fully connected layers with ten and X nodes, respectively (X differs according to the specific classification task, corresponding to X target classes, e.g., corresponding to four pathological stages AAH/AIS/MIA/IAC, then X is taken as 4), and a softmax layer. The remaining parameters of the original model were retained and involved in the training as initial conditions. The entire model structure is divided into two parts: the pre-training network and the transferred network. The parameters in the pre-training network have been trained on ImageNet with millions of images and the extracted features have been shown to be effective for classification, and these parameters may only need to be slightly adjusted to fit the new images. The parameters in the migration network represent only a small fraction of the entire network, which is suitable for training on small datasets.

Actually, we considered 14 different models for feature extraction (**Supplementary Figure G1-C**). AlexNet was finally selected for feature extraction since it had the most balanced performance in accuracy, stability, and training portability. For further details how we determine the best candidate model, please refer to the following note.

Supplementary Table G1 | Essential parameters used in AlexNet training

**Name of parameter**	**Essential parameters**
Momentum	0.9
Initial Learn Rate	0.0005
Learn Rate Schedule	None
Learn Rate Drop Factor	0.1
Learn Rate Drop Period	10
L2Regularization	0.0001
Gradient Threshold Method	l2norm
Gradient Threshold	Inf
Max Epochs	60
Mini Batch Size	32
Verbose	1
Verbose Frequency	50

Note: Determining the CNN model with best performance

We chose to compare 14 networks (SqueezeNet, GoogleNet, InceptionV3, DenseNet201, ResNet18, ResNet50, ResNet101, ShuffleNet, ResNetMobile, DarkNet19, DarkNet53, AlexNet, VGG16, and VGG19) with a relatively appropriate number of parameters and a problem size more appropriate to the characteristics of the sample in this study. Modeled after AlexNet, continuous innovations in CNN networks have been achieved by deepening the network structure (e.g., VGG16/VGG19) or enhancing the functionality of the convolutional module (e.g., GoogleNet/InceptionV3). They all have their representative typical structures that make them appropriate for specific applications. We defined three performance indexes (PI_{CNN}) to evaluate and compare the performance of these CNN models (**Supplementary Tabel G2**):

Supplementary Tabel G2 | Three different definitions of performance indexes (PI) and their considerations

No.	*Definitions*	*Considerations*
1	$PI_{\text{CNN-1}} = \dfrac{AUC_{\text{average}}/AUC_{\text{average-max}}}{t/t_{\text{min}}}$	■ Model accuracy<br>■ Training time (Time complexity of the model)
2	$PI_{\text{CNN-2}} = \dfrac{AUC_{\text{average}}/AUC_{\text{average-max}}}{(t/t_{\text{min}}) \cdot \left(\dfrac{SD_{\text{AUC}}/AUC_{\text{average}}}{SD_{\text{AUC-min}}/AUC_{\text{average-max}}}\right)}$	■ Model accuracy<br>■ Training time (Time complexity of the model)<br>■ Batch stability of the model
3	$PI_{\text{CNN-3}} = \dfrac{AUC_{\text{average}}/AUC_{\text{average-max}}}{(t/t_{\text{min}}) \cdot \left(\dfrac{SD_{\text{AUC}}/AUC_{\text{average}}}{SD_{\text{AUC-min}}/AUC_{\text{average-max}}}\right)} \cdot \left(\dfrac{N_{\text{parameters-min}}}{N_{\text{parameters}}}\right)$	■ Training space (Physical storage space needed to train the model; Space complexity of the model)<br>■ Model accuracy<br>■ Training time (Time complexity of the model)<br>■ Batch stability of the model

where the AUC_{average} denotes the average value of AUCs in the repeated independent experiments (*n* = 40), SD_{AUC} is the standard deviation of AUC values (The $SD_{\text{AUC}}/AUC_{\text{average}}$ value is actually the coefficient of variation in statistics), t refers to the average time in training the CNNs, and $N_{\text{parameters}}$ is the number of parameters in a certain CNN model. $SD_{\text{AUC-min}}$, $AUC_{\text{average-max}}$, $N_{\text{parameters-min}}$,and t_{min} were introduced to nondimensionalize the parameters. When the *PI* values of the models are comparable, the models are preferred in the following order: AUC > training time > SD/AUC ＞Number of parameters. The purpose of defining the PI_{CNN} values is to select models that achieve a more stable and accurate classification with relatively little time and space cost. In our model presented in the main text, we chose the simplest definition of PI_{CNN}, namely the $PI_{\text{CNN-1}}$ to be the performance metric for model selection, where we mainly consider the model accuracy and training time of the model. It can be seen from the **Supplementary Figure G2** that the AlexNet had the biggest $PI_{\text{CNN-1}}$ among the candidate CNN models, by which we selected AlexNet to be the final CNN element/component of our optimized model. Indeed, when the batch stability of the model and the training space were considered, this conclusion could change from AlexNet as the optimized model to the SqueezeNet as the model with the best comprehensive performance.

A

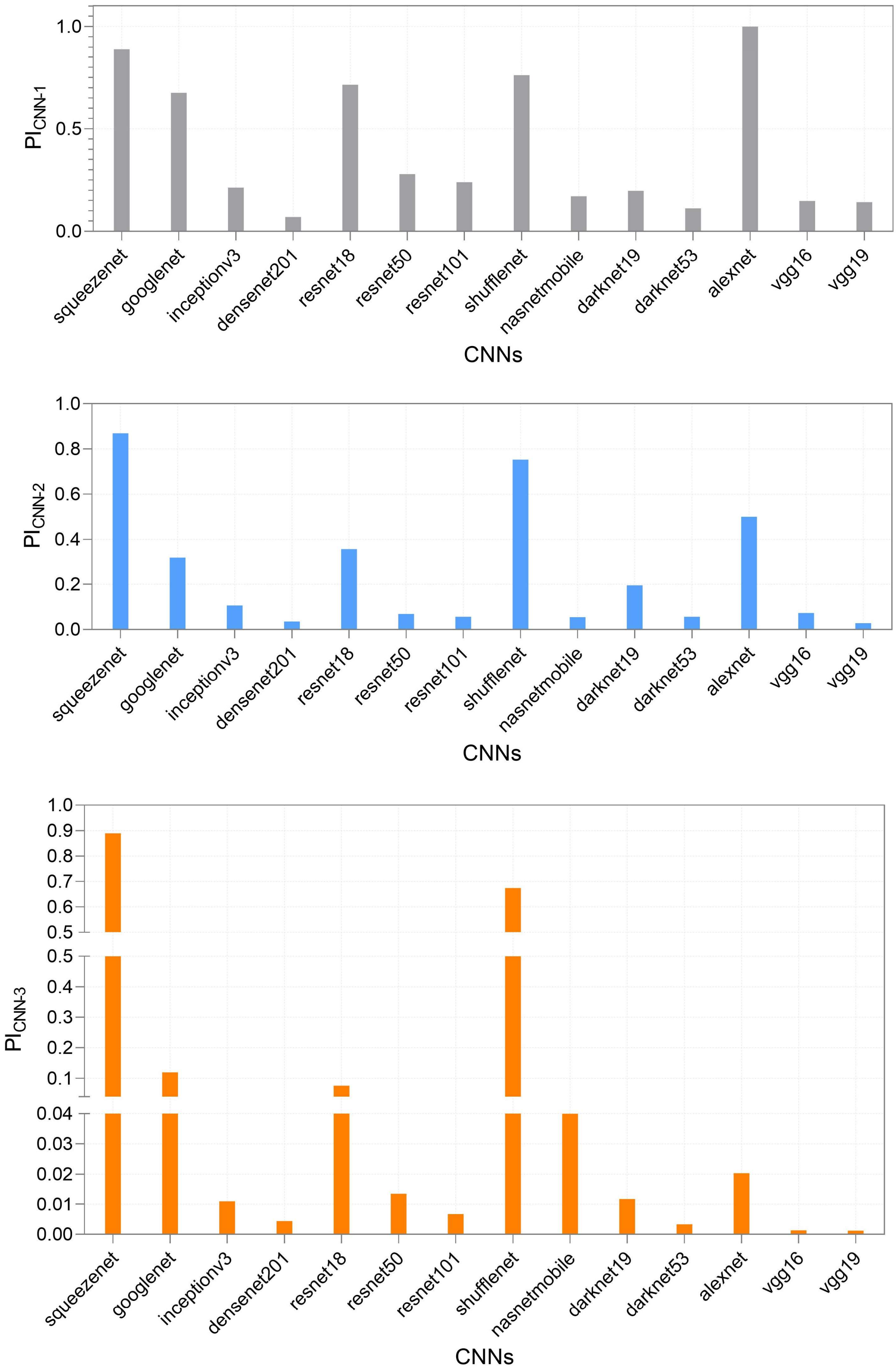

PI_{CNN-1}
CNNs
PI_{CNN-2}
CNNs
PI_{CNN-3}
CNNs
squeezenet
googlenet
inceptionv3
densenet201
resnet18
resnet50
resnet101
shufflenet
nasnetmobile
darknet19
darknet53
alexnet
vgg16
vgg19

B

Independent two-sample t-test of AUC, n = 40 (*$p < 0.05$, **$p < 0.01$, ***$p < 0.001$), Red unit, the row is superior than the column; Green unit, the column is superior than the row

CNNs	VGG19	VGG16	DenseNet201	DarkNet53	NasNetMobile	DarkNet19	Inception v3	**AlexNet**	SqueezeNet	ResNet101	ResNet50	ResNet18	GoogleNet	ShuffleNet
VGG19														
VGG16	***													
DenseNet201	***	***												
DarkNet53	***	***	0.3005											
NasNetMobile	0.3204	***	***	***										
DarkNet19	***	0.1897	***	***	***									
Inception v3	***	***	0.6329	0.8204	***	***								
**AlexNet**	***	***	0.2463	0.4532	***	***	0.9602							
SqueezeNet	***	***	***	***	***	***	***	***						
ResNet101	**	***	***	***	**~***(0.001)	***	***	***	***					
ResNet50	***	***	***	***	***	***	***	***	0.7306	***				
ResNet18	***	***	0.1564	0.1670	***	***	0.4655	0.5762	***	***	***			
GoogleNet	0.1187	***	***	***	**	***	***	***	***	0.1301	***	***		
ShuffleNet	***	0.6772	***	***	***	0.5722	***	***	***	***	***	***	***	

C

Independent two-sample t-test of training time, n = 40 (*p < 0.05, **p < 0.01, ***p < 0.001), Red unit, the row is superior than the column; Green unit, the column is superior than the row														
CNNs	VGG19	VGG16	DenseNet201	DarkNet53	NasNetMobile	DarkNet19	Inception v3	**AlexNet**	SqueezeNet	ResNet101	ResNet50	ResNet18	GoogleNet	ShuffleNet
VGG19														
VGG16	*													
DenseNet201	***	***												
DarkNet53	***	***	***											
NasNetMobile	***	***	***	***										
DarkNet19	***	***	***	***	***									
Inception v3	***	***	***	***	***	**								
**AlexNet**	***	***	***	***	***	***	***							
SqueezeNet	***	***	***	***	***	***	***	***						
ResNet101	***	***	***	***	***	***	***	***	***					
ResNet50	***	***	***	***	***	***	***	***	***	**				
ResNet18	***	***	***	***	***	***	***	***	***	***	***			
GoogleNet	***	***	***	***	***	***	***	***	***	***	***	0.0628		
ShuffleNet	***	***	***	***	***	***	***	***	***	***	***	0.2717	0.1590	

Supplementary Figure G2 | The optimization of the CNN model. A. The performances of CNNs. **B.** Independent two-sample t-test of AUC, n = 40 (*p < 0.05, **p < 0.01, ***p < 0.001). **C.** Independent two-sample t-test of training time, n = 40 (*p < 0.05, **p < 0.01, ***p < 0.001).

Appendix H | Feature selection

The feature selection process in this paper was divided into two steps. First, in order to obtain stable and repeatable features to eliminate the influence of unstable factors on the results during preprocessing and manual annotation, we invited four participants to delineate the ROI volume in the data collection twice (described before). For each CT scan, a total of 3404 features were extracted via intra-tumoral (1702 intra-radiomics features) and peri-tumoral (1702 peri-radiomics features) 3-mm VOI delineations. Considering the reproducibility of the study, we chose ICC values as a criterion to measure whether these features could be consistently extracted by the same observer (namely the participant) twice (according to the intra-group ICC values), and evaluated the consistency of these features across observers by inter-group ICC values. The figure below shows the ICC-based feature filter results, which showed good intra-group reproducibility and inter-group consistency for both the baseline CT radiomics and the follow-up CT radiomics features, as well as the time-based slope calculation results (i.e., delta-radiomics features). The ICC screening of the follow-up information extracted from the follow-up CT and the Delta-radiomics information (criterion: ICC > 0.75) determined in a total of 2527 features of reliable repeatability.

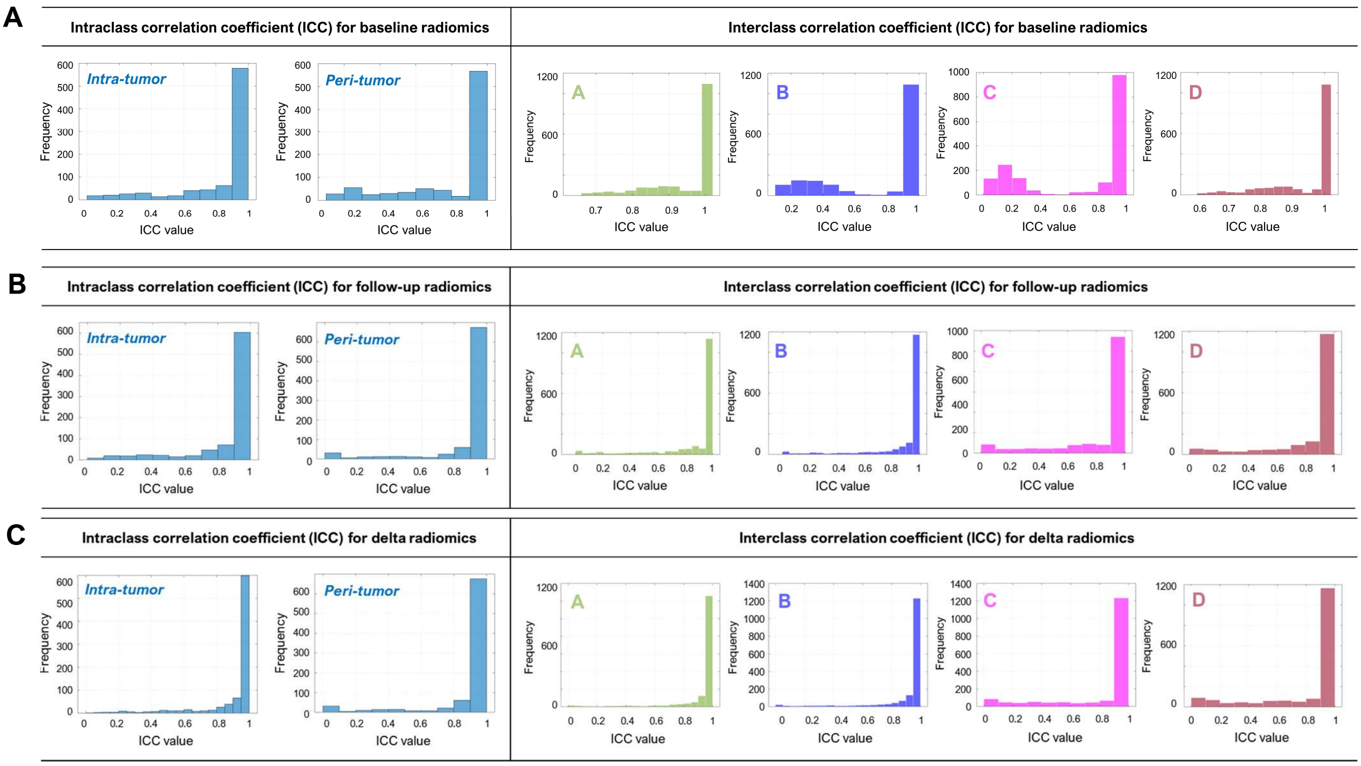

Supplementary Figure H | The intraclass and interclass correlation coefficient values for baseline radiomics (A), follow-up radiomics (B), and delta-radiomics (C).

Features should also be informative, i.e., being highly correlated with the target variables, non-redundant, and having good descriptiveness of the target properties that the task requires. For this purpose, feature selection algorithms were used to select the most informative features, and a total of 45 feature selection algorithms were taken into account. It must be noted that for each algorithm, which includes a certain number of parameters, the Bayes optimizer is selected to optimize the parameters in the actual training process with the

expected improvement per second (plus) as the objective function in order to obtain the optimal parameters for cross-algorithm comparison and find the optimized selection algorithm with the best performance. The feature selection methods used in this paper and their characteristics are given in the following table.

Supplementary Table H | Feature selection methods

Feature selection methods (abbreviations)	Feature selection methods (full name)	Basic principle / characteristics	Default parameters in this study
ABC	Artificial Bee Colony	One of the bionic algorithms for simulating virtual bee colonies, including three types of individuals: hired bees, observation bees and scouting bees	max = 5
ABO	Artificial Butterfly Optimization	Based on the butterfly optimization algorithm dividing the virtual butterflies into two groups, the optimal algorithm is explored according to the different behaviors of sunspot and canopy butterflies.	stepe = 0.05 (control number of sunspot), ratio = 0.2 (control step), ty = 1 (version 1 or 2)
ACO	Ant Colony Optimization	Simulating the accumulation of pheromones in ant colony foraging positive feedback mechanism to select the optimal path to reach the goal	/
ACS	Ant Colony System	The pseudo-random scaling rule is used for state transfer based on ACO, and only the global update rule is applied on the optimal ant path, and the local pheromone update rule is applied to the problem solution.	/
ALO	Ant Lion Optimizer	Mimics the behavior of an ant lion hunting ants and simulates the interaction between the ant lion and the ants in the trap	/
ASO	Atom Search Optimization	Simulation of global and local search by gravitational and repulsive forces in a molecular system composed of atoms that produce displacement according to Newton's laws of motion	alpha = 50 (depth weight), beta = 0.2 (multiplier weight)
BA	Bat Algorithm	The solution vector of the optimization problem is modeled as the position coordinates of the bat, and the optimal solution is found by the variation of the position of the bat echo localization	fmax = 2 (maximum frequency), fmin = 0 (minimum frequency), alpha = 0.9 (constant), gamma = 0.9 (constant), A = 2 (maximum loudness), r = 1 (maximum pulse rate)
BOA	Butterfly Optimization Algorithm	Inspired by simulated butterfly foraging and mating behavior, butterflies pick up or perceive and analyze airborne odors to determine the potential direction of a target	c = 0.01 (modular modality), p = 0.8 (switch probability)
CS	Cuckoo Search Algorithm	A balanced combination of local random wandering and global exploratory random wandering using the cuckoo's parasitic brood model, allowing for more effective exploration on a global scale	Pa = 0.25 (discovery rate)

CSA	Crow Search Algorithm	Simulate the pattern of a crow storing/retrieving food to avoid theft to handle some simple function problems	AP = 0.1 (awareness probability), fl = 1.5 (flight length)
DE	Differential Evolution	Each individual in the population competes with each other to generate intelligence to continuously retain the good individuals and eliminate the bad ones in order to approximate the optimal solution	CR = 0.9 (crossover rate), F = 0.5 (constant factor)
EO	Equilibrium Optimizer	Simulates the phenomenon of controlled volume mass balance by controlling the entry, exit and production of mass in the volume to find the equilibrium candidate, i.e. the optimal solution	a1 = 2 (constant), a2 = 1 (constant), GP = 0.5 (generation probability)
EPO	Emperor Penguin Optimizer	Simulating the solidarity and division of labor of emperor penguins in winter clusters for heating leads each individual to continuously change its position and move towards the optimal point to find the optimal solution	M = 2 (movement parameter), f = 3 (control parameter), l = 2 (control parameter)
FA	Firefly Algorithm	Simulate the process of firefly luminous attraction courtship, according to the principle of light brightness decay with distance to guide the individual to the best advantage	alpha = 1 (constant), beta0 = 1 (light amplitude), gamma = 1 (absorbtion coefficient), theta = 0.97 (control alpha)
FOA	Fruitfly Optimization Algorithm	Simulate the foraging process of fruit flies, and simulate the global exploration process as the olfactory stage and the local development process as the visual stage to continuously approach the optimal result	/
FPA	Flower Pollination Algorithm	The process of pollen dispersal is simulated, the local optimization is simulated as self-pollination process, and the global optimization stage is simulated as heterogeneous pollination process and the balance of these two processes is controlled by switching probabilities to coordinate the local performance and global performance of the algorithm	/
GA	Genetic Algorithm	Variation/crossover/recombination/genetic inheritance of characteristics of a large number of individuals to optimize the best solution, simulating the process of natural selection and reproduction	/
GNDO	Generalized Normal Distribution Optimization	Simulation of the established generalized normal distribution model for local open method and global development based on three randomly selected individuals	/
GSA	Gravitational Search Algorithm	Based on the law of gravity and Newton's law of motion to simulate the interaction between particles, optimize the position of particles to find the optimal solution	/
GWO	Grey Wolf Optimizer	Simulate the hierarchy and hunting behavior of gray wolves, and conduct global search through three stages of encirclement-hunting-attack to find the optimal solution	/

HGSO	Henry Gas Solubility Optimization	According to Henry's law, the dissolution and release of gas at a fixed temperature are simulated to optimize the balance between the development process and the search process	Nc = 2 (number of gas types/cluster), K = 1 (constant), alpha = 1 (influence of other gas), beta = 1 (constant), L1 = 5E-3 (constant), L2 = 100 (constant), L3 = 1E-2 (constant)
HHO	Harris Hawks Optimization	Simulate Harris Hawk's strain on the way of prey's escape in different situations to adjust the balance of exploitation and search, and finally find the best coordinates of the target	/
HLO	Human Learning Optimization	Simulate the pattern of human learning, based on three learning operators random learning operator, individual learning operator and social learning operator to search and develop	pi = 0.85 (probability of individual learning), pr = 0.1 (probability of exploration learning)
HS	Harmony Search	Simulate the pattern of an orchestra ensemble and adjust the harmonic weights of different individuals until the optimal solution is reached	/
JA	Jaya Algorithm	By traversing all the individuals in the population, finding the current best and worst individuals and updating the individual parameters, the current individuals keep avoiding harm and keep approaching the optimal solution	/
LASSO	Least Absolute Shrinkage and Selection Operator	Construct a more refined model by penalizing the function to compress the regression coefficients and retain a subset of shrinkage by setting some regression coefficients to zero	alpha = 0.1/0.2/0.5/0.8
MBO	Monarch Butterfly Optimization	Simulate the migratory behavior of monarch butterflies to make a feasible solution for the butterfly's location simulation and find the optimal location through both migration and adaptation processes	peri = 1.2 (migration period), p = 5/12 (ratio), Smax = 1 (maximum step), BAR = 5/12 (butterfly adjusting rate), N1 = 4 (number of butterflies in land 1)
MFO	Moth Flame Optimization	Mimicking the moth's directional navigation mechanism allows the individual to continuously approach the target and search the space more extensively	b = 1 (constant)
MPA	Marine Predators Algorithm	Simulate the hunting behavior of predators in the ocean, and achieve the purpose of finding the best through many different random generation strategies	P = 0.5 (constant), FADs = 0.2 (fish aggregating devices effect)
MRFO	Manta Ray Foraging Optimization	Simulate the foraging behavior of manta rays by three different foraging patterns: chain, spiral and tumbling behavior to determine the target location	S = 2 (somersault factor)
MVO	Multiverse Optimizer	Based on the multiverse theory, the formation of black holes, white holes and wormholes is simulated to iterate the universe in a loop to drive objects to the optimal universe	p = 6 (control TDR), Wmax = 1 (maximum WEP), Wmin = 0.2 (minimum WEP)

PFA	Path Finder Algorithm	Simulating the foraging activities of a population led by a leader, selecting the leader by fitness and continuously iterating to find the best	/
PRO	Poor And Rich Optimization	Simulate different patterns of wealth growth for the poor and the rich to reduce/increase the class gap phenomenon to iterate for the purpose of meritocracy	Pmut = 0.06 (mutation probability)
PSO	Particle Swarm Optimization	Simulate the behavioral pattern of foraging birds to perform global search and find the global optimal solution	c1 = 2 (cognitive factor), c2 = 2 (social factor), w = 0.9 (inertia weight)
SA	Simulated Annealing	Simulates the process of heating-annealing a non-crystal to a crystal and accepts a worse solution with a certain probability to jump out of the local optimum and reach the global optimum	c = 0.93 (cooling rate), T0 = 100 (initial temperature)
SBO	Satin Bower Bird Optimization	Simulating the courtship process of the satin blue gardener bird by calculating individual fitness, updating the population, and mutating individuals to continuously iterate to find the best	/
SCA	Sine Cosine Algorithm	The optimal solution is found by constructing multiple alternative random solutions and then substituting into the sine and cosine model to optimize the solution	alpha = 2 (constant)
SMA	Slime Mould Algorithm	Adaptive weights are constructed to simulate the predatory behavior of slime bacteria to generate feedback information from the biological oscillator of slime bacteria, and the best path to the target is constructed accordingly	/
SOS	Symbiotic Organisms Search	Simulate the mutualistic-symbiotic-parasitic three stages of symbiotic organisms to calculate the degree of adaptation to iteratively eliminate and finally find the optimal solution	/
SSA	Salp Swarm Algorithm	Simulate the predatory behavior of a chain of bottlenose sea squirts, distinguish leaders and followers by fitness, and iterate continuously to find the optimal solution that ranks first in the chain	/
TGA	Tree Growth Algorithm	Simulate the process of trees competing for sunlight and nutrients, and build the optimal growth pattern based on the unique vectorial movement of plants to adapt to the corresponding problem environment to find the global optimal solution	N1 = 3 (size of first group), N2 = 5 (size of second group), N4 = 3 (size of fourth group), theta = 0.8 (tree reduction rate of power), lambda = 0.5 (control nearest tree)
TSA	Tree Seed Algorithm	Simulate tree reproduction to adjust the global search and local search balance to achieve fast convergence to the optimal solution	ST = 0.1 (switch probability)
WOA	Whale Optimization Algorithm	Simulate the prey hunting behavior of whales and converge quickly to the optimal solution by encircling/bubble net repelling mode	b = 1 (constant)

WSA	Weighted Superposition Attraction	Simulate the principle of superposition of particles in physics, so that the superposition of individuals in the population, so as to lead other alternative solutions to change to avoid falling into the local optimum to achieve the global optimum	tau = 0.8 (constant), sl = 0.035 (step length), phi = 0.001 (constant), lambda = 0.75 (constant)

Appendix I | Classifiers considered in this study

The essence of classification is to provide known input data and perform supervised machine learning on the known labels or classes of the data. In this paper, we consider the following 32 classification algorithms, which can be found in the table below.

Supplementary Table I | The classifiers used in this work

Major categories	Classifiers	Core characteristics	
		Interpretability	Flexibility
Decision Trees, DT	Coarse Tree	A	C (Maximum number of divisions: 4)
	Medium Tree	A	B (Maximum number of divisions: 20)
	Fine Tree	A	A (Maximum number of divisions: 100)
Discriminant Analysis, DA	Linear Discriminant	A	C
	Quadratic Discriminant	A	C
Logistic Regression, LR		A	C
Naive Bayes Classifiers, NBC	Gaussian Naive Bayes	A	C
	Kernel Naive Bayes	A	B
Support Vector Machines, SVM	Linear SVM	A	C
	Quadratic SVM	C	B
	Cubic SVM	C	B
	Fine Gaussian SVM	C	Generally A
	Medium Gaussian SVM	C	B
	Coarse Gaussian SVM	C	A
K Nearest Neighbor Classifiers	Fine KNN	C	Depends
	Medium KNN	C	Depends
	Coarse KNN	C	Depends
	Cosine KNN	C	Depends
	Cubic KNN	C	Depends
	Weighted KNN	C	Depends
Kernel Approximation Classifiers	SVM Kernel	C	B
	Logistic Regression Kernel	C	B
Ensemble Classifiers	Boosted Trees	C	B
	Bagged Trees	C	A-B
	Subspace Discriminant	C	A
	RUSBoost Trees	C	B

	Subspace KNN	C	B
Neural Network Classifiers	Narrow Neural Network	C	B
	Medium Neural Network	C	B
	Wide Neural Network	C	B
	Bilayered Neural Network	C	A
	Trilayered Neural Network	C	A

Appendix J | Model evaluation methods

J1 Performance metrices

The samples applied in this paper are unbalanced (STAS (+) : STAS (-) = 143 : 442 in the main cohort). The AUC calculation takes into account both the classification ability of the machine learner for positive and negative examples, and still allows a reasonable evaluation of the classifier despite the imbalanced samples. In this way, we mainly applied the ROC (Receiver Operating Characteristic)-AUC for the evaluation of the model performances.

J2 Attention model for deep-learning networks

Although CNNs (Convolutional Neural Networks) are very effective at classifying many tasks, the content and rules they learn are difficult for humans to understand, commonly known as 'black boxes'. It would be useful to visualize CNNs in order to understand the models.
Grad-CAM (Class Activation Map) is one such tool. In this paper, we applied it to visualize the attention of the model. CAM is also known as a category heat map, saliency map, etc. It is a graph of equal size to the original image, and the pixels at each location on that image take values ranging from 0 to 1. In this paper it is represented by applying a color scale in MATLAB. In general, it can be interpreted as the distribution of contributions to the predicted output, with higher scores indicating a higher response and contribution to the network from the corresponding region of the original image. Grad-CAM uses a global average of the gradients to calculate the weights. It first defines the importance weight α_k^c of feature map *k* (i.e., representing the k-th channel in feature layer A) for classification *c* as:

$$\alpha_k^c = \frac{1}{Z}\sum_i \sum_j \frac{\partial y^c}{\partial A_{ij}^k}$$

Applying this weight, the last layer of the feature map is weighted and, after linear combination, input into the ReLU activation function to obtain:

$$L_{\text{Grad-CAM}}^c = ReLU\left(\sum_k \alpha_k^c A^k\right)$$

In essence, for a specified image to be classified, it is preprocessed to obtain the gradient of the model output relative to the activation output of the last convolutional layer, and then homogenized to process the gradient, establish a functional relationship between the model output, the activation output of the last convolutional layer and the gradient mean, and use the gradient value to multiply the feature value of each channel of the last convolutional layer, that is, to obtain the importance of each point to the final classification result of the model. The class activation map is rendered as a heatmap after adjustment, and after overlaying with the original image, the visualization result of the contribution of each point position (i, j) to the classification c in the original image is realized.

Appendix K | Model repetitiveness (*N* = 40)

Supplementary Table K1 | The AUC values and other model metrics with their SD values (Dual-delta model)

Classification tasks	AUC values	Accuracy/%	Sensitivity/%	Specificity/%
Without PSM (5-fold)	0.92 ± 0.02	87.54 ± 2.40	73.60 ± 3.52	92.77 ± 2.54
With PSM (10-fold)	0.90 ± 0.02	86.23 ± 2.55	73.45 ± 2.88	92.55 ± 2.31
Without PSM (In-center validation)	0.94 ± 0.02	87.20 ± 1.95	72.52 ± 2.97	91.98 ± 1.98
With PSM (In-center validation)	0.91 ± 0.01	86.77 ± 2.01	80.33 ± 2.56	93.66 ± 2.17
External validations A (ZS Cohort)	0.84 ± 0.02	80.65 ± 2.25	75.32 ± 2.93	82.80 ± 1.70
External validations B (Ninth Cohort)	0.84 ± 0.02	86.77 ± 2.01	94.24 ± 4.25	74.63 ± 3.77

Supplementary Table K2 | The AUC values and other model metrics with their SD values (Dual-radiomics model)

Classification tasks	AUC values	Accuracy/%	Sensitivity/%	Specificity/%
Without PSM (5-fold)	0.85 ± 0.01	82.79 ± 0.98	72.45 ± 1.01	83.26 ± 1.33
With PSM (10-fold)	0.83 ± 0.01	80.25 ± 1.73	72.00 ± 1.75	86.98 ± 1.20
Without PSM (In-center validation)	0.87 ± 0.01	84.22 ± 1.29	72.25 ± 0.97	87.15 ± 0.79
With PSM (In-center validation)	0.82 ± 0.01	80.10 ± 1.09	76.54 ± 1.33	82.12 ± 2.02

Supplementary Table K3 | The AUC values and other model metrics with their SD values (Dual-DL model)

Classification tasks	AUC values	Accuracy/%	Sensitivity/%	Specificity/%
Without PSM (5-fold)	0.82 ± 0.03	79.15 ± 3.10	70.22 ± 2.35	79.56 ± 3.46
With PSM (10-fold)	0.81 ± 0.03	80.73 ± 4.01	71.98 ± 3.04	84.59 ± 2.57

Without PSM (In-center validation)	0.81 ± 0.03	78.99 ± 2.78	71.09 ± 2.54	80.33 ± 3.51
With PSM (In-center validation)	0.83 ± 0.04	83.25 ± 3.77	70.95 ± 2.54	83.82 ± 3.49

Supplementary Table K4 | The AUC values and other model metrics with their SD values (Pure-DL model)

Classification tasks	AUC values	Accuracy/%	Sensitivity/%	Specificity/%
Without PSM (5-fold)	0.78 ± 0.04	76.36 ± 4.53	71.62 ± 4.56	78.22 ± 4.72
With PSM (10-fold)	0.81 ± 0.05	79.92 ± 3.67	74.25 ± 5.13	85.22 ± 4.35
Without PSM (In-center validation)	0.76 ± 0.03	75.87 ± 4.54	69.48 ± 4.25	82.43 ± 2.96
With PSM (In-center validation)	0.77 ± 0.05	79.98 ± 5.61	72.53 ± 5.25	81.67 ± 5.20

Supplementary Table K5 | The AUC values and other model metrics with their SD values (Pure radiomics model)

Classification tasks	AUC values	Accuracy/%	Sensitivity/%	Specificity/%
Without PSM (5-fold)	0.77 ± 0.02	76.99 ± 1.37	71.98 ± 0.98	80.25 ± 0.92
With PSM (10-fold)	0.82 ± 0.02	80.46 ± 0.94	73.91 ± 0.73	88.42 ± 1.88
Without PSM (In-center validation)	0.84 ± 0.02	80.85 ± 1.95	70.59 ± 1.33	87.06 ± 1.71
With PSM (In-center validation)	0.82 ± 0.02	82.68 ± 1.50	70.43 ± 0.99	82.97 ± 0.91

Supplementary Table K6 | The AUC values and other model metrics with SD values (Pure delta-radiomics model)

Classification tasks	AUC values	Accuracy/%	Sensitivity/%	Specificity/%
Without PSM (5-fold)	0.80 ± 0.01	79.81 ± 1.15	70.34 ± 0.69	84.81 ± 0.91
With PSM (10-fold)	0.81 ± 0.02	80.21 ± 0.88	74.12 ± 0.90	87.31 ± 0.95

Without PSM (In-center validation)	0.82 ± 0.02	79.95 ± 0.97	73.13 ± 0.88	89.29 ± 1.66
With PSM (In-center validation)	0.80 ± 0.01	81.44 ± 1.09	69.51 ± 1.87	85.24 ± 1.20

Supplementary Table K7 | The AUC values and other model metrics with their SD values (Pure delta-DL model)

Classification tasks	AUC values	Accuracy/%	Sensitivity/%	Specificity/%
Without PSM (5-fold)	0.81 ± 0.03	79.52 ± 4.97	70.24 ± 3.98	81.69 ± 4.12
With PSM (10-fold)	0.79 ± 0.04	79.81 ± 4.19	75.81 ± 5.04	83.68 ± 4.47
Without PSM (In-center validation)	0.78 ± 0.04	77.01 ± 5.13	70.28 ± 5.00	81.25 ± 4.08
With PSM (In-center validation)	0.81 ± 0.04	80.72 ± 4.87	69.23 ± 5.52	92.21 ± 4.80

Supplementary Table K8 | The AUC values and other model metrics with their SD values (Merged delta features)

Classification tasks	AUC values	Accuracy/%	Sensitivity/%	Specificity/%
Without PSM (5-fold)	0.86 ± 0.02	83.98 ± 0.97	69.54 ± 1.28	87.33 ± 2.03
With PSM (10-fold)	0.87 ± 0.02	85.81 ± 1.67	74.35 ± 1.88	89.03 ± 1.29
Without PSM (In-center validation)	0.89 ± 0.02	87.31 ± 2.04	70.88 ± 1.06	85.65 ± 0.88
With PSM (In-center validation)	0.86 ± 0.03	86.20 ± 1.35	69.82 ± 2.09	89.42 ± 0.96

Supplementary Table K9 | The AUC values and other model metrics with their SD values (Merged classic features)

Classification tasks	AUC values	Accuracy/%	Sensitivity/%	Specificity/%
Without PSM (5-fold)	0.83 ± 0.01	80.25 ± 0.75	70.02 ± 1.01	85.91 ± 1.92
With PSM (10-fold)	0.81 ± 0.02	80.20 ± 1.27	67.55 ± 1.42	87.53 ± 0.88

Without PSM (In-center validation)	0.84 ± 0.02	84.52 ± 1.72	71.49 ± 1.40	82.75 ± 0.92
With PSM (In-center validation)	0.82 ± 0.01	81.54 ± 2.00	69.38 ± 1.27	87.53 ± 0.99

Supplementary Table K10 | The AUC values and other metrics with SD values (Delta-DL with non-delta radiomics)

Classification tasks	AUC values	Accuracy/%	Sensitivity/%	Specificity/%
Without PSM (5-fold)	0.88 ± 0.03	86.32 ± 1.55	71.21 ± 2.28	87.29 ± 1.86
With PSM (10-fold)	0.86 ± 0.03	85.12 ± 2.53	69.34 ± 2.13	88.82 ± 2.11
Without PSM (In-center validation)	0.88 ± 0.02	85.81 ± 2.24	70.18 ± 2.72	86.23 ± 2.12
With PSM (In-center validation)	0.90± 0.03	86.29 ± 2.40	70.20 ± 2.11	92.82 ± 1.98

Supplementary Table K11 | The AUC values and other metrics with SD values (Delta-radiomics with non-delta DL)

Classification tasks	AUC values	Accuracy/%	Sensitivity/%	Specificity/%
Without PSM (5-fold)	0.84 ± 0.03	83.14 ± 3.52	70.23 ± 2.71	85.42 ± 4.01
With PSM (10-fold)	0.83 ± 0.04	83.52 ± 5.09	69.59 ± 3.88	87.24 ± 3.97
Without PSM (In-center validation)	0.88 ± 0.04	85.98 ± 3.01	70.53 ± 2.96	90.19 ± 3.05
With PSM (In-center validation)	0.89 ± 0.02	86.88 ± 1.82	72.23 ± 3.23	93.72 ± 1.67

Supplementary Table K12 | Pairwise comparison results ($P < 0.05$*, $P < 0.01$**, $P < 0.001$***)

Metrics	Groups	Without PSM (5-fold)	With PSM (10-fold)	Without PSM (In-center validation)	With PSM (In-center validation)
**AUC**	A vs B	***	***	***	***
	A vs C	***	***	***	***
	A vs D	***	***	***	***
	A vs E	***	***	***	***
	A vs F	***	***	***	***
	A vs G	***	***	***	***
	A vs H	***	***	***	***
	A vs I	***	***	***	***
	A vs J	***	***	***	***
	A vs K	***	***	***	***
**Accuracy**	A vs B	***	***	***	***
	A vs C	***	***	***	***
	A vs D	***	***	***	***
	A vs E	***	***	***	***
	A vs F	***	***	***	***
	A vs G	***	***	***	***
	A vs H	***	0.2596	0.1430	0.4561
	A vs I	***	***	***	***
	A vs J	0.3119	0.0062**	0.0081**	0.0059**
	A vs K	***	0.0034**	0.0937	0.9936
**Sensitivity**	A vs B	0.0279*	0.0021**	0.3950	***
	A vs C	***	0.0044**	0.0208*	***
	A vs D	0.1644	0.3784	***	***
	A vs E	0.0031**	0.9035	***	***
	A vs F	***	0.2390	0.1439	***
	A vs G	***	0.0011**	0.3176	***
	A vs H	***	0.0194*	***	***
	A vs I	***	***	***	***
	A vs J	***	***	***	***
	A vs K	***	***	***	***
**Specificity**	A vs B	***	***	***	***
	A vs C	***	***	***	***
	A vs D	***	***	***	***

	A vs E	***	***	***	***
	A vs F	***	***	***	***
	A vs G	***	***	***	0.1249
	A vs H	***	***	***	***
	A vs I	***	***	***	***
	A vs J	***	***	***	0.0098**
	A vs K	***	***	0.0023**	0.4514

Appendix L | The results of PSM and their analysis

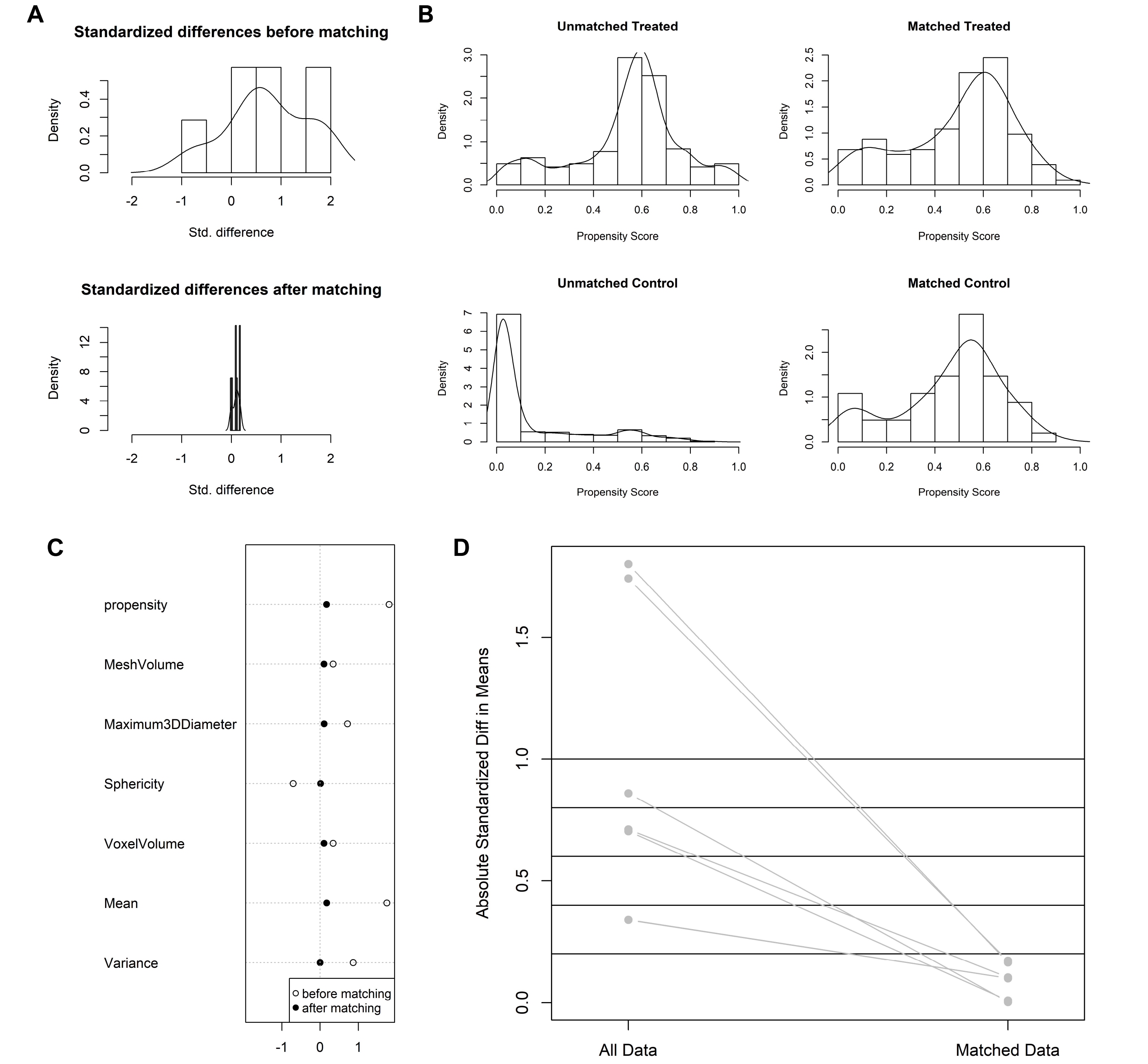

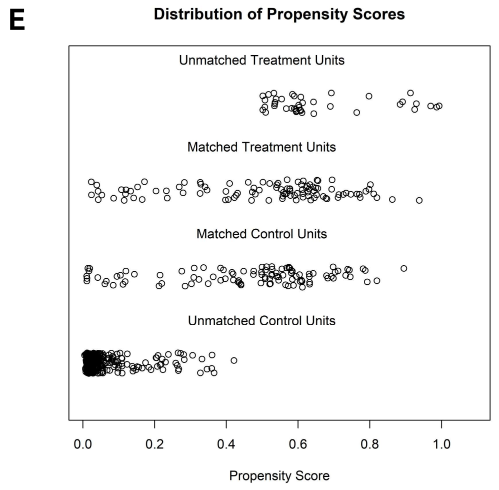

F

Sample Sizes								
Subsamples	All		Matched		Unmatched		Discarded	
	Control	Treated	Control	Treated	Control	Treated	Control	Treated
(all cases)	442	143	102	102	340	41	0	0

Overall balance test (Hansen & Bowers, 2010)			
Statistics	chisquare	df	p.value
(all cases)	3.469	6.000	0.748

Relative multivariate imbalance L1 (Iacus, King, & Porro, 2010)		
Statistics	Before matching	After matching
(all cases)	0.927	0.824

Summary of unbalanced covariates (|d| > .25)
No covariate exhibits a large imbalance (|d| > .25).

Supplementary Figure L1 | The results of propensity score matching (PSM) for balancing the essential first-order features in STAS(+) and STAS(-) groups. **A.** Histogram of SD distribution. **B.** Histogram of distribution of propensity

scores before and after the PSM (upper: STAS positive, lower: STAS negative). **C.** Univariate SD scatterplot. **D.** Line of variation plot. **E.** Case jitter scatterplot. **F.** Selected significant test results.

We applied IBM SPSS Statistics 22 software (with additional PS matching 3.0.4 toolkit) to match some of the important 1D radiomics (some essential shape features and first-order statistical features) information of the patients, forming a matched cohort of STAS (+): STAS (-) = 120: 120. This matching was implemented based on the Propensity Score Matching (PSM) algorithm first proposed by Rosenbaum and Rubin. The parameters selected for matching are as follows: Mesh Volume, Maximum 3D Diameter, Sphericity, Voxel Volume, Mean and Variance, a total of six covariates used to calculate the propensity score; the matching algorithm, Nearest Neighbor Matching (NNM); Match Tolerance, 0.05; Match Ratio, 1:1; estimation algorithm, Logistic regression; no playback (replacement) was allowed because reuse of controls weakened the sample independence of the control group.

The significances of matching the above six one-dimensional radiomics information of 143 STAS (+) patients in the STAS (-) patient population were as follows. First, we aimed to test the differential diagnostic value of the high-dimensional histological information and the newly added post-registration and subtracted DL model for nodules/masses with similar one-dimensional morphology. Second, we performed the PSM to improve the balance of the learning samples of the machine learning model to further examine the model performances with a balanced dataset. Third, this design could simulate the actual medical scenarios of differential diagnosis needs to further validate the real-world value of the model.

As a result, among 442 patients with STAS (-), a total of 120 patients were successfully matched with 120 patients with STAS (+) by their 1D baseline information. As seen from the matching results, the L1 measure statistic in the relative multivariate imbalance L1 test decreased from 0.927 to 0.824 before and after PSM, suggesting an improvement in variable imbalance after matching, and the amount of standardized mean difference ($|d| > 0.25$) suggested that no imbalance covariates were seen after matching. Histogram **A** of the SD distribution showed that the standard deviation was around 0 after matching, which testified a significant improvement from the pre-matching situation. Univariate SD scatter plot **C** and line plot of variation **D** suggested a significant improvement in variable balance, which was further demonstrated by Hansen & Bowers global balance test with $p = 0.748 > 0.05$. The histogram **B** showing the distribution of propensity values demonstrated the similarity between the matched propensity values of two groups, where the density function curves of the treated and control groups after PSM were approximate, suggesting good matching effect. The high overlap of the two groups on the case jitter scatter plot **E** also supported this conclusion. We performed the same statistical analysis of various information in the new patient cohort after matching, and the results suggested that although the covariates in PSM did not contain clinical variables, there was an improvement in the balance of most clinical and radiological indicators between the two groups after PSM, suggesting that patient populations with similar one-dimensional radiomics information tended to have more similar clinical and imaging performance, which was consistent with our observations in the clinical scenario.

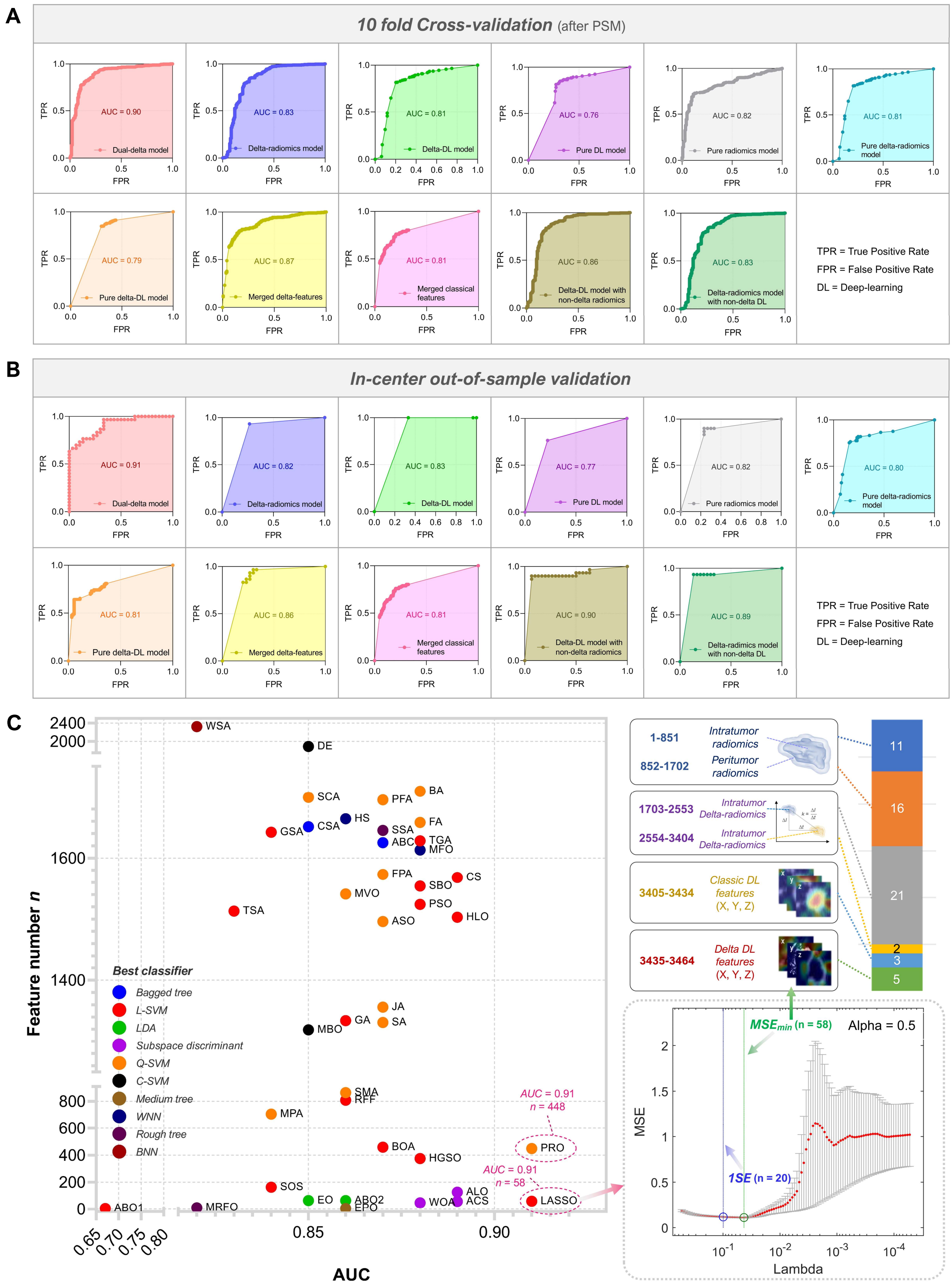

Supplementary Figure L2 | Results of five-fold cross-validation and in-center validation after PSM. A. Ten-fold cross-validation ROC curves and their AUC values. **B.** In-center validation ROC curves and their AUC values. **C.** AUC values and the feature numbers for the combinations of feature selection algorithms and their optimal classification models, where the LASSO cross-validation plot (green vertical lines represent the number of features corresponding to MSEmin), and the pie chart showing the composition of essential feature set selected by LASSO are given.

Appendix M | Visualization of model attention with Grad-CAM

A **Attention distribution of the deep-learning model (Classical)**

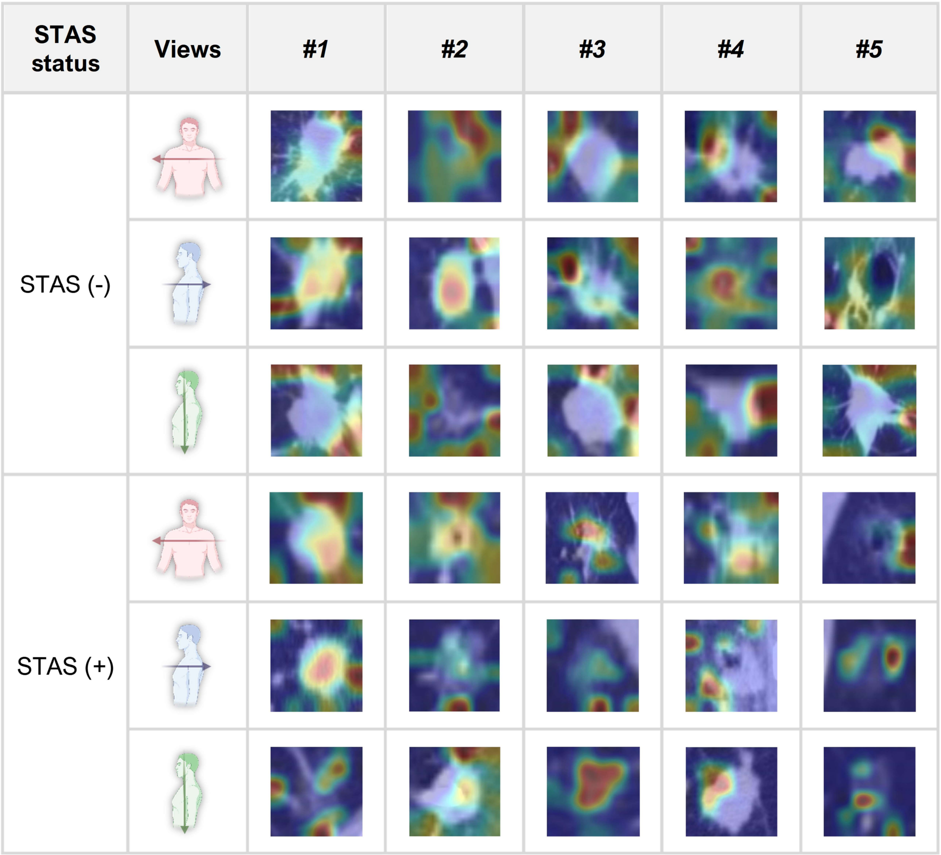

B **Attention distribution of the deep-learning model (Delta)**

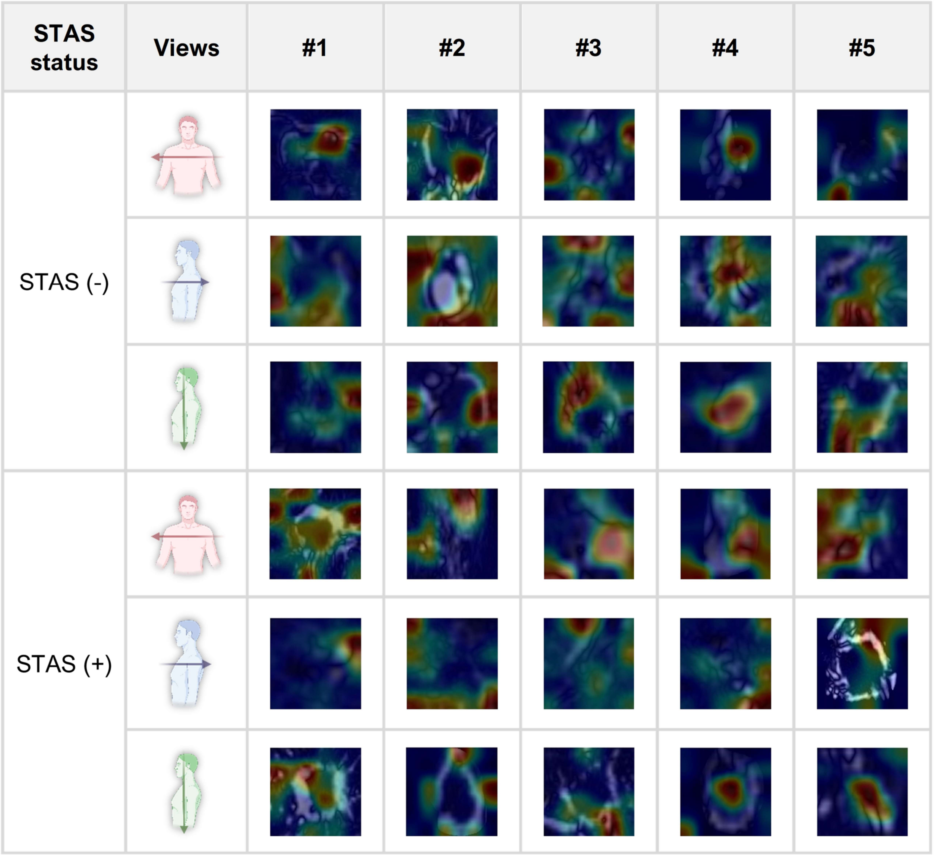

Supplementary Figure M | Grad-CAM visualization results. **A.** Attention map of classic deep learning model. **B.** Attention of Delta deep learning model.

Appendix N | On the interpretability of the features

To systematically understand the effectiveness of each radiomics feature and DL feature in the classification task, we selected VOI results and extracted radiomics information by **Participant A**, who has relatively the most experience in medical image interpreting and processing. The radiomics from the VOI and 3D field of view images corresponding to all 585 patients in the CHEST Cohort were used for training (to fully understand the values of features, we processed the radiomics data without ICC-based feature filtering). In order to discover the core set of features that were most valuable for the classification performance, we considered the results of the LASSO regression algorithm with alpha = 0.5 and the set of the corresponding top 38 significant features filtered by the ReliefF feature ranking algorithm (the parameter K in the algorithm was empirically taken as 10), and the results were presented in the following table. The coding of the features was given in the article, where features 1-851 were classic radiomics features within the tumor, features 852-1702 corresponded to classic radiomics features at 3 mm around the tumor, features 1703-2553 corresponded to intratumoral delta-radiomics features, features 2554-3404 corresponded to delta-radiomics features of the peri-tumor volumes, features 3405-3434 were classic DL features, and delta-DL features were recorded as features 3435-3464.

The results of LASSO regression suggested that a total of 38 features (i.e., the 38 degrees of freedom shown in the middle-upper panel of **Figure 8B**, corresponding to the green line) corresponded to the minimum MSE (Mean squared error). ReliefF gave information on the weights of the feature ranking, and it could be seen that the classic radiomics features were relatively robust in their classification importance, and there were few features with negative weights (i.e., those that interfered with the classification results). However, there were also fewer features that contributed significantly to the classification, which suggested that the classic radiomics features were feature set with conservative performances. Unlike the classic features, delta-radiomics features and both DL features had larger absolute values in weights, i.e., they included features that contributed significantly to the classification results, which also showed that further feature screening not only reduced the dimensionality of the model and improved the efficiency of training and computing, but also had great significance for ensuring the classification accuracy of the model.

Figure 8C and the table below further give the set of important features obtained by the feature filtering algorithm. **Figure 8C** shows the features with the top 38 weight rankings and their corresponding weights provided by the ReliefF algorithm. The sets of important features provided by the two algorithms did not exactly overlap, suggesting that there might be significant differences in the feature spaces composed when different feature selection algorithms were chosen. Further analysis suggests that LASSO regression was more suitable for this task (LASSO AUC = 0.901, ReliefF AUC = 0.843) than ReliefF selection combined with trial-and-error method, suggesting that the selection of a sensitive feature selection method to construct a feature space suitable for the task scenario was extremely significant for the classification quality. Meanwhile, it is easy to see that both the LASSO regression and ReliefF algorithms provided important feature sets that contained a large number of delta-radiomics features (LASSO: 28, ReliefF: 12), while the inclusion rate of classic radiomics features, although higher among the features filtered by ReliefF, was limited in the LASSO

regression results where they were significantly less involved than the other feature groups, with only 0.47% (8/1702) of the inclusion rate (Delta-radiomics: 28/1702 = 1.6%, classic deep learning model: 1/30 = 3.3%, delta deep learning model: 1/30 = 3.3%), which suggested that the other feature combinations had relatively higher information abundance (especially delta-radiomics information) than classic radiomics features. It was of great significance for the complement of classic radiomics to involve the other feature sets into the final classification model, which was, actually, demonstrated from the ablation study results shown in both **Figure 5C-E** and **Figure 6A-B.**

Whether the results were subjected to LASSO regression or to ReliefF feature ranking, the radiomics indicators in them showed good interpretability and enlightenment. For example, for the LASSO regression results, six classic radiomics features were selected, for example: the Maximum Probability which describes the texture feature with the most occurrences in the image, i.e., the probability of the maximum pixel pair, which indicated that the texture features defined by GLCM were meaningful for classification, especially the specific texture patterns in them that might be different among STAS positive and negative populations. The MCC defined the texture complexity, especially the component filtered by LLH, played important role in classification, which suggested that the texture complexity of the image after two approximations and one edging could help distinguish the STAS(-) and STAS(+). These features demonstrated the texture heterogeneity in a specific frequency domain might be a criterion for STAS diagnosis, although the frequency domain transformation process here was not necessarily instantaneous for the human eye and this texture heterogeneity cannot be evaluated instantaneously and quantitatively by the human observation. Twenty-eight delta-radiomics features were selected, among which the first-order features and shape features of the original images were well understood. For example, the feature Elongation reflected the relationship between the two largest principal components of the ROI shape, and this principal component analysis was performed using the physical coordinates of the voxel centers defining the ROI, i.e., reflecting the elongation of the image. δ-Elongation reflected the change in elongation of the image, i.e., the stretching growth of the tumor during the follow-up. Similarly, δ-Flatness measured the change of flatness in 3D volume during tumor follow-up, which was essentially a dynamic description of heterogeneous growth. δ-Median and δ-Skewness were quantitative descriptions of the histogram of CT values in VOIs, which essentially reflected the change of mean density and density heterogeneity of tumor and was also the information to be frequently considered during the current classic follow-up. δ-wavelet-LLL defined features occupy an important proportion of delta-radiomics features, which indicated that the sharp edge information may be relatively less important for the determination of STAS or not, while the texture information defined by GLCM and others had a greater classification value after low-pass filtering approximation.

Supplementary Figure N1 showed the importance of features in the prediction model calculated and ranked by ReliefF algorithm. The classic radiomics information was relatively robust in classification with only few features with negative weights. However, compared to the delta-radiomics features, the classic radiomics features had less contribution to the significant positive feature set. The delta-radiomics features and delta-DL features had larger absolute values of weights than those of classic radiomics, suggesting that they may

include information that contribute significantly to the classification results.

Supplementary Table N | Essential feature sets determined by LASSO and ReliefF

Selected by LASSO (alpha = 0.5)

Code	Image type	Peri/Intra-tumor	Feature class	Feature name
53	original	I	GLCM	Maximum Probability
145	wavelet-LLH	I	GLCM	MCC
251	wavelet-LHL	I	GLDM	Large Dependence High Gray Level Emphasis
289	wavelet-LHL	I	NGTDM	Busyness
432	wavelet-HLL	I	GLDM	Dependence Variance
767	wavelet-LLL	I	First-order	Mean
796	wavelet-LLL	I	GLCM	MCC
1247	wavelet-HLL	P	First-order	Median
1703	δ-original	I	δ-Shape	δ-Elongation
1704	δ-original	I	δ-shape	δ-Flatness
1726	δ-original	I	δ-First-order	δ-Median
1731	δ-original	I	δ-First-order	δ-Skewness
1762	δ-original	I	δ-GLDM	δ-Dependence Variance
1798	δ-original	I	δ-GLSZM	δ-Size Zone Non Uniformity Normalized
1836	δ-wavelet-LLH	I	δ-GLCM	δ-Difference Variance
1868	δ-wavelet-LLH	I	δ-GLRLM	δ-Gray Level Variance
1884	δ-wavelet-LLH	I	δ-GLSZM	δ-Gray Level Variance
2070	δ-wavelet-LHH	I	δ-GLSZM	δ-Gray Level Variance
2109	δ-wavelet-HLL	I	δ-GLCM	δ-Cluster Shade
2134	δ-wavelet-HLL	I	δ-GLDM	δ-Dependence Variance
2170	δ-wavelet-HLL	I	δ-GLSZM	δ-Size Zone Non Uniformity Normalized
2272	δ-wavelet-HLH	I	δ-NGTDM	δ-Complexity
2446	δ-wavelet-HHH	P	δ-GLSZM	δ-Large Area Low Gray Level Emphasis
2470	δ-wavelet-LLL	P	δ-First-order	δ-Median
2475	δ-wavelet-LLL	P	δ-First-order	δ-Skewness
2479	δ-wavelet-LLL	P	δ-GLCM	δ-Autocorrelation
2487	δ-wavelet-LLL	P	δ-GLCM	δ-Difference Variance
2495	δ-wavelet-LLL	P	δ-GLCM	δ-Joint Average
2500	δ-wavelet-LLL	P	δ-GLCM	δ-Sum Average
2506	δ-wavelet-LLL	P	δ-GLDM	δ-Dependence Variance
2509	δ-wavelet-LLL	P	δ-GLDM	δ-High Gray Level Emphasis
2519	δ-wavelet-LLL	P	δ-GLRLM	δ-Gray Level Variance
2520	δ-wavelet-LLL	P	δ-GLRLM	δ-High Gray Level Run Emphasis
2531	δ-wavelet-LLL	P	δ-GLRLM	δ-Short Run High Gray Level Emphasis
2855	δ-wavelet-LHH	P	δ-First-order	δ-Mean
3077	δ-wavelet-HLH	P	δ-GLDM	δ-Dependence Non Uniformity Normalized
3406	Deep-learning-based feature extraction	/	Non-delta	Classic-DL-X02
3455	Deep-learning-based feature extraction	/	Delta	Delta-DL-Z01

Ranked by ReliefF

Code	Image type	Peri/Intratumor	Feature class	Feature name
806	wavelet-LLL	I	GLDM	Gray Level Variance
1450	wavelet-HHL	P	GLCM	Difference Variance
1967	δ-wavelet-LHL	I	δ-GLRLM	δ-Run Entropy
1502	wavelet-HHL	P	GLSZM	Large Area Low Gray Level Emphasis
762	wavelet-LLL	I	First-order	Entropy
480	wavelet-HLH	I	First-order	10 Percentile
3449	Deep-learning-based feature extraction	/	Delta	Delta-DL-Y05
721	wavelet-HHH	I	GLDM	Small Dependence Low Gray Level Emphasis
1110	wavelet-LHL	P	GLRLM	Gray Level Variance
1636	wavelet-LLL	P	GLCM	Difference Variance
1860	δ-wavelet-LLH	I	δ-GLDM	δ-Large Dependence High Gray Level Emphasis
2317	δ-wavelet-HHL	I	δ-GLDM	δ-Dependence Entropy
1769	δ-original	I	δ-GLDM	δ-Low Gray Level Emphasis
1139	wavelet-LHL	P	GLSZM	Zone Variance
1116	wavelet-LHL	P	GLRLM	Run Entropy
2241	δ-wavelet-HLH	I	δ-GLRLM	δ-High Gray Level Run Emphasis
2470	δ-wavelet-LLL	I	δ-First order	δ-Median
1698	wavelet-LLL	P	NGTDM	Busyness
3456	Deep-learning-based feature extraction	/	Delta	Delta-DL-Z05
2132	δ-wavelet-HLL	I	δ-GLDM	δ-Dependence Non Uniformity
1708	δ-original	I	δ-shape	δ-Maximum 2D Diameter Row
1230	wavelet-LHH	P	GLSZM	Zone Entropy
1700	wavelet-LLL	P	NGTDM	Complexity
1728	δ-original	I	δ-First order	δ-Range
154	wavelet-LLH	I	GLDM	Gray Level Non Uniformity
3411	Deep-learning-based feature extraction	/	Classic	Classic-DL-X07
1257	wavelet-HLL	P	GLCM	Cluster Prominence
1471	wavelet-HHL	P	GLDM	Gray Level Variance
1857	δ-wavelet-LLH	I	δ-GLDM	δ-Gray Level Variance
2471	δ-wavelet-LLL	I	δ-First order	δ-Minimum
1552	wavelet-HHH	P	GLCM	Joint Energy
969	wavelet-LLH	P	First order	Minimum
749	wavelet-HHH	I	GLSZM	Small Area High Gray Level Emphasis
2021	δ-wavelet-LHH	I	δ-GLCM	δ-Difference Entropy
1000	wavelet-LLH	P	GLCM	Sum Squares
1150	wavelet-LHH	P	First order	Kurtosis
1115	wavelet-LHL	P	GLRLM	Low Gray Level Run Emphasis
492	wavelet-HLH	I	First order	Robust Mean Absolute Deviation

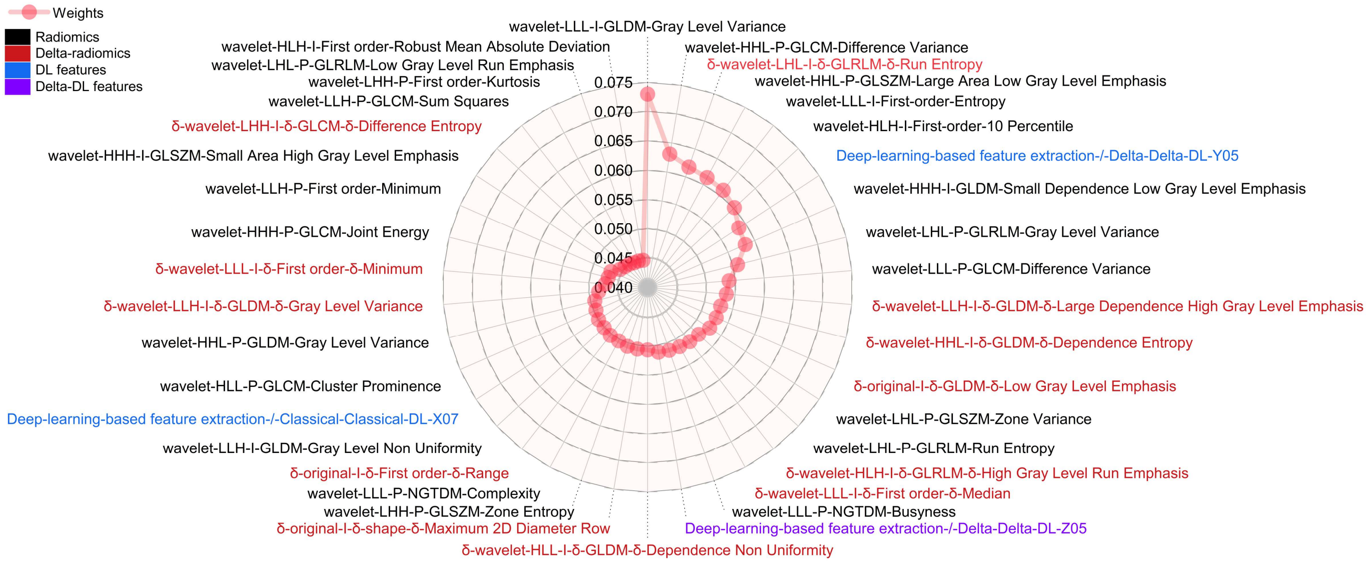

Supplementary Figure N1 | The importance of features in the prediction model calculated and ranked by ReliefF algorithm.

We further give the result of AUC metrics and the feature selection results provided by two algorithms (LASSO and ReliefF) of models trained with cohort of different follow-up lengths in **Supplementary Figure N2** below, which demonstrates that the delta-DL model might have more feature effectiveness when follow-up times were longer. The AUC for STAS diagnosis using delta-DL modality alone gradually improved when follow-up time became longer, and it eventually surpassed delta-radiomics in Group C with longer follow-up intervals. The core set of features provided by the LASSO and ReliefF algorithms had an increasing inclusion rate of delta-DL modality features when follow-up intervals increased, with a decreasing proportion of delta-radiomics features.

Items	Group A-short interval	Group B-medium interval	Group C-long interval
AUC metrics	Pure delta-DL 0.75<br>Classic DL 0.79<br>Pure delta-radiomics 0.78<br>Classic radiomics 0.80	Pure delta-DL 0.80<br>Classic DL 0.80<br>Pure delta-radiomics 0.84<br>Classic radiomics 0.79	Pure delta-DL 0.83<br>Classic DL 0.81<br>Pure delta-radiomics 0.81<br>Classic radiomics 0.82
Proportions in the core signature (identified by LASSO)	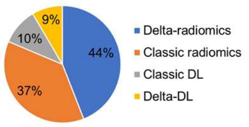	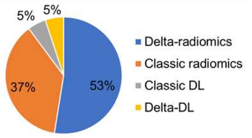	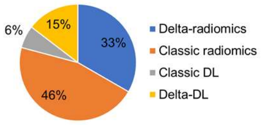
Proportions in the features with top 100 importance (given by ReliefF)	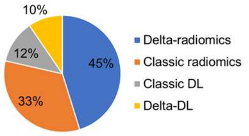	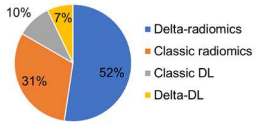	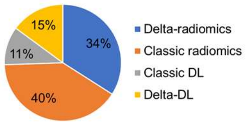

Supplementary Figure N2 | Supportive information for that the delta-DL model might have more feature effectiveness when follow-up times were longer. The AUC metrics, proportions in the core signature (identified by LASSO, and proportions in the features with top 100 importance (given by ReliefF) of models trained with cohort of different follow-up lengths.

Appendix O| On the sample size evaluation

A sample size of at least 37 cases (28 STAS-positive and 9 STAS-negative) was required in the training and test cohorts based on the following inputs and assumptions: power, 80%; two-sided significance level, 0.05; alternative hypothesis of the area under the receiver operating characteristic (ROC) curve (AUC), 0.800, compared with the null hypothesis of the AUC, 0.500; and an allocation ratio of sample sizes in the negative and positive groups of CHEST Cohort. Thus, sample sizes of 509 with 119 STAS-positive and 76 with 24 STAS-positive in the training cohort and the in-center validation cohort were sufficient to detect an AUC difference of 0.500 with 80% power if the true AUC was above 0.800. Statistical analyses were performed using MedCalc (version 20.218; https://www.medcalc.org).

Appendix P| On the bias of pathological types

We analyzed the possible bias of pathological types from two different perspectives: for one, we analyzed the predictive accuracy of the model on various major pathology types, namely lung adenocarcinoma (LUAD), lung squamous carcinoma (LUSC), and other relatively rare pathology types; for another, in different pathology grades of LUAD, we also analyzed the predictive performance of the model.

Our results suggest that the model maintained a basically consistent prediction accuracy across major pathology types and pathological grades in LUAD (shown in **Supplementary Figure P**). Therefore, the pathology subtypes and grades may not be a major classification bias. However, considering that the training process of the model included mainly patients with LUAD, bias between different pathologic types may still exist. Conservatively, it is still necessary to incorporate richer pathologic types into the training and validation of the model in the future to ensure that pathologic subtypes do not become the main bias.

As for the definition of LUAD grades in **Supplementary Figure P**), the new grading system for invasive lung adenocarcinoma introduced here was proposed by the Pathology Committee of the International Association for the Study of Lung Cancer (IASLC) in 2020 and was adopted by the 2021 edition of the WHO Classification of Thoracic Tumors. The system establishes grading criteria that are closely related to patient prognosis based on the five growth modes of invasive lung adenocarcinoma and a combination of tumor cell characteristics and other high-risk factors. The new grading system defines 20% of high-grade component as the threshold value and combines it with the diagnostic model of invasive lung adenocarcinoma of the predominant growth mode type in the 2015 edition of the WHO lung cancer classification to classify invasive lung adenocarcinoma into Grade 1 (predominantly lepidic, without or with <20% of high-grade component), Grade 2 (predominantly vesicular or papillary, with no or <20% of high-grade component) and Grade 3 (predominantly any growth pattern with ⩾20% high-grade component). The new grading system incorporates a new high-grade type, complex glandular, in addition to the original high-grade types, solid and micropapillary. Studies have shown that these high-grade types are strongly associated with poor patient prognosis.

We would also like to point out in detail that the training process in **Supplementary Figure P-A** and **Supplementary Figure P-B** is that for **Supplementary Figure P-A**, since there are sufficient numbers of patients with Grades of I, II and III, respectively, we trained the model mainly in patients of other grades, and externally validated it in patients of the pathology grade of interest. Therefore, the ROC curves and AUC values in **Supplementary Figure P-A** are the corresponding validation results; For **Supplementary Figure P-B**, since the main data source of the model is from LUAD, the model behaves as underfitting when trained purely on patients of other pathological types due to the insufficient training data, which makes the corresponding validation results less meaningful. Therefore, we trained the model on LUAD patient data and validated it on LUSC and other rare pathology types; the LUAD-ROC in **Supplementary Figure P-B** is the result of the 10-fold cross-validation in LUAD patients, while the ROC curves for LUSC and other pathology types are the results of the validation of the model trained on LUAD patient data in these patients.

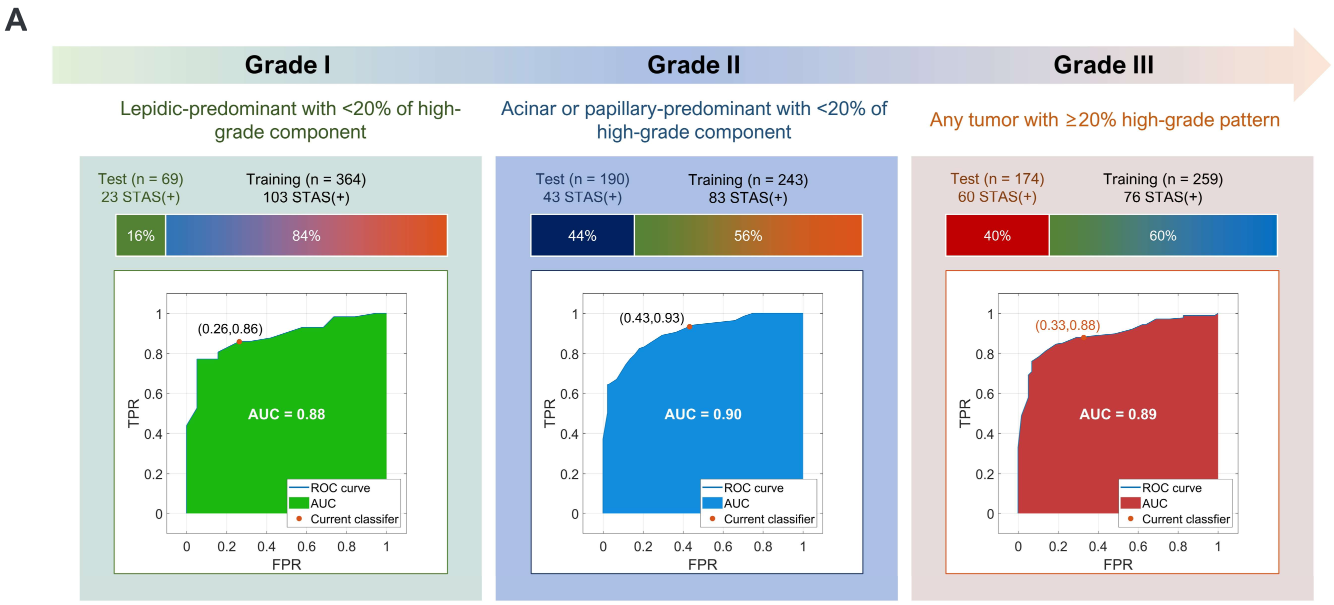

1. High-grade pattern/component includes solid (S), micropapillary (MP), and complex glandular pattern (CGP, including cribriform as nests of tumor cells with sieve-like perforation and fused gland as fused glands with irregular borders, back-to-back glands without intervening stroma, or ribbon-like formations).
2. New IASLC grading system is not applicable to invasive mucinous adenocarcinoma. Therefore, 433 patients (126 STAS-positive) with non-mucinous IAC were included in this analysis.

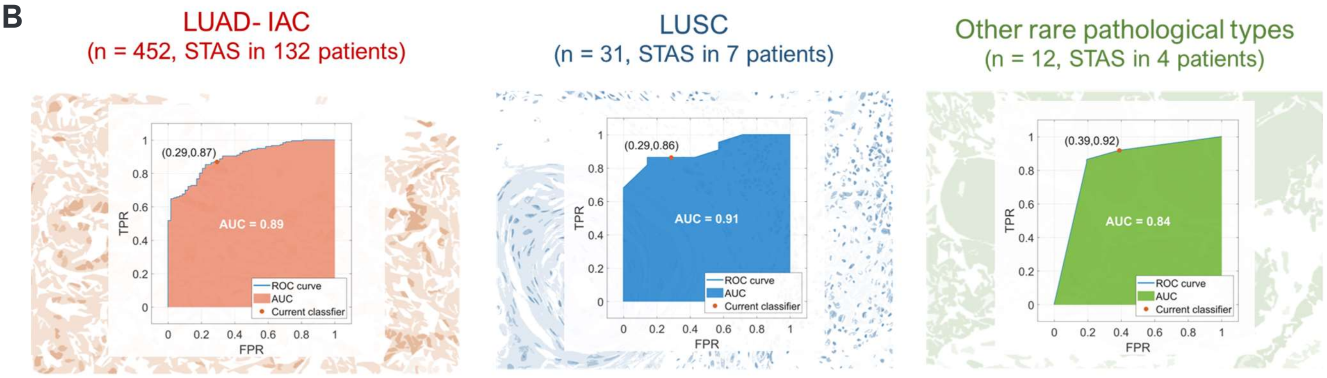

Supplementary Figure P | The sub-group/stratified analysis by different pathological types and grades. A. Predictive accuracy of different pathologic grades in lung adenocarcinoma (LUAD). **B.** Predictive accuracy of different major pathologic types (adenocarcinoma, squamous carcinoma, and other rare pathological types).

SI References

1. Jiang C, Luo Y, Yuan J, et al. CT-based radiomics and machine learning to predict spread through air space in lung adenocarcinoma. *Eur Radiol*. 2020;30(7):4050-4057. doi:10.1007/s00330-020-06694-z
2. Liu Q, Qi W, Wu Y, Zhou Y, Huang Z. Construction of Pulmonary Nodule CT Radiomics Random Forest Model Based on Artificial Intelligence Software for STAS Evaluation of Stage IA Lung Adenocarcinoma. *Comput Math Methods Med*. 2022;2022. doi:10.1155/2022/2173412
3. Onozato Y, Nakajima T, Yokota H, et al. Radiomics is feasible for prediction of spread through air spaces in patients with nonsmall cell lung cancer. *Sci Rep*. 2021;11(1):1-10. doi:10.1038/s41598-021-93002-4
4. Bassi M, Russomando A, Vannucci J, et al. Role of radiomics in predicting lung cancer spread through air spaces in a heterogeneous dataset. *Transl Lung Cancer Res*. 2022;11(4):560-571. doi:10.21037/tlcr-21-895
5. Chen D, She Y, Wang T, et al. Radiomics-based prediction for tumour spread through air spaces in stage i lung adenocarcinoma using machine learning. *European Journal of Cardio-thoracic Surgery*. 2020;58(1):51-58. doi:10.1093/ejcts/ezaa011
6. Takehana K, Sakamoto R, Fujimoto K, Matsuo Y, Nakajima N. Peritumoral radiomics features on preoperative thin - slice CT images can predict the spread through air spaces of lung adenocarcinoma. *Sci Rep*. 2022;(0123456789):1-9. doi:10.1038/s41598-022-14400-w
7. Han X, Fan J, Zheng Y, et al. The Value of CT-Based Radiomics for Predicting Spread Through Air Spaces in Stage IA Lung Adenocarcinoma. *Front Oncol*. 2022;12(July):1-11. doi:10.3389/fonc.2022.757389
8. Qi L, Li X, He L, et al. Comparison of Diagnostic Performance of Spread Through Airspaces of Lung Adenocarcinoma Based on Morphological Analysis and Perinodular and Intranodular Radiomic Features on Chest CT Images. *Front Oncol*. 2021;11(June):1-11. doi:10.3389/fonc.2021.654413
9. Liao G, Huang L, Wu S, et al. Preoperative CT-based peritumoral and tumoral radiomic features prediction for tumor spread through air spaces in clinical stage I lung adenocarcinoma. *Lung Cancer*. 2022;163:87-95. doi:10.1016/j.lungcan.2021.11.017
10. Tao J, Liang C, Yin K, Fang J, Chen B, Wang Z. 3D convolutional neural network model from contrast-enhanced CT to predict spread through air spaces in non-small cell lung cancer. *Diagn Interv Imaging*. 2022;000. doi:10.1016/j.diii.2022.06.002
11. Jolly YYBMP. for Optimal Boundary & Region Segmentation of Objects in N-D Images. In: *Proceedings Eighth IEEE International Conference on Computer Vision(ICCV 2001)*. Vol 1. ; 2001:105-112.
12. Teuwen J, Gouw ZAR, Sonke JJ. Artificial Intelligence for Image Registration in Radiation Oncology. *Semin Radiat Oncol*. 2022;32(4):330-342. doi:10.1016/j.semradonc.2022.06.003
